# Neutrophil extracellular traps (NET) induced by different stimuli: A comparative proteomic analysis

**DOI:** 10.1371/journal.pone.0218946

**Published:** 2019-07-08

**Authors:** Andrea Petretto, Maurizio Bruschi, Federico Pratesi, Cristina Croia, Giovanni Candiano, Gianmarco Ghiggeri, Paola Migliorini

**Affiliations:** 1 Core Facilities-Proteomics Laboratory, Istituto Giannina Gaslini, Genoa, Italy; 2 Laboratory of Molecular Nephrology, Istituto Giannina Gaslini, Genoa, Italy; 3 Clinical Immunology Unit, Department of Clinical and Experimental Medicine, University of Pisa, Pisa, Italy; 4 Division of Nephrology, Dialysis, and Transplantation, Scientific Institute for Research and Health Care (IRCCS), Istituto Giannina Gaslini, Genoa, Italy; Hospital for Sick Children, CANADA

## Abstract

Neutrophil extracellular traps (NET) formation is part of the neutrophil response to infections, but excessive or inappropriate NETosis may trigger the production of autoantibodies and cause organ damage in autoimmune disorders. Spontaneously netting neutrophils are not frequent and induction of NET *in vitro* by selected stimuli is necessary to investigate their structure. In the present work, the protein composition and post-translational modifications of NET produced under different stimuli have been studied by means of proteomic analysis. Neutrophils from healthy donors were stimulated by PMA, A23187, *Escherichia coli* LPS or untreated; after three hours, cells were washed, treated with DNase and supernatants collected for mass spectrometry. Data were analyzed by unsupervised hierarchical clustering analyses. We identified proteins contained in NETs of any source or exclusive of one stimulus: LPS-induced and spontaneous NET diverge in protein composition, while PMA- and A23187-induced NET appear more similar. Among the post-translational modifications we examined, methionine sulfoxidation is frequent especially in PMA- and LPS-induced NETs. Myeloperoxidase is the protein more extensively modified. Thus, proteomic analysis indicates that NETs induced by different stimuli are heterogeneous in terms of both protein composition and post-translational modifications, suggesting that NET induced in different conditions may have different biological effects.

## Introduction

Neutrophil extracellular traps (NET) formation (NETosis) is a mechanism of defense that neutrophils deploy as an alternative to phagocytosis, to constrain the spread of fungi, large bacteria and several other microorganisms [1,2]. During NETosis, nucleus decondenses and intracellular membranes disintegrate; nuclear and cytoplasmic content mixes and plasma membrane permeabilizes, allowing the extrusion of a tangle of chromatin fibers decorated with granule proteins. NET trap and kill infectious agents due to the high local concentration of histones, anti-microbial peptides and other anti-microbial agents. Neutrophils are induced to release NET by the engagement of a variety of receptors by microbial products, immune complexes and crystals. In the best characterized pathway leading to NET extrusion, ERK signaling leads to the activation of NADPH oxidase and the production of superoxide radicals that are converted into oxygen peroxide by superoxide dismutase [3]. Myeloperoxidase (MPO) transforms hydrogen peroxide in hypochlorous acid, activating neutrophil elastase (NE). NE is responsible for degradation of cytoskeleton and nuclear membrane, allowing the mixing of nuclear content and cytoplasm [4]. Histone deimination by activated peptidylarginine deiminase (PAD) and proteolytic cleavage by MPO and NE lead to chromatin decondensation [5]. Chromatin fibers associate with granule and cytoplasmic proteins and are then released extracellularly. Transcriptional firing at multiple locations contributes to chromatin decondensation [6], but NET formation proceeds independently of translation [7].

Reactive oxygen species (ROS) production is the key event in NETosis and both mitochondrial respiratory chain and NADPH oxidase independently contribute to their generation. Several different receptors trigger NET formation activating NADPH oxidase (NOX), in the classical “suicidal” NETosis [8]. Similarly, phorbol myristate acetate (PMA), resembling diacylglycerole, activates classical and conventional phosphokinase C (PKC) [9] and ERK signaling, mimicking NET induction by bacteria and fungi. PMA is in fact a very efficient and very frequently used stimulus, even if experimental protocols of NET induction via PMA may substantially differ [10].

NOX independent NETosis relies on mitochondrial ROS production, facilitated by alkaline pH that increases Ca influx [11]. Triggering of a calcium-activated small conductance potassium (SK) channel member SK3 is critical for calcium activated NOX-independent NETosis [12].

PAD4 activation is prominent in this condition and citrullination of histones is extensive. Calcium ionophores like ionomycin (product of some Gram-positive bacteria like *Streptomyces)* and A23187 activate PKC-ζ and PAD4 [9], inducing NOX-independent NETosis. In some conditions, NOX-independent nuclear and mitochondrial DNA release takes place from vital neutrophils. It has been shown that immune complexes, acting on normal or low-density granulocytes, induce mitochondrial ROS production and extrusion of NET containing mitochondrial DNA from alive cells [13]. Similarly, activated platelets in sepsis patients adhere to neutrophils and induce the extrusion of NET from live cells [14].

Spontaneously netting neutrophils, even in condition where NETosis is increased, are not so frequent to allow NET isolation and characterization. Thus, induction of NET in vitro from isolated neutrophils is a pre-requisite to investigate their physiological and pathological role. To such extent, different stimuli have been employed, such as bacterial products (lipopolysaccharide-LPS), calcium ionophores or PMA (recently reviewed by Hoppenbrowers *et al*, 10).

Twenty-five different NETosis inducers have been reported, and many of them at different concentrations or for different times [10]. Subtle procedural differences may lead to different results, complicating any attempt to build up a common core of knowledge on NETosis [15]. Moreover, non-overlapping pathways of NET induction are triggered by different stimuli, as recently shown by means of selective inhibitors [16]. Despite a different role of ROS production and activity of granule enzymes in NET induction, the different pathways lead to the production of NETs endowed with a similar bactericidal activity [16].

Nonetheless, the possibility that NET induced by different stimuli differ in protein composition has never been thoroughly analyzed.

The aim of the present work is to investigate the protein composition and post-translational modifications of NET produced under different stimuli by means of proteomic analysis.

## Results

### Proteins composition of NETs

We analyzed the protein composition of NETs, produced *in vitro* by isolated neutrophils, either spontaneously or after stimulation with lipopolysaccharide (LPS), calcium ionophore A23187 and phorbol 12-myristate 13-acetate (PMA).

PMA was the most effective stimulus for the induction of NETs (S1 Fig) and no stimulus induced neutrophil apoptosis (S2 Fig).

**Fig 1:**
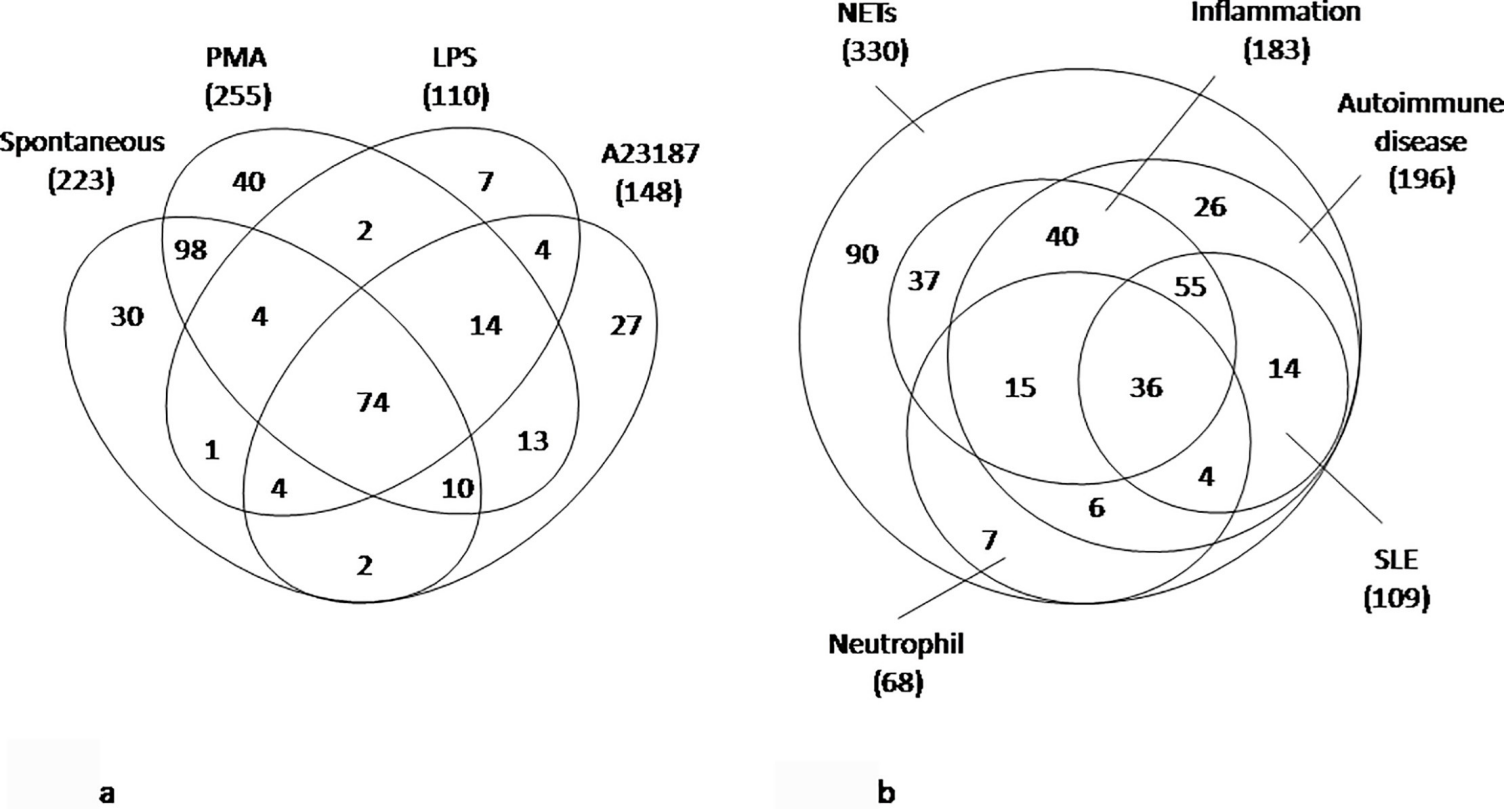
Venn diagram of proteins identified in NETs. A. Venn diagram of overlapped and unique NETs proteins identified by means of mass spectrometry. Number of proteins in the overlapping and not-overlapping areas is shown. B. Venn diagram of NETs proteins grouped on the basis of database annotation. Number of proteins in the overlapping and not-overlapping areas is shown.

A total of 330 proteins were identified as NET constituents. Among these, 74 proteins (22%) were detected in NETs of any source while 30 (9.1%), 7 (2.1%), 27 (8.2%) and 40 (12.1%) proteins were exclusive, respectively, of spontaneous NETs, or NETs obtained with LPS, A23187 and PMA (Fig 1A and S1 Table).

The majority of NET proteins corresponded to proteins annotated to inflammation (183), autoimmunity disease (196) and in particular to SLE (109) in several databases (UniProt, Open Target and Atlas) (Fig 1B).

**Fig 2:**
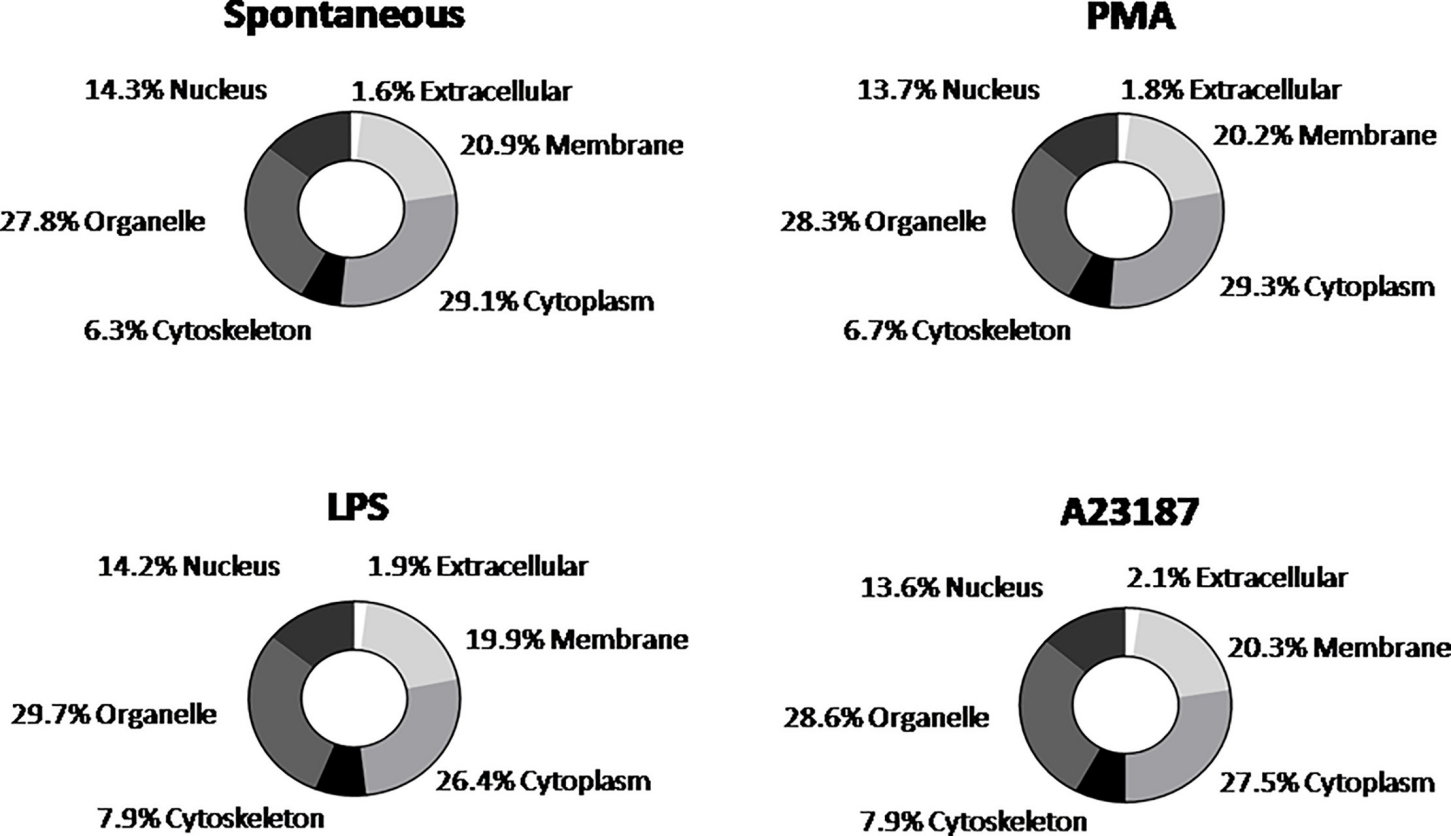
Pie chart of cellular component gene ontology annotation of NETs samples.

Cellular origin of NET constituents was very similar in NETs of any origin, with a range of 1.6–2.1% (extracellular), 19.9–20.9% (membrane), 34.3–36% (cytoplasm/cytoskeleton), 27.8–29.7% (organelle) and 13.6–14.3% (nucleus) (Fig 2).

**Fig 3:**
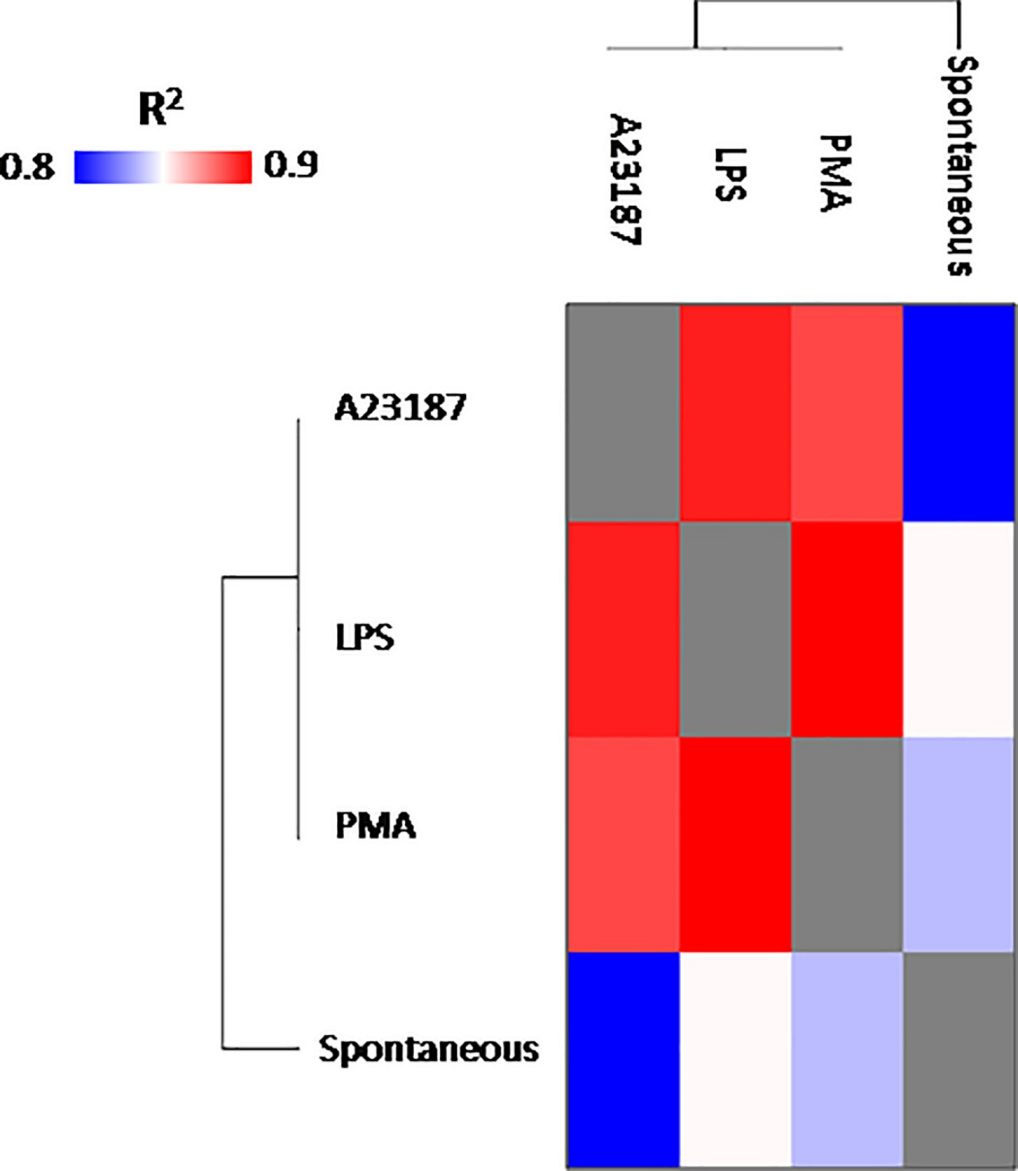
Spearman’s correlogram associated to unsupervised hierarchical clustering analysis of NETs. In Spearman’s correlogram the coefficient values are depicted by a pseudo color scale from 0.8 (blue) to 0.9 (red); moreover the tree dendrogram displays the results of an unsupervised hierarchical clustering analysis placing similar Spearman's coefficient values near each other.

The vast majority of NET proteins are shared by spontaneous NETs and by NETs obtained under PMA, LPS and A23187 stimulation. However, by clustering analysis a core of proteins allowing a good discrimination between spontaneous and stimuli-induced NETs can be identified (Fig 3).

**Fig 4:**
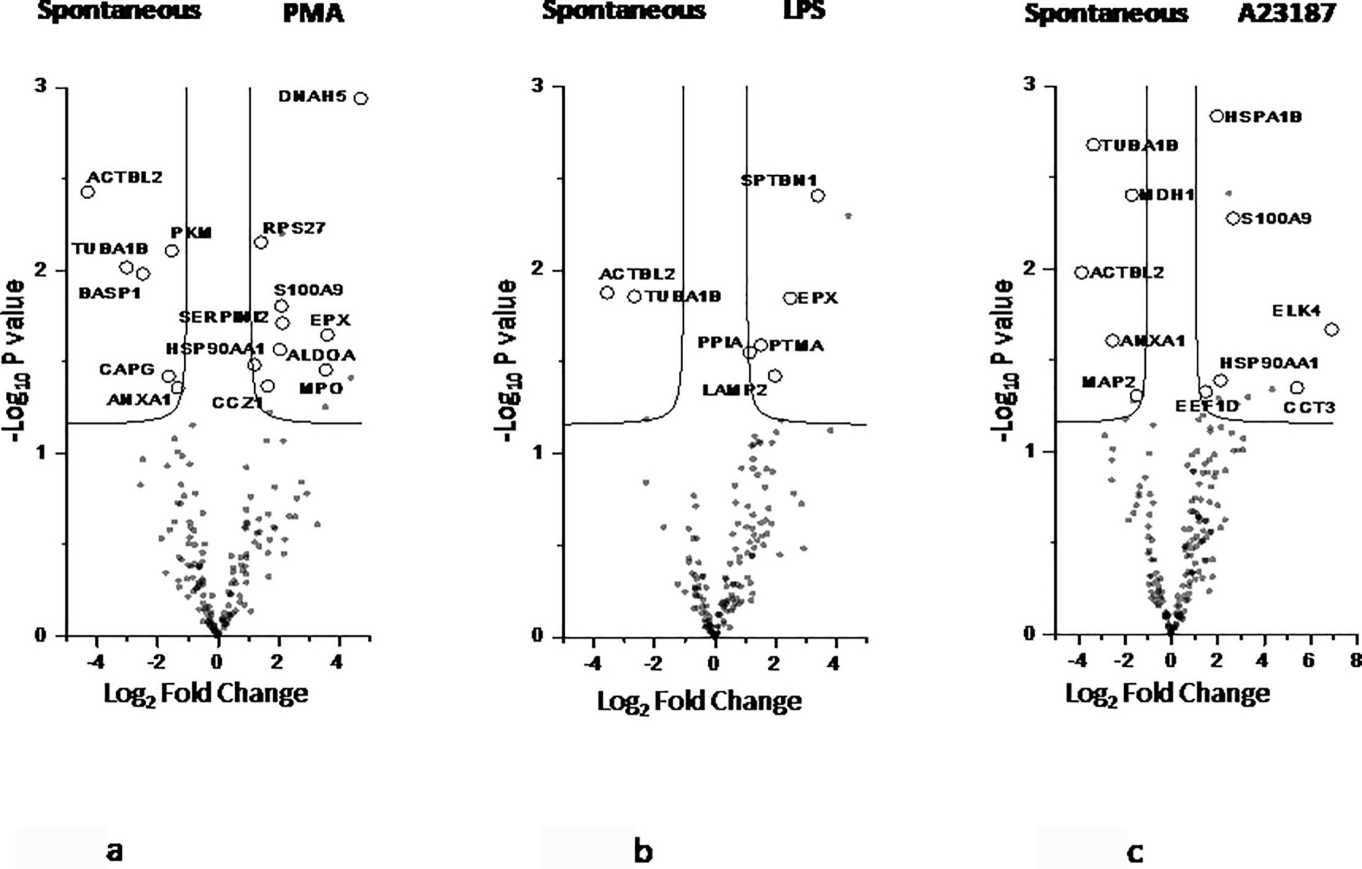
Volcano plot of univariate statistical analysis of NETs samples. Volcano plot based on fold change (Log_2_) and P value (-Log_10_) of all the proteins identified in PMA (4a), LPS (4b) and A23187 (4c) as compared with spontaneous NETs. White circles indicate the proteins displaying statistically significant changes.

To better describe the differences between spontaneous and stimuli-induced NETs, univariate statistical analysis, PLS-DA and SVM were performed. A total of 25 proteins were highlighted. Among these, 9, 5 and 6 proteins are enriched respectively in PMA-, LPS- and A23187-induced NETs (right side of each volcano plot, Fig 4).

**Fig 5:**
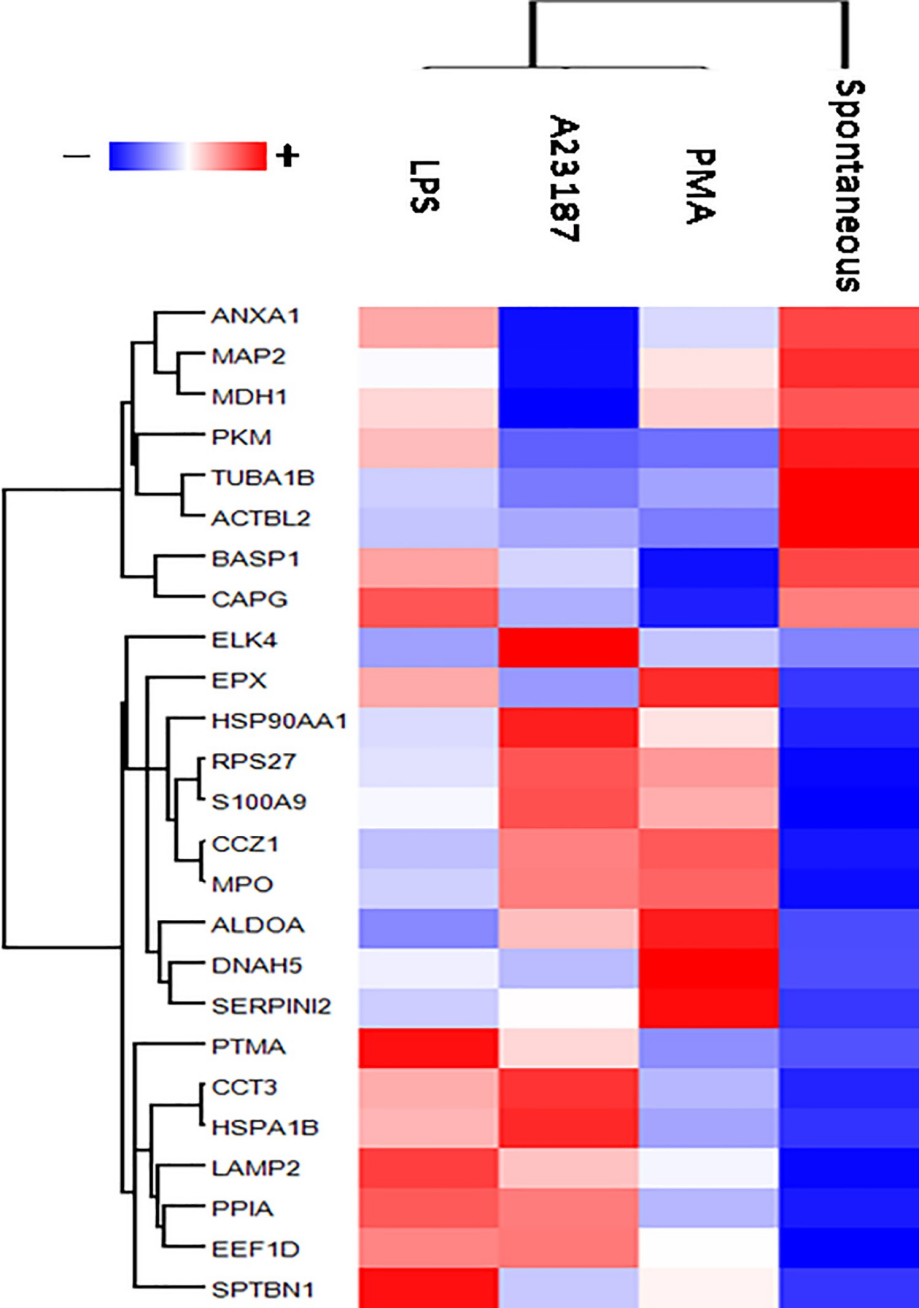
Heat map of NETs highlighted proteins. Heat map of proteome profile of 25 proteins highlight through the combined use of univariate statistical analysis, support vector machine and partial least square discriminant analysis. In heat map, each row represents a protein, and each column corresponds to each condition. Normalized Z-score of proteins abundance are depicted by a pseudo color scale with red indicating positive expression, white equal expression, and blue negative expression compared to each proteins values, whereas the tree dendrogram displays the results of an unsupervised hierarchical clustering analysis placing similar proteome profile values near each other. Visual inspection of the dendrogram and heat map demonstrate the possibility to distinguish among the different condition of NETs induction.

The proteome profile of the 25 highlighted proteins is shown by means of heat map after Z-score (Fig 5).

**Fig 6:**
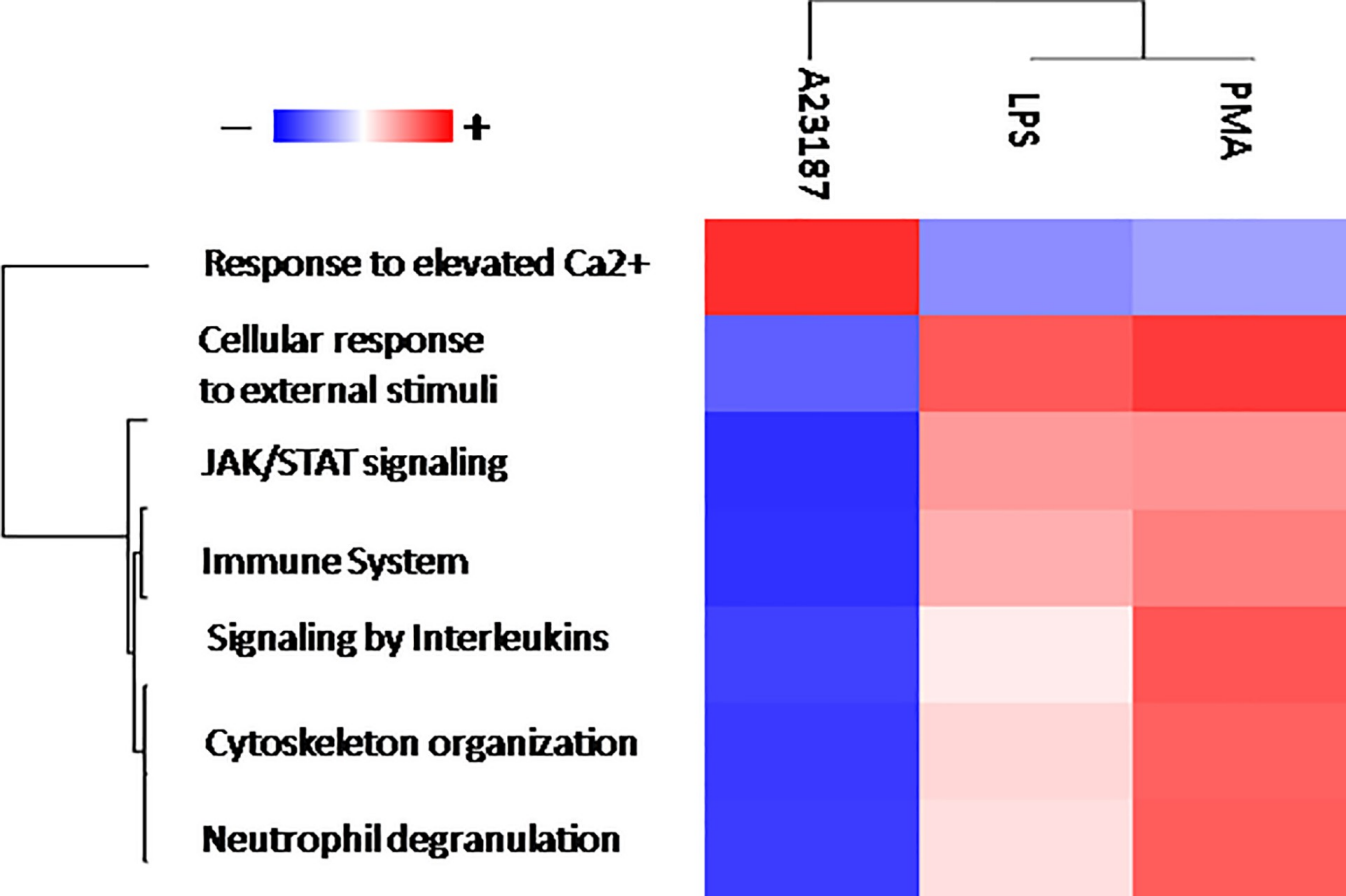
Heat map of NETs gene ontology enrichment analysis. Heat map of biological process enrichment of different NETs stimuli compared to no stimulated condition. In the heat map, each row represents a protein, and each column corresponds to each condition. Normalized Z-score of proteins abundance are depicted by a pseudo color scale with red indicating positive expression, white equal expression, and blue negative expression compared to each proteins values, whereas the tree dendrogram displays the results of an unsupervised hierarchical clustering analysis placing similar proteome profile values near each other. Visual inspection of the dendrogram and heat map demonstrate the ability of these biological processes to distinguish between the A23187 and PMA/LPS stimuli.

These 25 proteins were classified according the available gene ontology (GO) signatures in cellular component (CC), molecular function (MF), and biological process (BP). Among CC proteins, 50% were annotated as cytoplasm/cytoskeleton, 20% as nucleus, 19% as membrane and 11% as extracellular. Based on MF, 50% proteins were classified as “binding proteins”, 23% as “catalytic activity”, 20% as “structural molecule” and 6% as “antioxidant activity”. In the BP, most proteins were involved in cellular component rearrangement, development process and response to external stimuli. The diversity of the protein expression profile between spontaneous and stimuli-induced NETs suggests an involvement of these proteins in different biological pathways/processes. To assess this, we performed gene ontology (GO) analysis based on gene annotation extracted from different open source databases. Using all identified proteins and their abundance, we highlighted the signatures of each condition. To summarize the results of GO analysis we drew a heat map of enriched gene signatures (Fig 6).

This diagram reports the average of proteins expression associated to each highlighted biological process (rows) in the different stimuli (columns). In PMA- and LPS-induced NETs, proteins associated with cellular response to external stimuli, immune system response, neutrophil degranulation, cytoskeleton rearrangement, interleukin signalling and in JAK/STAT pathwaywere positively enriched. Proteins involved in the response to elevated Ca^2+^ are instead up regulated in A23187-induced NETs.

### Post translational modifications (PTM) of NET components

**Fig 7:**
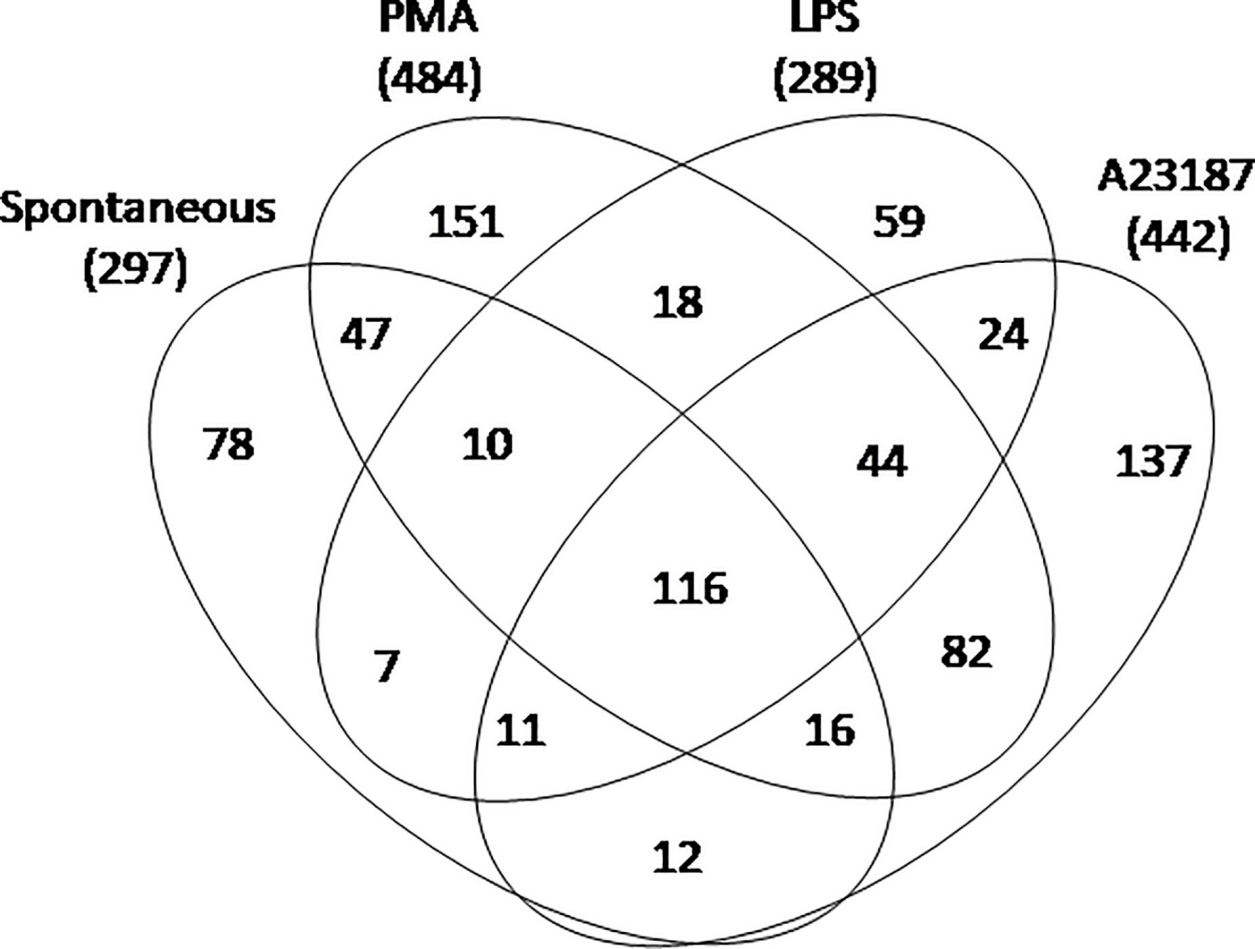
Venn diagram of total NETs peptides with at least one post translational modification identified by means of mass spectrometry. Venn diagram shows overlapped and unique peptides. Numbers represent the distinct peptides/post translational modifications in the overlapping and not-overlapping areas.

We evaluated a group of PTM potentially relevant in NETosis: methionine oxidation (sulfoxide), thiol alkylation (addition of iodoacetamide), deamination of N and Q amino acids, deimination of arginine (citrullination) and all other types of PTM grouped as “others”. A total of 812 peptides with at least one PTM were identified. The majority of PTM were found in PMA-induced NETs (484) followed by A23187 (442), LPS (289) and spontaneous NETs (297) (Fig 7 and S2 Table).

Most of these PTM are shared by different stimuli, but a few were exclusive for each condition. In particular, 78 (9.6%), 59 (7.3%), 137 (16.9%) and 151 (18.6%) PTM were exclusive respectively for spontaneous, LPS-, A23187- and PMA-induced NETs (Fig 7).

Spontaneous NETs display a prevalence of thiol alkylation, whereas PMA- and LPS-induced NETs exhibit a prelevance of methionine sulfoxidation and N and Q deamination.

To better describe the differences between spontaneous and stimuli-induced PTM of NET proteins, univariate statistical analysis, PLS-DA and SVM were performed.

**Fig 8:**
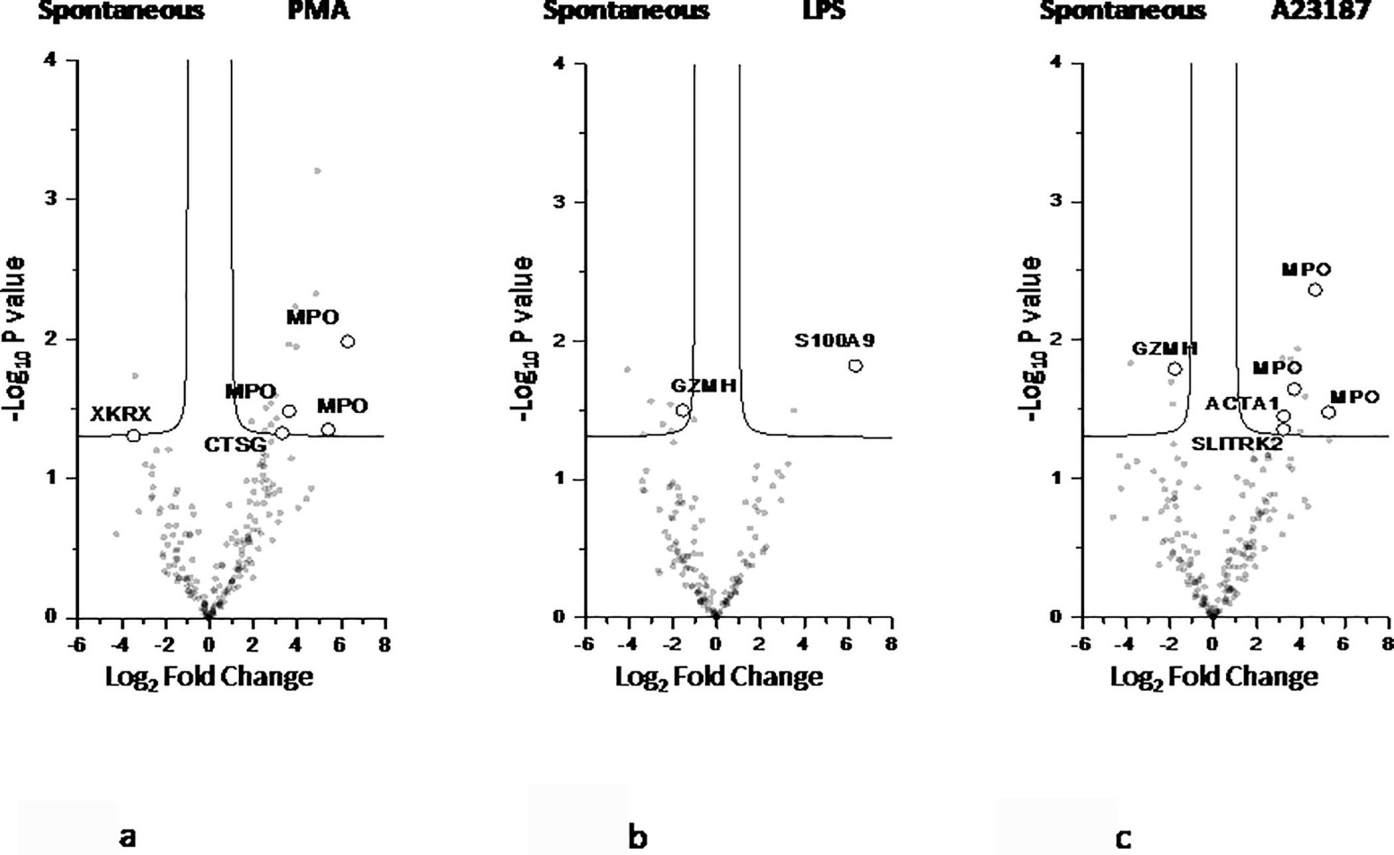
Volcano plot of univariate statistical analysis of spontaneous and induced NETs. Volcano plot based on fold change (Log_2_) and P value (-Log_10_) of all the peptides with at least one PTM identified in PMA (8a), LPS (8b) and A23187 (8c) as compared with spontaneous NETs. White circles indicate the peptides displaying statistically significant changes.

A total of 11 peptides with at least one PTM, corresponding to 7 proteins, were significantly enriched across the different conditions (Fig 8).

**Fig 9:**
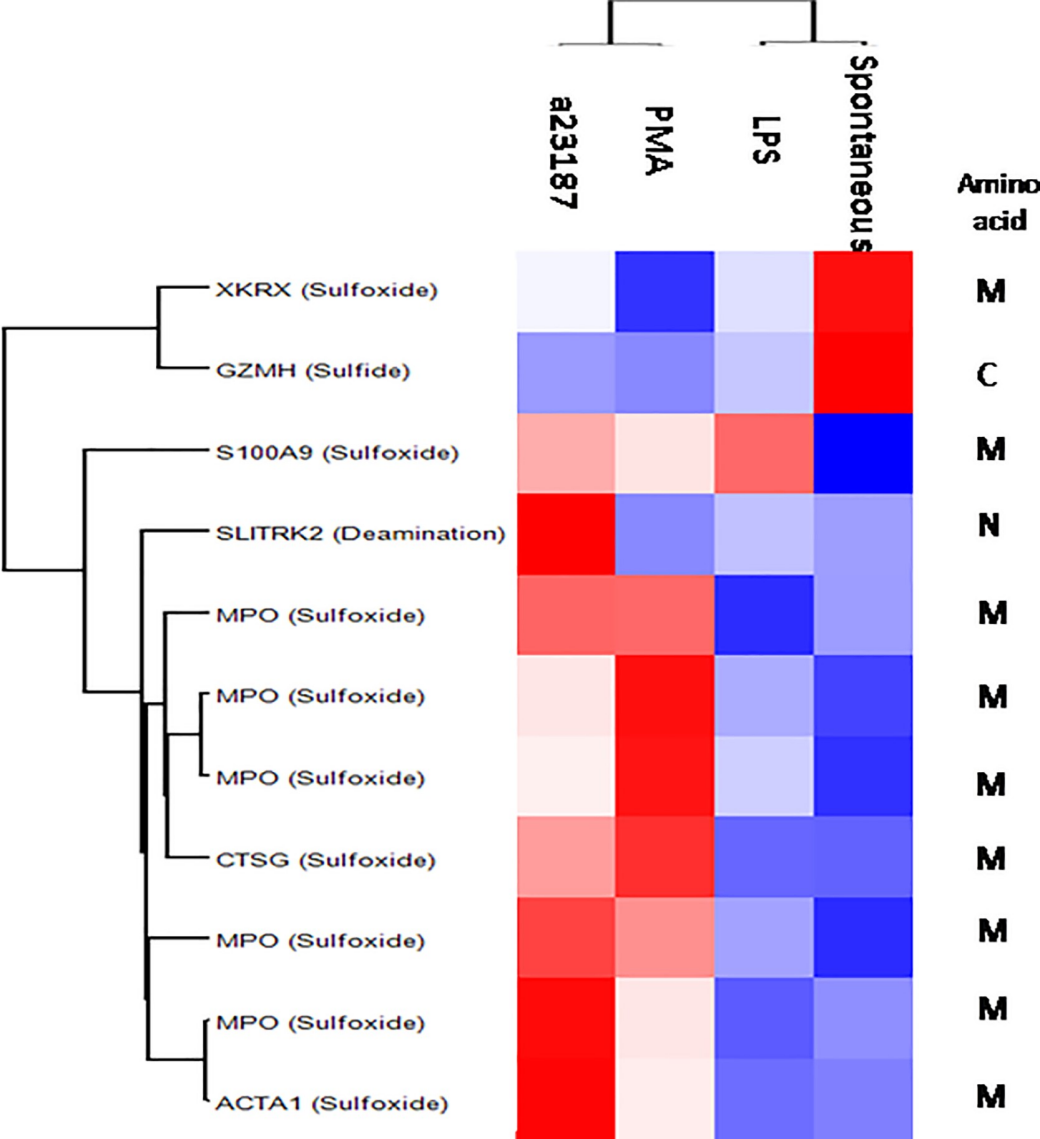
Heat map of highlight peptides. Heat map of peptide profile of NETs samples. In heat map, each row represents a modified peptide, and each column corresponds to each stimulus. Normalized Z-score of peptides abundance are depicted by a pseudo color scale with red indicating positive expression, white equal expression, and blue negative expression compared to each peptide value, whereas the tree dendrogram displays the results of an unsupervised hierarchical clustering analysis placing similar peptide profile values near each other.

The intensity profile of these highlighted peptides bearing PTM are show by means of heat map after Z-score (Fig 9).

Visual inspection of heat map shows the presence of two clusters composed by spontaneous and LPS-induced NETs versus A23187- and PMA-induced NETs. A23187, in particular, shows the highest intensity of peptides bearing PTM. The 7 highlighted proteins classified as CC components were equally distributed between extracellular and organelle proteins. Based on MF, the majority of proteins were classified as catalytic/antioxidant activity (40%), binding proteins and structural molecules (20% each). The principals BP involved were cellular component rearrangement, immune system process and response to external stimuli.

**Fig 10:**
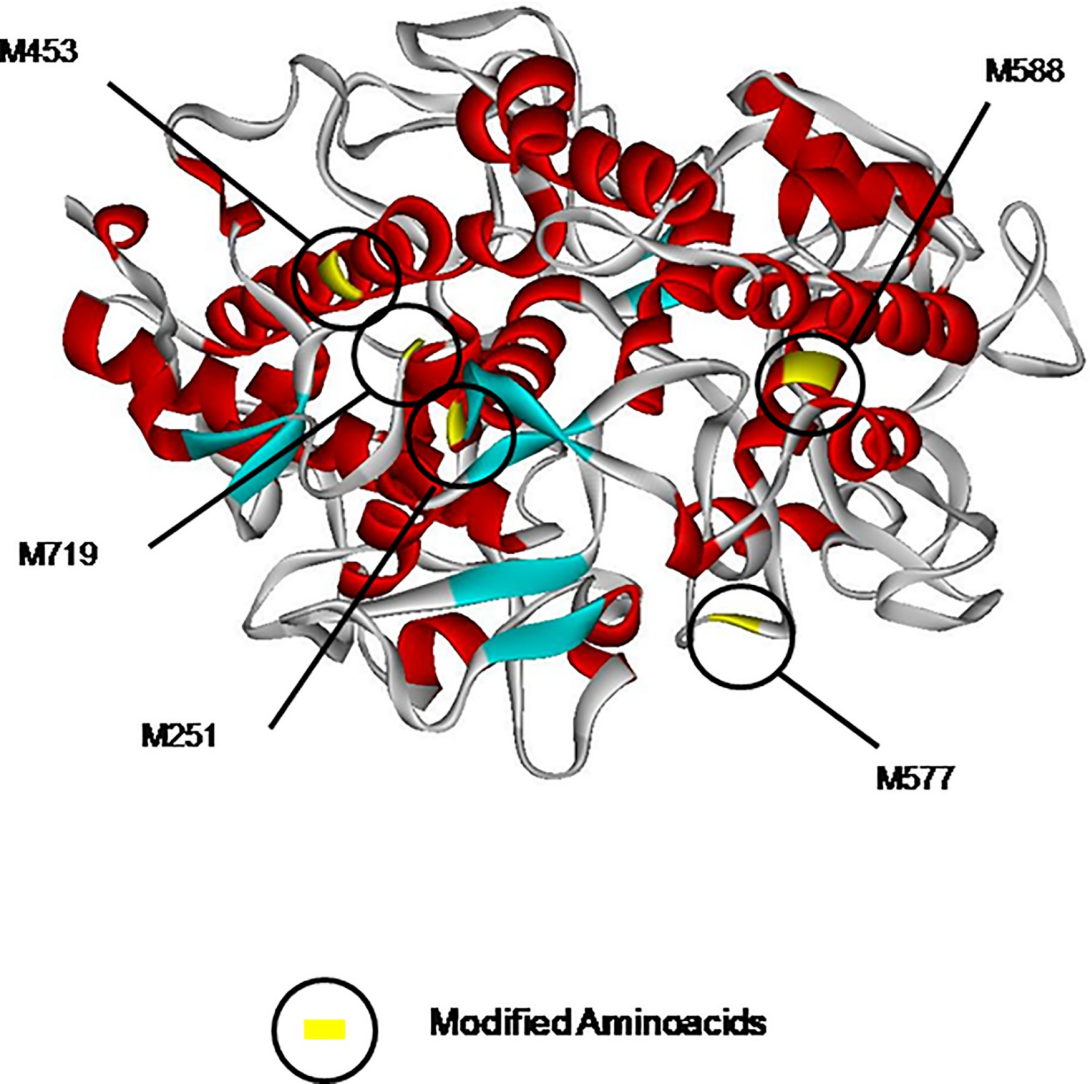
Three-dimensional (3D) model of MPO. 3D-model of MPO based on its crystal structure (PDB codes: 5 MFA). The model is colored in function of second structure i.e. gray, red and cyan respectively for coil, alpha helix and beta-strand. In yellow methionine sulfoxide is shown.

Interestingly, methionine sulfoxide is the most frequent PTM across all conditions of NET formation (82%) and the majority of modified peptides (5/11, 45%) derive from MPO. The position of the oxidized amino acid depends on the type of stimulus but all oxidized peptides are localized on the surface of molecule (Fig 10), suggesting that in the different pathways triggered by each stimulus MPO can be oxidized in different spots.

In NETs obtained with different stimuli, proteins differ also in PTM. PMA- and A23187-induced NETs are again similar, bearing a higher number of PTM when compared to LPS-induced and spontaneous NETs. These latter are characterized by a lower degree of oxidation: thiol alkylation, indicative of a free SH group, is in fact more frequent in spontaneous NETs. No major differences are observed in the frequency of the other PTM we studied, but individual proteins bearing PTM may be differentially enriched in NET obtained with the various stimuli: oxidized MPO and cathepsin G are abundant in PMA- and A23187-induced NET, oxidized calprotectin in A23187- and LPS-induced NET, while oxidized actin is highly represented in A23187-induced NET.

## Discussion

Despite the growing interest in NETosis as a double-edged sword in innate immunity, several issues are still obscure. Multiple receptors are engaged in NETosis and redundant signaling pathways are involved, thus allowing a fine tuning of this potentially dangerous tool.

Each of the stimuli we used for NET induction recapitulates the pathways physiologically triggered in neutrophils. The different pathways, however, converge and the final steps (degradation of nuclear membrane, chromatin decondensation, extrusion of fibers) are shared. As a consequence, NET produced by all stimuli are proteolytically active and kill bacteria [16]. Thus, it is conceivable that the vast majority of NET proteins are identical across stimuli and come from the same cell compartments. Proteomic analysis of NETs induced by different stimuli shows in fact a common core of proteins, which characterizes any type of NETs we studied and have been previously described as NET components [17, 18]. The presence in NETs of proteins contained in cytoplasm, nucleus, and membranes is consistent with the process of NET formation, which involves multiple pathways in several cellular compartments. Components from granules and cytoplasm in general are more abundant; histones represent the main nuclear proteins.

However, in the different conditions proteins differentially expressed were also detected, that can be ascribed to only partially overlapping pathways, related to the process of NET formation.

The heat map and the Volcano plot clearly depict these differences. On the whole, LPS-induced and spontaneous NET widely diverge in protein composition, while PMA- and A23187-induced NET appear more similar. In fact, ROS production is observed also in NOX-independent pathways [16], thus explaining why end products are similar in NOX dependent (PMA) or independent stimuli (A23187).

Taking into account individual components, cytoskeleton proteins such as actin and tubulin are enriched in spontaneous NET, while the dynein protein DNAH5 and the heat shock protein HSPA1B are highly represented in PMA-induced NET. Calprotectin S100A9 and the ribosomal protein RPS27 are shared by PMA- and A23187-induced NET, while LAMP2 (lysosomal associated membrane protein 2) is enriched in LPS-induced NET.

NETosis is critical in innate immunity and the failure to form NET is associated with a severe form of immunodeficiency [19]. Even if NETosis has been discovered and studied as a critical step in protection from infections, available data on the interaction of neutrophils and pathogens in NET formation are not conclusive. The role of LPS, a major component of the outer membrane of Gram-negative bacteria, as signaling molecule has been debated and conflicting data have been published. Recently, it has been suggested that only a few bacteria express LPS able to induce directly NETosis, by a TLR4 independent signaling [20]. LPS from a strain of *E*. *coli* and *Pseudomonas aeruginosa* induces NOX-dependent suicidal NETosis while infection from the other Gram-negative bacteria leads to LPS interaction with platelets, platelet activation and the process of vital NETosis. However, NET induction by LPS seems to be at least in part dose-dependent and LPS from different sources may induce NETs when used at appropriate concentrations [21].

On the other side, excessive or inappropriate NETosis may trigger the production of autoantibodies and directly cause organ damage in autoimmune disorders [22, 23]. Excessive NET load due to increased production and/or defective removal may elicit interferon (IFN) I production and contribute to the induction of anti-chromatin antibodies in systemic lupus erythematosus (SLE) [24–27]. Netting neutrophils have been detected in renal biopsies of patients affected by systemic lupus or ANCA-associated vasculitis (AAV) [28]. In AAV, often associated with a necrotizing pauci-immune glomerulonephritis, NETosis is an important mechanism of endothelial damage. Autoantibodies specific for neutrophil granule-associated proteins such as MPO, lactoferrin, cathepsin G, alpha enolase are a hallmark of AAV [29, 30]. These autoantigens have all been detected in NETs induced by any stimuli. Another NET constituent relevant for AAV is hLAMP-2 (Lysosome associated membrane protein-2), a protein that shuttles between lysosomes and cell membrane.Anti-hLAMP-2 antibodies, as anti-MPO antibodies, are in fact strongly related with disease activity, being detected mainly in the active phases of AAV [31].

Other features of NET constituents that may influence their fate as autoantigens are post translational modifications (PTMs), that may render immunogenic otherwise tolerated antigens. Oxidation and citrullination, disrupting the structure of proteins and increasing the susceptibility to proteolytic degradation, allow antigen presentation and exposure of cryptic epitopes, steps that are all involved in the induction of autoimmune responses.

In NETs obtained with different stimuli, proteins differ also in PTM. PMA- and A23187-induced NETs are again similar, bearing a higher number of PTM when compared to LPS-induced and spontaneous NETs. These latter are characterized by a lower degree of oxidation: thiol alkylation, indicative of a free SH group, is in fact more frequent in spontaneous NETs. No major differences are observed in the frequency of the other PTM we studied, but individual proteins bearing PTM may be differentially enriched in NET obtained with the various stimuli: oxidized MPO and cathepsin G are abundant in PMA- and A23187-induced NET, oxidized calprotectin in A23187- and LPS-induced NET, while oxidized actin is highly represented in A23187-induced NET.

MPO and histones are representative examples of post translationally modified autoantigens contained in NETS; citrullinated histone H4 from NETs has been described as target of anti-citrullinated antibodies in RA, potentially involved in the induction of this class of autoantibodies [32,33]. Another critical histone PTM is acetylation, that contributes to chromatin unfolding [34], increases the immune stimulatory potential of NETs [35] and creates targets for autoantibodies in SLE[36]. The above described autoantigens are present in NETs produced under any stimulus, together with cathelicidin, a defense peptide that may induce maturation of plasmacytoid dendritic cells permitting antigen presentation and possibly autoreactive B cell expansion [37]. Thus, the protein composition of NETs (autoantigens together with danger signals such as cathelicidin) is consistent with their role in induction and expansion of autoantibody production.

In conclusion, proteomic analysis of NET indicates that NETs induced by different stimuli are heterogeneous in terms of protein composition and post-translational modifications. Many of the differentially expressed or differentially modified proteins are targets of autoantibodies.

This observation adds further complexity to a very complex biological phenomenon, suggesting that NET induced in different conditions by different pathways may have different biological effects.

## Materials and methods

### Isolation of neutrophils and 'in vitro' NETs induction

Neutrophils were obtained from buffy coat of healthy blood donors and isolated using discontinuous gradient centrifugation according to English *et al*. [38]. Briefly, 30 ml of buffy coat preparation were diluted to 150ml with D-PBS, stratified over a double Histopaque 1.077/1.119 gradient and centrifuged at 400g for 30 min, with low acceleration and without brake.

Granulocyte ring was recovered, cells were washed twice with cold D-PBS and counted in Burker chamber.

A total of 10–15 million neutrophils were seeded in 10mm Petri dishes in Hank’s balanced salt solution (HBSS) at 10 × 10^6^ cells/plate and treated with a) no stimulus; b) 100 nM phorbol 12-myristate 13-acetate (PMA); c) 4 μM A23187; d) 1 μg/ml LPS from *E*. *coli* (all from Sigma Aldrich) for 3 hours at 37°C.

After removing the medium, the wells were gently washed 2 × 10 min with 10ml Dulbecco-modified phosphate buffer saline (D-PBS) and incubated for 20 min at 37°C with 10 U/ml DNase I (Sigma) in HBSS + CaCl_2_ 2 mM. DNase activity was stopped by adding EDTA 5 mM (final concentration). The samples were then centrifuged 10’ at 3000g to remove intact cells and intact nuclei; the supernatants containing NET proteins were processed as described below.

In parallel, neutrophils were seeded on cover slides (400000/slide) and incubated for 3 h with medium only or with PMA, A23187, LPS or an hypertonic solution (PBS, NaCl 509 mM). Cells were washed in PBS and then fixed in PFA 4% for 10’ RT. After fixation cells were incubated with protein block (Dako) for 10’ and then with the proper primary antibody: anti- MPO (Dako, 1:300) to visualise NETs, or anti-cleaved Caspase 3 (Cell Signalling Technology, 1:250) to visualise apoptotic cells. After a PBS wash, cells were incubated with goat anti-rabbit Alexa488 secondary antibody (Invitrogen, 1:300) for 30’. To visualise the nucleus, cells were incubated for 10’ with DAPI. After a wash in PBS, cover slides were mounted with Mowiol on a slide and analysed with a fluorescence microscope.

Counts of stained cells were performed in five 40x microscopic fields per section.

The study was approved by the local Ethics Committee, Comitato Etico per la Sperimentazione Clinica Area Vasta Nord Ovest (CEAVNO)- protocol 3661/2012. No informed consent was obtained because the data were analyzed anonymously.

### Sample preparation for mass spectrometry and mass spectrometer setup

The supernatant containing NET proteins was precipitated with acetone and NET pellets were re-suspended in 25 μl of lysis buffer (6M GdmCl, 10mM TCEP, 40mM CAA, and 100 mM Tris pH8.5). The samples were reduced, alkylated and lastly digested in a single step and then loaded into StageTip. Peptides were analyzed by nano-UHPLC-MS/MS using an Ultimate 3000 RSLC with EASY spray column (75 μm x 500 mm, 2 μm particle size, Thermo Scientific) and with a 180 minute non-linear gradient of 5–45% solution B (80% CAN and 20% H_2_O, 5% DMSO, 0.1% FA) at a flow rate of 250 nl/min. Eluting peptides were analyzed using an Orbitrap Fusion Tribrid mass spectrometer (Thermo Scientific Instruments, Bremen, Germany). Orbitrap detection was used for both MS1 and MS2 measurements at resolving powers of 120 K and 30 K (at m/z 200), respectively. Data dependent MS/MS analysis was performed in top speed mode with a 2 sec. cycletime, during which precursors detected within the range of m/z 375−1500 were selected for activation in order of abundance. Quadrupole isolation with a 1.4 m/z isolation window was used, and dynamic exclusion was enabled for 45 s. Automatic gain control targets were 2.5 × 10^5^ for MS1 and 5 × 10^4^ for MS2, with 50 and 60 ms maximum injection times, respectively. The signal intensity threshold for MS2 was 1 × 10^4^. HCD was performed using 30% normalized collision energy. One microscan was used for both MS1 and MS2 events. For all the MS1 scans the option ETD internal Calibration was selected.

MaxQuant software, version 1.5.5.30, was used to process the raw data, setting a false discovery rate (FDR) of 0.01 for the identification of proteins, peptides and PSM (peptide-spectrum match), moreover a minimum length of 6 amino acids for peptide identification was required. Andromeda engine, incorporated into MaxQuant software, was used to search MS/MS spectra against Uniprot human database (release UP000005640_9606 February 2016).

Two different elaborations were made to identify the PTMs in order to limit the false positives. In the first processing, variable modifications were Acetylation (Protein N-Term), Oxidation (M), Deamidation (NQR) and Carbamido-methylation (C). Finally, in order to overcome the common limitations of search engine based PTM analysis, we used the unbiased PTM Dependent Peptide (DP) search option, taking advantage of high mass accuracy data collected in high resolution mode with an internal calibration (MS1 error < 1 ppm). **).** The DP peptide table was extracted from allPeptides.txt by Plugin Dependent Peptides of Perseus (https://github.com/jdrudolph/PluginDependentPeptides) and filtered by score >80. The intensity values were extracted and statistically evaluated using the different Site Table, DP table or Protein Groups table. Algorithm MaxLFQbwas chosen for the protein quantification with the activated option ‘match between runs’to reduce the number of the missing proteins.

The mass spectrometry proteomics data have been deposited to the ProteomeXchange Consortium via the PRIDE [39] partner repository under the Project Name “NET induced by different stimuli: a comparative proteomic analysis” and the dataset identifier PXD012951.

### Statistics and bioinformatic analysis

After normalization, data obtained from mass spectrometry were analyzed using unsupervised hierarchical clustering analyses, i.e. Multidimensional Scaling (MDS) and Spearman's Correlation, to identify outlier and samples dissimilarity. Differences in proteins and peptides expression between spontaneous and stimuli-induced NET samples were detected using a non-parametric U-Mann Whitney test and p-values were adjusted using the Benjamini-Hochberg method. Results were considered significant with two-fold change and adjusted for P-value ≤ 0.05. Volcano plot was used to visualize the statistical differences, in which case the cutoff lines were established using the function y = c/(x—x0). Non-linear support vector machine (SVM) and partial least squares discriminant analysis (PLS-DA) were utilized to identify maximal discrimination among groups. In SVM, a cross-validation approach a 4-fold increment limit was applied to estimate the accuracy of classification and make a ranked proteins list. The results of these analyses were summarized by mean of heat map graph.

Cytoscape software was utilized for gene ontology and pathway analysis. The list of identified proteins was validated according to their gene ontology (GO) annotation extracted from the UniProt, Reactome, KEGG and ClueGO database. The results of GO analysis were shown by mean of heat map. All statistical analysis were performed using software package R last version available at the time of experiments.

## Supporting information

S1 Fig MPO/DAPI IF on neutrophils untreated (A-C) or treated with PMA (D-F), A23187 (G-I) or LPS (J-L). A graph in M summarizes the results.
(TIF)

S2 Fig Cleaved Caspase 3/DAPI IF on neutrophils untreated (A, B), or treated with PMA (C-E), A23187 (F-H), LPS (I-K) or hypertonic solution (J-N) treated. A graph in O summarizes the results.
(TIF)

S1 Table List of all proteins identified by mass spectrometry. The symbol “+” identify the proteins present in each experimental condition (Fig 1A).
(DOCX)

S2 Table List of all post-translational modifications identified by mass spectrometry using dependent peptide (DP) and variable modification (VM) methods. The symbol “+” identify the post-translational modifications present in each experimental condition (Fig 7).
(DOCX)
