# Supplementary figures and images for: Neutrophil extracellular traps (NET) induced by different stimuli: A comparative proteomic analysis
Source: PLoS One. 2019 Jul 8;14(7):e0218946. doi: 10.1371/journal.pone.0218946 (PMC6613696; doi:10.1371/journal.pone.0218946)

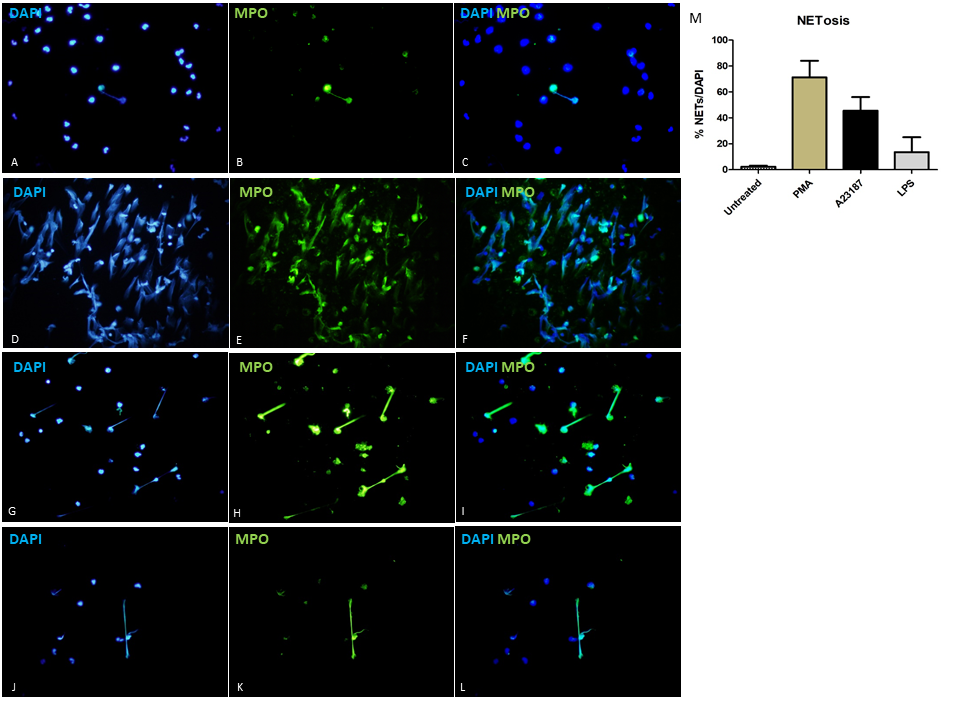

Supplement: S1 Fig — MPO/DAPI IF on neutrophils untreated (A-C) or treated with PMA (D-F), A23187 (G-I) or LPS (J-L). A graph in M summarizes the results. (TIF) [file pone.0218946.s001.tif]

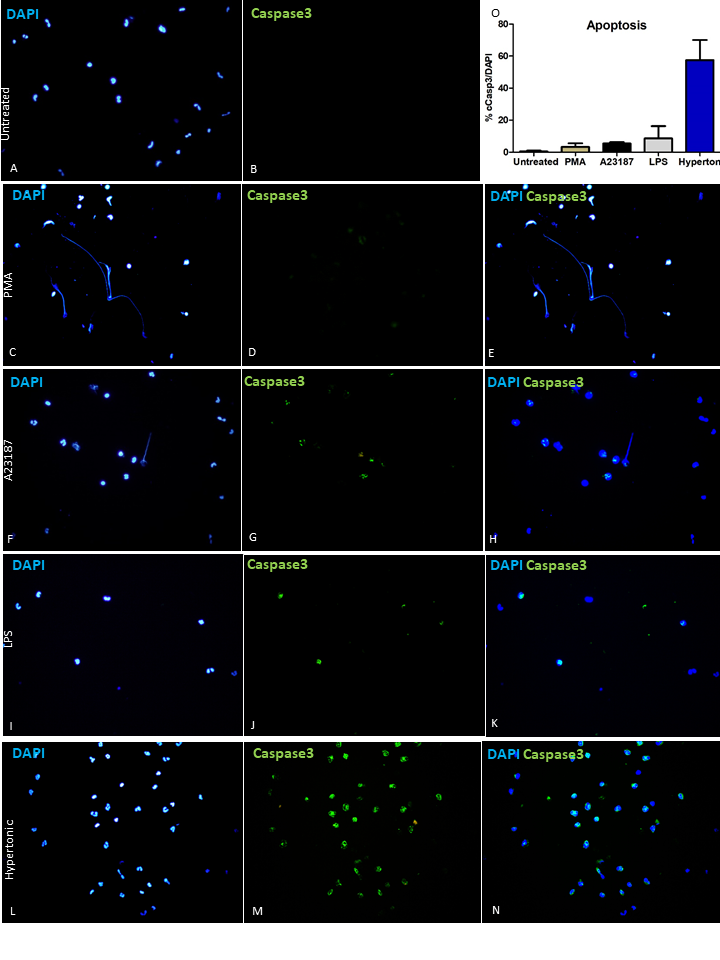

Supplement: S2 Fig — Cleaved Caspase 3/DAPI IF on neutrophils untreated (A, B), or treated with PMA (C-E), A23187 (F-H), LPS (I-K) or hypertonic solution (J-N) treated. A graph in O summarizes the results. (TIF) [file pone.0218946.s002.tif]
