# Supplementary material for: Neutrophil extracellular traps (NET) induced by different stimuli: A comparative proteomic analysis
Source: PLoS One. 2019 Jul 8;14(7):e0218946. doi: 10.1371/journal.pone.0218946 (PMC6613696; doi:10.1371/journal.pone.0218946)
Supplement: S1 Table — The symbol “+” identify the proteins present in each experimental condition (Fig 1A). (DOCX) [file pone.0218946.s003.docx]

**Supplementary Table 1**. List of all proteins identified by mass spectrometry. The symbol “+” identify the proteins present in each experimental condition (Figure 1a).

| Protein IDs | Protein names | Gene names | Number of proteins | Peptides | Unique peptides | Sequence coverage [%] | Unique sequence coverage [%] | Score | MS/MS count | Spontaneous | PMA | LPS | A23187 |
| --- | --- | --- | --- | --- | --- | --- | --- | --- | --- | --- | --- | --- | --- |
| C9JC03 | EH domain-containing protein 1 | EHD1 | 8 | 3 | 3 | 10.2 | 10.2 | 32 | 32 | + | + | + | + |
| A0A075B754 | ATPase family AAA domain-containing protein 5 | ATAD5 | 3 | 1 | 1 | 1.1 | 1.1 | 2 | 1 |  |  |  | + |
| A0A140TA77 | Protein-tyrosine-phosphatase | PTPRC | 12 | 2 | 2 | 6.1 | 6.1 | 16 | 8 | + | + | + | + |
| A0A087WTK0 | Protein-tyrosine-phosphatase | PTPRJ | 4 | 2 | 2 | 2.3 | 2.3 | 28 | 17 | + | + | + | + |
| A0A087WUI2 | Heterogeneous nuclear ribonucleoproteins A2/B1 | HNRNPA2B1 | 3 | 1 | 1 | 6.1 | 6.1 | 14 | 2 | + | + |  |  |
| A0A087WUZ3 | Spectrin beta chain, non-erythrocytic 1 | SPTBN1 | 5 | 4 | 4 | 2.3 | 2.3 | 28 | 12 | + | + | + | + |
| A0A087WVQ6 | Clathrin heavy chain | CLTC | 9 | 9 | 9 | 8.4 | 8.4 | 131 | 17 | + | + |  |  |
| A0A087WVW7 | Methyl-CpG-binding protein 2 | MECP2 | 7 | 1 | 1 | 12.5 | 12.5 | 63 | 1 | + |  |  |  |
| A0A087X2E3 | Synapsin-2 | SYN2 | 5 | 2 | 2 | 7.3 | 7.3 | 48 | 6 | + | + |  |  |
| K7EPV9 | Tropomyosin alpha-3 chain | TPM4 | 18 | 1 | 1 | 8.3 | 8.3 | 12 | 14 | + | + | + | + |
| F8WE71 | Serine/threonine-protein phosphatase | PPP1CB | 16 | 1 | 1 | 26.6 | 26.6 | 19 | 2 | + | + |  |  |
| M0R213 | Beta-soluble NSF attachment protein | NAPA | 7 | 1 | 1 | 13.3 | 13.3 | 67 | 2 | + | + |  |  |
| A0A087X0P0 | Kinesin-like protein | CENPE | 4 | 1 | 1 | 0.4 | 0.4 | 62 | 7 |  | + | + | + |
| H0Y8X1 | Succinate dehydrogenase [ubiquinone] flavoprotein subunit, mitochondrial | SDHA | 6 | 1 | 1 | 11.1 | 11.1 | 13 | 2 | + | + |  |  |
| A0A087X1W2 | Protein arginine N-methyltransferase 1 | PRMT1 | 7 | 1 | 1 | 3.9 | 3.9 | 70 | 1 | + |  |  |  |
| E9PN91 | Elongation factor 1-delta | EEF1D | 15 | 1 | 1 | 11.3 | 11.3 | 94 | 5 | + | + | + | + |
| A0A0C4DGD1 | Calpastatin | CAST | 23 | 1 | 1 | 8.4 | 8.4 | 9 | 0 | + |  |  |  |
| A0A0A0MRQ5 | Peroxiredoxin-2 | PRDX1 | 4 | 1 | 1 | 11.3 | 11.3 | 63 | 1 | + |  |  |  |
| A0A0A0MRV0 | Ribosome-binding protein 1 | RRBP1 | 7 | 4 | 4 | 14 | 14 | 25 | 4 | + | + | + | + |
| A0A0A0MS51 | Gelsolin | GSN | 8 | 3 | 3 | 3.5 | 3.5 | 19 | 13 | + | + | + | + |
| M0R116 | Sodium/potassium-transporting ATPase subunit alpha-3 | ATP1A3 | 14 | 13 | 8 | 19.8 | 11.6 | 162 | 20 | + | + |  |  |
| H7C4R7 | Deoxyribonuclease | DNASE1L3 | 4 | 1 | 1 | 5.8 | 5.8 | 10 | 79 | + | + | + | + |
| A0A0A0MTH9 | TATA-binding protein-associated factor 172 | BTAF1 | 3 | 1 | 1 | 0.5 | 0.5 | 2 | 0 |  | + |  | + |
| A0A0A0MTI9 | Actin-related protein 3C | ACTR3C | 9 | 1 | 1 | 5.8 | 5.8 | 78 | 1 |  | + |  |  |
| A0A0J9YXP8 | Glucose-6-phosphate isomerase | GPI | 7 | 1 | 1 | 4.1 | 4.1 | 62 | 2 |  |  |  | + |
| A0A0B4J1R6 | Transketolase | TKT | 3 | 1 | 1 | 1.5 | 1.5 | 64 | 2 | + | + |  |  |
| F8VV32 | Lysozyme | LYZ | 3 | 1 | 1 | 11.5 | 11.5 | 83 | 4 |  | + | + | + |
| A0A0C4DG56 | Superoxide dismutase | SOD2 | 7 | 1 | 1 | 8.6 | 8.6 | 90 | 2 | + | + |  |  |
| A0A0C4DFZ1 | Syntaxin-1A | STX1A | 5 | 1 | 1 | 5 | 5 | 79 | 1 |  | + |  |  |
| H0YIC4 | Citrate synthase | CS | 4 | 1 | 1 | 8.9 | 8.9 | 64 | 1 |  | + |  |  |
| A0A0C4DGJ9 | Granzyme H | GZMH | 4 | 1 | 1 | 8.7 | 8.7 | 76 | 10 | + | + | + | + |
| C9J7N5 | Serpin I2 | SERPINI2 | 3 | 3 | 3 | 11.7 | 11.7 | 28 | 20 |  | + | + | + |
| A0A0D9SF54 | Spectrin alpha chain, non-erythrocytic 1 | SPTAN1 | 6 | 3 | 3 | 2.2 | 2.2 | 57 | 3 | + | + |  |  |
| A0A0D9SFB1 | Dynamin-1 | DNM1 | 16 | 2 | 2 | 3.6 | 3.6 | 25 | 3 | + | + |  |  |
| E9PFG7 | 2-oxoglutarate dehydrogenase, mitochondrial | OGDH | 6 | 2 | 2 | 3.7 | 3.7 | 18 | 2 |  | + |  |  |
| A0A1B0GW76 | Syntaxin-binding protein 1 | STXBP1 | 7 | 2 | 2 | 7.5 | 7.5 | 31 | 5 | + | + |  |  |
| C9JW66 | Synaptojanin-1 | SYNJ1 | 9 | 1 | 1 | 2.3 | 2.3 | 94 | 1 |  | + |  |  |
| A0A0G2JIW1 | Heat shock 70 kDa protein 1B | HSPA1B | 5 | 4 | 3 | 8.6 | 6.1 | 27 | 6 |  | + | + | + |
| A0A0G2JNJ8 | Mucin-6 | MUC6 | 3 | 1 | 1 | 0.3 | 0.3 | 60 | 3 |  | + |  | + |
| A0A0G2JQK5 | Nucleoredoxin | NXN | 2 | 1 | 1 | 10 | 10 | 62 | 1 |  | + |  |  |
| A0A0G2JQM1 | AP-2 complex subunit alpha-2 | AP2A2 | 7 | 1 | 1 | 2.8 | 2.8 | 10 | 2 | + | + |  |  |
| F5H823 | Ras-related protein Rap-1b | RAP1B | 10 | 1 | 1 | 11.7 | 11.7 | 97 | 2 | + | + |  |  |
| A0A0J9YXZ5 | Ras GTPase-activating-like protein IQGAP1 | IQGAP1 | 2 | 1 | 1 | 1.5 | 1.5 | 13 | 1 |  |  |  | + |
| Q9HCH5-13 |  | SYTL2 | 2 | 1 | 1 | 1.1 | 1.1 | 63 | 90 | + | + | + | + |
| H3BTW5 | C-myc promoter-binding protein | DENND4A | 4 | 1 | 1 | 0.6 | 0.6 | 38 | 5 | + | + | + | + |
| A0A1B0GTF3 | Heat shock 70 kDa protein 12A | HSPA12A | 2 | 1 | 1 | 2.5 | 2.5 | 60 | 1 |  | + |  |  |
| A0A1B0GVA3 |  | ASAH1 | 4 | 1 | 1 | 7.3 | 7.3 | 75 | 0 |  | + |  |  |
| A0A1B0GUW7 |  | SMIM27 | 2 | 1 | 1 | 14.5 | 14.5 | 2 | 2 |  |  | + | + |
| A0A1B0GVI3 | Keratin, type I cytoskeletal 10 | KRT10 | 3 | 1 | 1 | 12.1 | 12.1 | 64 | 1 |  | + |  |  |
| E5RI95 | Neuron-specific calcium-binding protein hippocalcin | NCALD | 13 | 1 | 1 | 10.5 | 10.5 | 8 | 2 | + | + |  |  |
| A0A1C7CYX9 | Dihydropyrimidinase-related protein 2 | DPYSL2 | 7 | 7 | 7 | 17.6 | 17.6 | 99 | 6 | + | + |  |  |
| C9J080 | Beta-adducin | ADD2 | 11 | 1 | 1 | 8 | 8 | 11 | 1 |  | + |  |  |
| A1A4F0 | Putative uncharacterized protein PQLC2L | PQLC2L | 1 | 1 | 1 | 11.1 | 11.1 | 63 | 2 |  | + |  | + |
| A2A274 | Aconitate hydratase, mitochondrial | ACO2 | 2 | 2 | 2 | 5.3 | 5.3 | 26 | 4 | + | + |  |  |
| A2A2D0 | Stathmin | STMN1 | 3 | 1 | 1 | 14.1 | 14.1 | 6 | 2 | + | + |  |  |
| A2A3R5 | 40S ribosomal protein S6 | RPS6 | 2 | 1 | 1 | 5.5 | 5.5 | 92 | 17 | + | + | + | + |
| A6NC48 | ADP-ribosyl cyclase/cyclic ADP-ribose hydrolase 2 | BST1 | 3 | 1 | 1 | 5.7 | 5.7 | 29 | 3 |  | + | + | + |
| A6NL93 | Non-histone chromosomal protein HMG-14 | HMGN1 | 3 | 1 | 1 | 14.4 | 14.4 | 67 | 2 | + |  |  |  |
| B7ZBA8 | Coiled-coil domain-containing protein 154 | CCDC154 | 3 | 1 | 1 | 2.1 | 2.1 | 2 | 4 |  | + | + |  |
| A8MXH2 | Nucleosome assembly protein 1-like 4 | NAP1L4 | 6 | 1 | 1 | 7.1 | 7.1 | 61 | 1 |  | + |  |  |
| E9PMR5 | Myelin basic protein | MBP | 16 | 1 | 1 | 12.2 | 12.2 | 78 | 3 | + | + |  |  |
| B0YJC4 | Vimentin | VIM | 5 | 6 | 6 | 16.5 | 16.5 | 45 | 25 | + | + | + | + |
| B1AHC9 | X-ray repair cross-complementing protein 6 | XRCC6 | 3 | 1 | 1 | 3.9 | 3.9 | 10 | 1 | + |  |  |  |
| Q5T7C4 | Putative high mobility group protein B1-like 1 | HMGB1 | 3 | 2 | 2 | 9.5 | 9.5 | 15 | 17 | + | + | + | + |
| H0YAP7 | Elongator complex protein 3 | ELP3 | 6 | 1 | 1 | 11.4 | 11.4 | 65 | 1 |  |  |  | + |
| B4DM24 | CaM kinase-like vesicle-associated protein | CAMKV | 5 | 1 | 1 | 3.5 | 3.5 | 7 | 1 | + |  |  |  |
| B4DUR8 | T-complex protein 1 subunit gamma | CCT3 | 3 | 1 | 1 | 2.8 | 2.8 | 63 | 11 |  | + | + | + |
| J3QS39 | Ubiquitin-60S ribosomal protein L40 | UBB | 23 | 2 | 2 | 26.9 | 26.9 | 74 | 6 | + | + | + | + |
| B5MCN7 | Nuclear receptor coactivator 1 | NCOA1 | 4 | 1 | 1 | 0.6 | 0.6 | 62 | 0 |  | + |  |  |
| B5MCP9 | 40S ribosomal protein S7 | RPS7 | 2 | 1 | 1 | 11.8 | 11.8 | 43 | 2 | + | + |  |  |
| H0YFC6 | GTP-binding nuclear protein Ran | RAN | 5 | 2 | 2 | 18.4 | 18.4 | 14 | 3 |  | + |  | + |
| H0Y9U7 | Long-chain-fatty-acid--CoA ligase 1 | ACSL1 | 6 | 1 | 1 | 2.4 | 2.4 | 2 | 1 |  |  | + |  |
| F6UXX1 | Heterogeneous nuclear ribonucleoprotein Q | SYNCRIP | 7 | 1 | 1 | 7 | 7 | 99 | 2 | + | + |  |  |
| F8VZU9 | Myosin light polypeptide 6 | MYL6 | 13 | 2 | 2 | 24.3 | 24.3 | 25 | 3 |  |  |  | + |
| B8ZZ51 | Malate dehydrogenase, cytoplasmic | MDH1 | 5 | 1 | 1 | 7.1 | 7.1 | 66 | 2 | + | + |  |  |
| B8ZZQ6 | Prothymosin alpha | PTMA | 6 | 1 | 1 | 12.1 | 12.1 | 11 | 2 | + | + |  |  |
| C9J8H1 | V-type proton ATPase subunit E 1 | ATP6V1E1 | 3 | 1 | 1 | 9.9 | 9.9 | 323 | 2 | + | + |  |  |
| C9J9W2 | LIM and SH3 domain protein 1 | LASP1 | 4 | 1 | 1 | 7.8 | 7.8 | 62 | 1 | + |  |  |  |
| C9JB30 | Microtubule-associated protein RP/EB family member 3 | MAPRE3 | 3 | 1 | 1 | 5.5 | 5.5 | 96 | 1 | + |  |  |  |
| C9JB90 | Ras-related protein Rab-6A | RAB6B | 9 | 1 | 1 | 22.4 | 22.4 | 67 | 2 | + | + |  |  |
| C9JI87 | Voltage-dependent anion-selective channel protein 1 | VDAC1 | 2 | 2 | 2 | 13.1 | 13.1 | 14 | 3 | + | + |  |  |
| C9JNW0 |  | SH3BP5 | 3 | 1 | 1 | 35.3 | 35.3 | 63 | 1 |  |  | + |  |
| G5E9W8 | Glycogenin-1 | GYG1 | 7 | 4 | 4 | 25.9 | 25.9 | 32 | 20 | + | + | + | + |
| F8VR50 | Actin-related protein 2/3 complex subunit 3 | ARPC3 | 3 | 1 | 1 | 15.5 | 15.5 | 98 | 1 | + |  |  |  |
| P00766-1 |  | NA | 1 | 1 | 1 | 13.3 | 13.3 | 70 | 1 |  | + |  |  |
| F8VP67 | Keratin, type II cytoskeletal 8 | KRT8 | 7 | 1 | 1 | 10.6 | 10.6 | 6 | 2 | + | + |  |  |
| P00761 |  | NA | 1 | 4 | 4 | 25.1 | 25.1 | 262 | 214 | + | + | + | + |
| P00766 |  | NA | 1 | 6 | 6 | 36.3 | 36.3 | 323 | 63 | + | + | + | + |
| P69905 | Hemoglobin subunit alpha | HBA1 | 1 | 3 | 1 | 28.2 | 10.6 | 10 | 10 | + | + | + | + |
| P02769 |  | NA | 12 | 31 | 31 | 48.6 | 48.6 | 323 | 438 | + | + | + | + |
| P04264 | Keratin, type II cytoskeletal 1 | KRT1 | 14 | 6 | 6 | 11.2 | 11.2 | 39 | 10 | + | + | + | + |
| P12763 |  | NA | 1 | 2 | 2 | 10.9 | 10.9 | 18 | 3 | + | + |  |  |
| P34955 |  | NA | 1 | 1 | 1 | 3.6 | 3.6 | 18 | 2 | + | + |  |  |
| P35527 | Keratin, type I cytoskeletal 9 | KRT9 | 3 | 3 | 3 | 5.9 | 5.9 | 22 | 7 | + | + |  | + |
| Q3SX09 |  | NA | 1 | 2 | 1 | 14.4 | 9.5 | 28 | 2 | + | + |  |  |
| Q3SZV7 |  | NA | 1 | 1 | 1 | 5 | 5 | 12 | 2 |  |  |  | + |
| Q86YZ3 | Hornerin | HRNR | 2 | 1 | 1 | 1.7 | 1.7 | 9 | 7 |  | + | + | + |
| D3DSM0 | Integrin beta | ITGB2 | 2 | 1 | 1 | 1.8 | 1.8 | 69 | 4 | + | + |  | + |
| D6REK8 | Glutamate receptor 2 | GRIA2 | 7 | 1 | 1 | 13.5 | 13.5 | 86 | 1 |  | + |  |  |
| D6RD66 | WD repeat-containing protein 1 | WDR1 | 2 | 1 | 1 | 8.6 | 8.6 | 69 | 1 |  |  |  | + |
| D6REY1 | Chitotriosidase-1 | CHIT1 | 5 | 1 | 1 | 5.8 | 5.8 | 71 | 1 |  |  |  | + |
| D6RFF0 | La-related protein 7 | LARP7 | 4 | 1 | 1 | 7 | 7 | 69 | 9 | + | + | + |  |
| D6RG09 |  | SENP6 | 1 | 1 | 1 | 3.2 | 3.2 | 2 | 1 | + |  |  |  |
| D6RGY2 | Calnexin | CANX | 6 | 3 | 3 | 11.7 | 11.7 | 188 | 27 | + | + | + | + |
| E5RG14 | Calbindin | CALB1 | 4 | 1 | 1 | 20.6 | 20.6 | 13 | 1 |  | + |  |  |
| E5RI98 | Nucleophosmin | NPM1 | 4 | 1 | 1 | 18.9 | 18.9 | 21 | 3 | + | + |  |  |
| E9PLD0 | Ras-related protein Rab-1A | RAB1B | 6 | 1 | 1 | 9.5 | 9.5 | 75 | 1 |  | + |  |  |
| E7ENU9 | Macrophage-capping protein | CAPG | 3 | 1 | 1 | 4.7 | 4.7 | 11 | 1 | + |  |  |  |
| E7EP32 | Guanine nucleotide-binding protein G(I)/G(S)/G(T) subunit beta-2 | GNB2 | 10 | 2 | 1 | 9.8 | 6.1 | 15 | 2 | + |  |  |  |
| E7EQB2 | Lactotransferrin | LTF | 7 | 39 | 39 | 54.6 | 54.6 | 323 | 439 | + | + | + | + |
| E7ERH2 | S-phase kinase-associated protein 1 | SKP1 | 4 | 1 | 1 | 13.4 | 13.4 | 13 | 1 |  | + |  |  |
| E7ETG2 |  | ERN2 | 1 | 1 | 1 | 2.4 | 2.4 | 64 | 13 |  |  | + | + |
| E9PCB6 | Neurolysin, mitochondrial | NLN | 2 | 1 | 1 | 2.3 | 2.3 | 69 | 1 |  |  |  | + |
| H0YBG7 | Heterogeneous nuclear ribonucleoprotein F | HNRNPH1 | 7 | 1 | 1 | 9.2 | 9.2 | 89 | 1 | + |  |  |  |
| E9PEW8 |  | HBD | 2 | 6 | 1 | 53.8 | 7.7 | 63 | 5 |  |  | + | + |
| E9PHT9 | Annexin | ANXA5 | 2 | 1 | 1 | 9.8 | 9.8 | 19 | 1 |  |  |  | + |
| G3V1A4 | Cofilin-1 | CFL1 | 8 | 3 | 3 | 28.2 | 28.2 | 25 | 4 | + | + |  |  |
| E9PK47 | Alpha-1,4 glucan phosphorylase | PYGL | 3 | 1 | 1 | 1.7 | 1.7 | 14 | 2 | + | + |  |  |
| E9PKZ0 | 60S ribosomal protein L8 | RPL8 | 5 | 3 | 3 | 12.7 | 12.7 | 25 | 6 | + | + | + | + |
| F8VSA6 | NEDD8 | NEDD8 | 5 | 1 | 1 | 22 | 22 | 76 | 1 | + |  |  |  |
| E9PLB5 | Protein phosphatase 1 regulatory subunit 36 | PPP1R36 | 3 | 1 | 1 | 33.3 | 33.3 | 76 | 4 | + | + | + | + |
| H3BNG3 | 40S ribosomal protein S2 | RPS2 | 8 | 2 | 2 | 61.4 | 61.4 | 22 | 3 | + | + |  |  |
| E9PN76 | RING finger protein 214 | RNF214 | 3 | 1 | 1 | 1.9 | 1.9 | 2 | 0 |  |  | + |  |
| H0YCA4 | Low-density lipoprotein receptor-related protein 8 | LRP8 | 6 | 1 | 1 | 9.1 | 9.1 | 69 | 24 | + | + | + | + |
| F5H0C5 | Prohibitin-2 | PHB2 | 5 | 1 | 1 | 11.6 | 11.6 | 68 | 2 | + | + |  |  |
| J3QQV3 | Centrosomal protein of 112 kDa | CEP112 | 3 | 1 | 1 | 2.1 | 2.1 | 7 | 1 |  |  |  | + |
| F5GYK7 | Glycerol-3-phosphate dehydrogenase | GPD2 | 3 | 1 | 1 | 3.2 | 3.2 | 95 | 1 |  | + |  |  |
| F5H0C7 | ADP-ribosylation factor 1 | ARF3 | 8 | 2 | 2 | 34.3 | 34.3 | 28 | 4 | + | + |  |  |
| F5H1M8 | Protein-L-isoaspartate O-methyltransferase domain-containing protein 1 | PCMTD1 | 3 | 1 | 1 | 3.6 | 3.6 | 2 | 2 |  | + |  | + |
| F5H2B9 | Uveal autoantigen with coiled-coil domains and ankyrin repeats | UACA | 4 | 1 | 1 | 0.9 | 0.9 | 2 | 0 |  |  |  | + |
| F5H2R5 | Rho GDP-dissociation inhibitor 2 | ARHGDIB | 5 | 2 | 2 | 34.1 | 34.1 | 46 | 43 | + | + | + | + |
| F5H5G1 | Limbic system-associated membrane protein | LSAMP | 3 | 1 | 1 | 5.6 | 5.6 | 19 | 2 | + | + |  |  |
| F5H6L7 | Sodium/calcium exchanger 2 | SLC8A2 | 2 | 2 | 2 | 4.7 | 4.7 | 21 | 2 |  | + |  |  |
| F5H6T1 | Actin-related protein 2 | ACTR2 | 3 | 1 | 1 | 2.1 | 2.1 | 67 | 59 | + | + | + | + |
| F6U236 |  | PACSIN1 | 1 | 1 | 1 | 2.7 | 2.7 | 61 | 4 |  | + |  |  |
| F8VU11 | Pre-mRNA-processing factor 40 homolog B | PRPF40B | 4 | 1 | 1 | 1.7 | 1.7 | 70 | 1 |  |  | + |  |
| G3V1N2 |  | HBA2 | 1 | 4 | 1 | 33.6 | 7.3 | 6 | 3 |  | + | + | + |
| H0YJF9 | Dihydrolipoyllysine-residue succinyltransferase component of 2-oxoglutarate dehydrogenase complex, mitochondrial | DLST | 5 | 1 | 1 | 18.9 | 18.9 | 7 | 1 |  | + |  |  |
| H0YDX6 | CD44 antigen | CD44 | 24 | 1 | 1 | 6.6 | 6.6 | 67 | 3 | + |  | + | + |
| H0Y2Y8 | Zyxin | ZYX | 5 | 3 | 3 | 8.7 | 8.7 | 34 | 5 | + | + |  |  |
| R4GMN8 | Phosphatidylinositol 3,4,5-trisphosphate 5-phosphatase 1 | INPP5D | 5 | 1 | 1 | 30.2 | 30.2 | 63 | 2 |  | + |  | + |
| P0DP25 |  | CALM2 | 14 | 6 | 6 | 47.7 | 47.7 | 53 | 30 | + | + | + | + |
| H0Y8X7 | Coatomer subunit gamma-1 | COPG1 | 2 | 1 | 1 | 8.5 | 8.5 | 60 | 1 |  |  |  | + |
| H0YC45 | V-type proton ATPase subunit B, brain isoform | ATP6V1B2 | 2 | 1 | 1 | 42.9 | 42.9 | 21 | 2 | + | + |  |  |
| H0YE38 |  | FHAD1 | 1 | 1 | 1 | 1.3 | 1.3 | 2 | 0 | + |  |  |  |
| H0YLP6 | 60S ribosomal protein L28 | RPL28 | 8 | 1 | 1 | 12.4 | 12.4 | 67 | 4 | + | + |  |  |
| H3BRN4 | 4-aminobutyrate aminotransferase, mitochondrial | ABAT | 3 | 2 | 2 | 5.2 | 5.2 | 21 | 3 | + | + |  |  |
| H3BQN4 | Fructose-bisphosphate aldolase | ALDOA | 10 | 4 | 4 | 20.2 | 20.2 | 42 | 14 | + | + | + | + |
| H3BT58 | Coactosin-like protein | COTL1 | 2 | 1 | 1 | 21.9 | 21.9 | 20 | 2 | + | + |  |  |
| H3BTN5 | Pyruvate kinase | PKM | 15 | 8 | 8 | 25.2 | 25.2 | 82 | 16 | + | + |  |  |
| H7BZX1 | Sorbin and SH3 domain-containing protein 2 | SORBS2 | 16 | 1 | 1 | 3.2 | 3.2 | 85 | 9 | + | + | + | + |
| H7C1J4 | UHRF1-binding protein 1 | UHRF1BP1 | 2 | 1 | 1 | 0.7 | 0.7 | 6 | 0 | + | + |  | + |
| H7C272 |  | HUS1 | 1 | 1 | 1 | 9.5 | 9.5 | 2 | 0 |  |  | + |  |
| H7C3F9 | Actin-related protein 2/3 complex subunit 2 | ARPC2 | 2 | 1 | 1 | 12.1 | 12.1 | 33 | 2 | + | + |  |  |
| I3L0N3 | Vesicle-fusing ATPase | NSF | 3 | 4 | 4 | 8.3 | 8.3 | 42 | 5 | + | + |  |  |
| I3L3Q7 | Complement component 1 Q subcomponent-binding protein, mitochondrial | C1QBP | 3 | 1 | 1 | 14.7 | 14.7 | 30 | 2 | + | + |  |  |
| J3KNB4 | Cathelicidin antimicrobial peptide | CAMP | 2 | 2 | 2 | 9.8 | 9.8 | 15 | 9 | + | + | + | + |
| J3KQA0 | Synaptotagmin-1 | SYT1 | 3 | 4 | 4 | 13.8 | 13.8 | 25 | 5 | + | + |  |  |
| K7EJB9 | Calreticulin | CALR | 3 | 2 | 2 | 15.8 | 15.8 | 34 | 14 | + | + | + | + |
| K7EK07 | Histone H3 | H3F3B | 11 | 3 | 3 | 34.8 | 34.8 | 27 | 9 | + | + | + | + |
| K7EP16 | Eukaryotic translation initiation factor 3 subunit G | EIF3G | 5 | 1 | 1 | 5.9 | 5.9 | 7 | 0 |  | + |  |  |
| M0QX52 | Microtubule-associated protein RP/EB family member 2 | MAPRE2 | 8 | 1 | 1 | 9.4 | 9.4 | 96 | 2 | + | + |  |  |
| K7EMN2 | 6-phosphogluconate dehydrogenase, decarboxylating | PGD | 5 | 4 | 4 | 30.6 | 30.6 | 24 | 5 | + | + | + | + |
| K7EQ37 | Protein unc-13 homolog D | UNC13D | 5 | 1 | 1 | 3.7 | 3.7 | 61 | 0 |  |  |  | + |
| K7EQ61 | HAUS augmin-like complex subunit 5 | HAUS5 | 2 | 1 | 1 | 6.5 | 6.5 | 2 | 0 |  |  | + |  |
| K7ERC4 | 2,3-cyclic-nucleotide 3-phosphodiesterase | CNP | 3 | 1 | 1 | 7.9 | 7.9 | 62 | 2 | + | + |  |  |
| M0QZ24 | Lysosomal alpha-mannosidase | MAN2B1 | 3 | 1 | 1 | 8.1 | 8.1 | 11 | 1 |  |  |  | + |
| O00254-2 |  | F2RL2 | 1 | 1 | 1 | 2.8 | 2.8 | 62 | 19 | + | + | + | + |
| O00299 | Chloride intracellular channel protein 1 | CLIC1 | 1 | 1 | 1 | 5 | 5 | 64 | 1 |  | + |  |  |
| O14862 | Interferon-inducible protein AIM2 | AIM2 | 1 | 1 | 1 | 2.3 | 2.3 | 61 | 9 | + | + | + | + |
| O15427 | Monocarboxylate transporter 4 | SLC16A3 | 1 | 1 | 1 | 2.8 | 2.8 | 65 | 0 | + |  |  | + |
| O43707 | Alpha-actinin-4 | ACTN4 | 10 | 7 | 2 | 7.7 | 2.4 | 12 | 1 |  | + |  | + |
| O43823 | A-kinase anchor protein 8 | AKAP8 | 1 | 1 | 1 | 1.9 | 1.9 | 2 | 0 |  |  |  | + |
| U3KQK0 |  | HIST1H2BN | 17 | 5 | 5 | 30.1 | 30.1 | 323 | 587 | + | + | + | + |
| O75533 | Splicing factor 3B subunit 1 | SF3B1 | 1 | 2 | 2 | 2.1 | 2.1 | 17 | 4 | + | + |  | + |
| O75746-2 | Calcium-binding mitochondrial carrier protein Aralar1 | SLC25A12 | 2 | 1 | 1 | 4.2 | 4.2 | 66 | 1 | + |  |  |  |
| O75923-15 | Dysferlin | DYSF | 15 | 2 | 2 | 1 | 1 | 14 | 7 | + |  | + | + |
| O94811 | Tubulin polymerization-promoting protein | TPPP | 1 | 1 | 1 | 7.3 | 7.3 | 11 | 1 |  | + |  |  |
| O95716 | Ras-related protein Rab-3D | RAB3D | 1 | 2 | 1 | 13.7 | 5 | 22 | 3 |  | + |  |  |
| P00338 | L-lactate dehydrogenase A chain | LDHA | 11 | 7 | 7 | 26.8 | 26.8 | 121 | 40 | + | + | + | + |
| P00505-2 | Aspartate aminotransferase, mitochondrial | GOT2 | 2 | 2 | 2 | 10.3 | 10.3 | 44 | 4 | + | + |  |  |
| P00558-2 | Phosphoglycerate kinase 1 | PGK1 | 3 | 5 | 5 | 18.3 | 18.3 | 37 | 8 | + | + |  | + |
| P00918 | Carbonic anhydrase 2 | CA2 | 1 | 1 | 1 | 6.2 | 6.2 | 64 | 2 |  | + |  |  |
| P02042 | Hemoglobin subunit delta | HBD | 1 | 7 | 0 | 42.2 | 0 | 60 | 0 |  |  |  |  |
| P02671-2 | Fibrinogen alpha chain | FGA | 2 | 1 | 1 | 2.3 | 2.3 | 68 | 4 | + |  | + | + |
| P02763 | Alpha-1-acid glycoprotein 1 | ORM1 | 2 | 3 | 3 | 19.4 | 19.4 | 26 | 13 |  |  | + | + |
| P04040 | Catalase | CAT | 1 | 5 | 5 | 14.4 | 14.4 | 44 | 11 | + | + | + | + |
| P04080 | Cystatin-B | CSTB | 1 | 1 | 1 | 12.2 | 12.2 | 66 | 1 |  |  |  | + |
| P04083 | Annexin A1 | ANXA1 | 3 | 4 | 4 | 16.2 | 16.2 | 78 | 51 | + | + | + | + |
| P04350 | Tubulin beta-4A chain | TUBB4A | 15 | 10 | 0 | 40.1 | 0 | 10 | 0 |  |  |  |  |
| P04406-2 | Glyceraldehyde-3-phosphate dehydrogenase | GAPDH | 3 | 6 | 6 | 32.4 | 32.4 | 79 | 27 | + | + | + | + |
| P63096 | Guanine nucleotide-binding protein G(i) subunit alpha-1 | GNAI1 | 31 | 3 | 1 | 11 | 4.2 | 26 | 2 |  | + |  |  |
| P04920-2 | Anion exchange protein 2 | SLC4A2 | 3 | 1 | 1 | 0.9 | 0.9 | 61 | 1 | + | + |  |  |
| P05023-3 | Sodium/potassium-transporting ATPase subunit alpha-1 | ATP1A1 | 4 | 6 | 1 | 10.7 | 2.5 | 30 | 1 |  | + |  |  |
| P05109 | Protein S100-A8 | S100A8 | 1 | 9 | 9 | 62.4 | 62.4 | 323 | 416 | + | + | + | + |
| P05141 | ADP/ATP translocase 2 | SLC25A5 | 4 | 2 | 1 | 8.4 | 4.4 | 18 | 6 | + | + |  |  |
| P05164-2 | Myeloperoxidase | MPO | 4 | 35 | 31 | 53.1 | 47.5 | 323 | 573 | + | + | + | + |
| P05204 | Non-histone chromosomal protein HMG-17 | HMGN2 | 1 | 1 | 1 | 13.3 | 13.3 | 98 | 14 | + | + | + | + |
| P05386 | 60S acidic ribosomal protein P1 | RPLP1 | 1 | 1 | 1 | 14 | 14 | 63 | 2 | + | + |  |  |
| P05771 | Protein kinase C beta type | PRKCB | 4 | 2 | 2 | 3.7 | 3.7 | 17 | 3 | + | + |  |  |
| P06576 | ATP synthase subunit beta, mitochondrial | ATP5B | 5 | 11 | 11 | 35.5 | 35.5 | 191 | 27 | + | + |  |  |
| P06702 | Protein S100-A9 | S100A9 | 1 | 10 | 10 | 73.7 | 73.7 | 323 | 1076 | + | + | + | + |
| P06733 | Alpha-enolase | ENO1 | 11 | 11 | 3 | 37.8 | 4.6 | 117 | 22 | + | + | + |  |
| P07195 | L-lactate dehydrogenase B chain | LDHB | 4 | 2 | 2 | 10.5 | 10.5 | 90 | 3 | + | + |  |  |
| Q5JP53 | Tubulin beta chain | TUBB | 5 | 11 | 2 | 41.8 | 7.5 | 31 | 3 | + | + |  |  |
| P07737 | Profilin-1 | PFN1 | 3 | 3 | 3 | 28.6 | 28.6 | 34 | 17 | + | + | + | + |
| P07900 | Heat shock protein HSP 90-alpha | HSP90AA1 | 4 | 5 | 3 | 11.2 | 7.7 | 45 | 8 | + | + |  | + |
| P08133-2 | Annexin A6 | ANXA6 | 8 | 5 | 5 | 11.4 | 11.4 | 38 | 14 | + | + |  | + |
| P08238 | Heat shock protein HSP 90-beta | HSP90AB1 | 2 | 4 | 2 | 7.6 | 4 | 17 | 2 | + | + |  |  |
| P08246 | Neutrophil elastase | ELANE | 1 | 1 | 1 | 2.6 | 2.6 | 66 | 6 |  | + | + | + |
| P08311 | Cathepsin G | CTSG | 1 | 11 | 11 | 45.5 | 45.5 | 83 | 52 | + | + | + | + |
| P09104-2 | Gamma-enolase | ENO2 | 4 | 5 | 2 | 22.5 | 8.4 | 23 | 5 | + | + |  |  |
| P09471 | Guanine nucleotide-binding protein G(o) subunit alpha | GNAO1 | 6 | 3 | 1 | 11.6 | 3.7 | 31 | 6 | + | + |  |  |
| P09960-3 | Leukotriene A-4 hydrolase | LTA4H | 4 | 1 | 1 | 2.4 | 2.4 | 7 | 2 | + | + |  |  |
| Q99878 | Histone H2A type 1-J | HIST1H2AJ | 25 | 8 | 3 | 66.4 | 31.2 | 96 | 127 | + | + | + | + |
| P0CB47 | Putative upstream-binding factor 1-like protein 1 | UBTFL1 | 2 | 1 | 1 | 2.5 | 2.5 | 2 | 0 |  |  |  | + |
| P10153 | Non-secretory ribonuclease | RNASE2 | 1 | 1 | 1 | 9.3 | 9.3 | 98 | 7 | + | + | + | + |
| P10599 | Thioredoxin | TXN | 1 | 1 | 1 | 11.4 | 11.4 | 89 | 4 | + | + | + |  |
| P10809 | 60 kDa heat shock protein, mitochondrial | HSPD1 | 1 | 1 | 1 | 5.2 | 5.2 | 74 | 2 | + | + |  |  |
| P11021 | 78 kDa glucose-regulated protein | HSPA5 | 1 | 4 | 3 | 9.5 | 7 | 101 | 27 | + | + | + | + |
| P11137-2 | Microtubule-associated protein 2 | MAP2 | 4 | 1 | 1 | 2.5 | 2.5 | 94 | 40 | + | + | + | + |
| P11142-2 | Heat shock cognate 71 kDa protein | HSPA8 | 14 | 8 | 7 | 26.6 | 23.3 | 77 | 13 | + | + |  | + |
| P11215 | Integrin alpha-M | ITGAM | 3 | 4 | 4 | 3 | 3 | 27 | 16 | + | + | + | + |
| P11277-3 | Spectrin beta chain, erythrocytic | SPTB | 3 | 2 | 2 | 1 | 1 | 12 | 2 |  |  |  | + |
| P11678 | Eosinophil peroxidase | EPX | 1 | 5 | 1 | 6 | 1 | 66 | 7 |  | + | + |  |
| P12236 | ADP/ATP translocase 3 | SLC25A6 | 1 | 2 | 1 | 9.1 | 5 | 12 | 1 |  | + |  |  |
| P12277 | Creatine kinase B-type | CKB | 4 | 3 | 3 | 10.8 | 10.8 | 33 | 5 | + | + |  |  |
| P12429 | Annexin A3 | ANXA3 | 6 | 5 | 5 | 17 | 17 | 45 | 32 | + | + | + | + |
| P12532 | Creatine kinase U-type, mitochondrial | CKMT1A | 3 | 1 | 1 | 2.2 | 2.2 | 90 | 0 | + | + |  |  |
| P12724 | Eosinophil cationic protein | RNASE3 | 1 | 2 | 2 | 16.9 | 16.9 | 13 | 5 |  | + | + | + |
| P12814 | Alpha-actinin-1 | ACTN1 | 13 | 9 | 4 | 11.2 | 5.8 | 323 | 51 | + | + | + | + |
| P13473-2 | Lysosome-associated membrane glycoprotein 2 | LAMP2 | 4 | 2 | 2 | 4.6 | 4.6 | 32 | 9 |  | + | + | + |
| P13639 | Elongation factor 2 | EEF2 | 1 | 2 | 2 | 3.5 | 3.5 | 25 | 4 | + | + |  |  |
| P13796 | Plastin-2 | LCP1 | 2 | 2 | 2 | 4.3 | 4.3 | 50 | 2 |  | + |  | + |
| Q5T621 | Alcohol dehydrogenase [NADP(+)] | AKR1A1 | 2 | 1 | 1 | 15.6 | 15.6 | 10 | 1 |  | + |  |  |
| Q58FF3 | Putative endoplasmin-like protein | HSP90B2P | 2 | 1 | 1 | 2.5 | 2.5 | 10 | 1 |  |  |  | + |
| P16402 | Histone H1.3 | HIST1H1D | 1 | 1 | 1 | 7.2 | 7.2 | 12 | 1 | + |  |  |  |
| P16989-2 | Y-box-binding protein 3 | YBX3 | 3 | 1 | 1 | 3.6 | 3.6 | 63 | 2 | + | + |  |  |
| P17600-2 | Synapsin-1 | SYN1 | 2 | 3 | 3 | 8.4 | 8.4 | 54 | 3 | + | + |  |  |
| P18206-2 | Vinculin | VCL | 2 | 2 | 2 | 3 | 3 | 12 | 1 |  | + |  | + |
| P18669 | Phosphoglycerate mutase 1 | PGAM1 | 3 | 4 | 4 | 29.1 | 29.1 | 139 | 7 | + | + |  |  |
| P19022-2 | Cadherin-2 | CDH2 | 2 | 1 | 1 | 1.3 | 1.3 | 64 | 1 |  | + |  |  |
| P19367-4 | Hexokinase-1 | HK1 | 7 | 2 | 2 | 3.2 | 3.2 | 17 | 3 | + | + |  |  |
| P20160 | Azurocidin | AZU1 | 1 | 3 | 3 | 12.4 | 12.4 | 61 | 50 | + | + | + | + |
| P20336 | Ras-related protein Rab-3A | RAB3A | 3 | 2 | 1 | 15.5 | 6.8 | 71 | 1 | + |  |  |  |
| P20674 | Cytochrome c oxidase subunit 5A, mitochondrial | COX5A | 5 | 2 | 2 | 30.7 | 30.7 | 33 | 1 |  | + |  |  |
| Q60FE5 | Filamin-A | FLNA | 9 | 13 | 13 | 8.8 | 8.8 | 154 | 46 | + | + | + | + |
| Q5JRR6 | Ubiquitin-like modifier-activating enzyme 1 | UBA1 | 3 | 1 | 1 | 4.2 | 4.2 | 10 | 1 |  | + |  |  |
| P24158 | Myeloblastin | PRTN3 | 2 | 3 | 3 | 11.3 | 11.3 | 20 | 14 |  | + | + | + |
| P25705-2 | ATP synthase subunit alpha, mitochondrial | ATP5A1 | 9 | 7 | 7 | 20.5 | 20.5 | 71 | 12 | + | + |  |  |
| P26038 | Moesin | MSN | 1 | 2 | 2 | 1.9 | 1.9 | 12 | 1 | + |  |  | + |
| P26583 | High mobility group protein B2 | HMGB2 | 6 | 3 | 3 | 14.8 | 14.8 | 47 | 44 | + | + | + | + |
| P28324-2 | ETS domain-containing protein Elk-4 | ELK4 | 2 | 1 | 1 | 2 | 2 | 2 | 11 | + | + | + | + |
| P29350-2 | Tyrosine-protein phosphatase non-receptor type 6 | PTPN6 | 4 | 1 | 1 | 2 | 2 | 62 | 1 | + |  |  |  |
| P29966 | Myristoylated alanine-rich C-kinase substrate | MARCKS | 1 | 1 | 1 | 5.1 | 5.1 | 66 | 1 | + |  |  |  |
| P30044-4 | Peroxiredoxin-5, mitochondrial | PRDX5 | 4 | 1 | 1 | 13.6 | 13.6 | 26 | 1 | + |  |  |  |
| P30740 | Leukocyte elastase inhibitor | SERPINB1 | 2 | 8 | 8 | 27.2 | 27.2 | 153 | 50 |  | + | + | + |
| P31146 | Coronin-1A | CORO1A | 2 | 3 | 3 | 9.5 | 9.5 | 35 | 6 | + | + | + |  |
| Q5SX91 | Rab GDP dissociation inhibitor beta | GDI2 | 5 | 2 | 2 | 18.7 | 18.7 | 39 | 5 | + | + |  |  |
| P31946-2 | 14-3-3 protein beta/alpha | YWHAB | 2 | 4 | 2 | 22.1 | 15.2 | 32 | 3 | + | + |  |  |
| P31949 | Protein S100-A11 | S100A11 | 1 | 2 | 2 | 21.9 | 21.9 | 14 | 6 | + | + | + | + |
| P31997 | Carcinoembryonic antigen-related cell adhesion molecule 8 | CEACAM8 | 15 | 3 | 3 | 12.3 | 12.3 | 60 | 43 | + | + | + | + |
| P35579 | Myosin-9 | MYH9 | 17 | 17 | 17 | 9.1 | 9.1 | 141 | 91 | + | + | + | + |
| X6RJP6 | Transgelin-2 | TAGLN2 | 3 | 1 | 1 | 7.5 | 7.5 | 65 | 2 | + | + |  |  |
| P38606-2 | V-type proton ATPase catalytic subunit A | ATP6V1A | 2 | 1 | 1 | 3.1 | 3.1 | 25 | 2 | + | + |  |  |
| P38646 | Stress-70 protein, mitochondrial | HSPA9 | 1 | 1 | 1 | 3.1 | 3.1 | 72 | 1 |  | + |  |  |
| P40199 | Carcinoembryonic antigen-related cell adhesion molecule 6 | CEACAM6 | 2 | 2 | 2 | 9.9 | 9.9 | 24 | 35 | + | + | + | + |
| P40926 | Malate dehydrogenase, mitochondrial | MDH2 | 3 | 4 | 4 | 16.6 | 16.6 | 53 | 8 | + | + |  |  |
| Q5T4L4 | 40S ribosomal protein S27 | RPS27 | 2 | 2 | 2 | 18.2 | 18.2 | 25 | 3 |  | + |  | + |
| P43004-3 | Excitatory amino acid transporter 2 | SLC1A2 | 6 | 3 | 3 | 6.4 | 6.4 | 38 | 9 | + | + |  |  |
| P50552 | Vasodilator-stimulated phosphoprotein | VASP | 2 | 2 | 2 | 6.3 | 6.3 | 15 | 5 | + | + |  | + |
| P52907 | F-actin-capping protein subunit alpha-1 | CAPZA1 | 1 | 1 | 1 | 6.3 | 6.3 | 75 | 1 |  |  |  | + |
| P57052-2 | Splicing regulator RBM11 | RBM11 | 2 | 1 | 1 | 3 | 3 | 2 | 2 |  | + |  |  |
| P60174-4 | Triosephosphate isomerase | TPI1 | 5 | 2 | 2 | 26.3 | 26.3 | 21 | 3 | + | + |  |  |
| P60201 | Myelin proteolipid protein | PLP1 | 5 | 2 | 2 | 8.7 | 8.7 | 14 | 6 | + | + |  |  |
| P60709 | Actin, cytoplasmic 1 | ACTB | 13 | 18 | 1 | 68.3 | 4.5 | 293 | 259 | + | + |  | + |
| P61266-2 | Syntaxin-1B | STX1B | 2 | 1 | 1 | 5.4 | 5.4 | 10 | 2 | + | + |  |  |
| P61604 | 10 kDa heat shock protein, mitochondrial | HSPE1 | 4 | 6 | 6 | 59.8 | 59.8 | 38 | 14 | + | + | + | + |
| P61978-3 | Heterogeneous nuclear ribonucleoprotein K | HNRNPK | 5 | 3 | 3 | 8.9 | 8.9 | 21 | 4 | + | + |  |  |
| P61981 | 14-3-3 protein gamma | YWHAG | 1 | 4 | 2 | 22.3 | 15.4 | 20 | 4 | + | + |  |  |
| P62258 | 14-3-3 protein epsilon | YWHAE | 6 | 5 | 3 | 23.9 | 17.3 | 31 | 5 | + | + |  |  |
| P62328 | Thymosin beta-4 | TMSB4X | 1 | 1 | 1 | 25 | 25 | 15 | 15 | + | + |  |  |
| P62701 | 40S ribosomal protein S4, X isoform | RPS4X | 1 | 1 | 1 | 6.5 | 6.5 | 88 | 2 | + | + |  |  |
| P62760 | Visinin-like protein 1 | VSNL1 | 1 | 1 | 1 | 6.3 | 6.3 | 63 | 2 | + | + |  |  |
| P62805 | Histone H4 | HIST1H4A | 1 | 6 | 6 | 51.5 | 51.5 | 87 | 48 | + | + | + | + |
| P62873-2 | Guanine nucleotide-binding protein G(I)/G(S)/G(T) subunit beta-1 | GNB1 | 3 | 2 | 1 | 8.7 | 5.4 | 67 | 1 | + |  |  |  |
| P62937 | Peptidyl-prolyl cis-trans isomerase A | PPIA | 11 | 2 | 2 | 15.2 | 15.2 | 13 | 3 | + | + |  |  |
| P63104 | 14-3-3 protein zeta/delta | YWHAZ | 22 | 6 | 4 | 34.7 | 27.8 | 54 | 11 | + | + |  |  |
| P63261 | Actin, cytoplasmic 2 | ACTG1 | 7 | 18 | 1 | 68.3 | 4.5 | 24 | 4 | + | + |  |  |
| P68133 | Actin, alpha skeletal muscle | ACTA1 | 10 | 11 | 1 | 29.7 | 4.2 | 17 | 29 | + | + | + | + |
| Q5VTE0 | Putative elongation factor 1-alpha-like 3 | EEF1A1P5 | 6 | 4 | 4 | 15.6 | 15.6 | 55 | 5 | + | + |  |  |
| P68363 | Tubulin alpha-1B chain | TUBA1B | 3 | 13 | 0 | 52.3 | 0 | 192 | 40 |  |  |  |  |
| P68366-2 | Tubulin alpha-4A chain | TUBA4A | 6 | 11 | 1 | 42.5 | 3.5 | 18 | 3 | + | + |  |  |
| P68371 | Tubulin beta-4B chain | TUBB4B | 3 | 12 | 0 | 46.5 | 0 | 48 | 8 |  |  |  |  |
| P68871 | Hemoglobin subunit beta | HBB | 10 | 9 | 3 | 63.9 | 30.6 | 323 | 119 | + | + | + | + |
| P69905 | Hemoglobin subunit alpha | HBA1 | 1 | 4 | 1 | 36.6 | 10.6 | 103 | 80 | + | + | + | + |
| P78324-4 | Tyrosine-protein phosphatase non-receptor type substrate 1 | SIRPA | 3 | 1 | 1 | 2.6 | 2.6 | 15 | 1 |  |  | + |  |
| P80511 | Protein S100-A12 | S100A12 | 1 | 4 | 4 | 29.3 | 29.3 | 154 | 53 | + | + | + | + |
| P80723 | Brain acid soluble protein 1 | BASP1 | 3 | 4 | 4 | 29.5 | 29.5 | 45 | 10 | + |  | + |  |
| P81605 | Dermcidin | DCD | 2 | 1 | 1 | 10 | 10 | 61 | 3 | + | + |  |  |
| P86791 | Vacuolar fusion protein CCZ1 homolog | CCZ1 | 2 | 1 | 1 | 2.9 | 2.9 | 66 | 6 |  | + |  | + |
| Q03001-3 |  | DST | 1 | 1 | 1 | 0.4 | 0.4 | 2 | 1 |  |  |  | + |
| Q04760-2 | Lactoylglutathione lyase | GLO1 | 2 | 1 | 1 | 11.8 | 11.8 | 13 | 1 | + |  |  |  |
| Q12860-2 | Contactin-1 | CNTN1 | 3 | 2 | 2 | 2.7 | 2.7 | 20 | 4 | + | + |  |  |
| Q13023 | A-kinase anchor protein 6 | AKAP6 | 1 | 1 | 1 | 0.6 | 0.6 | 2 | 1 |  | + |  |  |
| Q13367-2 | AP-3 complex subunit beta-2 | AP3B2 | 4 | 1 | 1 | 5.5 | 5.5 | 60 | 4 |  | + |  | + |
| Q13554 | Calcium/calmodulin-dependent protein kinase type II subunit beta | CAMK2B | 40 | 3 | 2 | 16.5 | 12.2 | 41 | 2 | + | + |  |  |
| Q13885 | Tubulin beta-2A chain | TUBB2A | 11 | 13 | 3 | 48.8 | 12.1 | 323 | 42 | + | + |  |  |
| Q15464 | SH2 domain-containing adapter protein B | SHB | 1 | 1 | 1 | 1.8 | 1.8 | 2 | 1 |  | + |  |  |
| Q16352 | Alpha-internexin | INA | 8 | 2 | 2 | 4.6 | 4.6 | 16 | 3 | + | + |  |  |
| Q16658 | Fascin | FSCN1 | 1 | 1 | 1 | 3.7 | 3.7 | 92 | 1 | + |  |  |  |
| Q562R1 | Beta-actin-like protein 2 | ACTBL2 | 1 | 5 | 1 | 13.8 | 2.7 | 2 | 1 | + |  |  |  |
| Q5K130 | Putative chronic lymphocytic leukemia up-regulated protein 1 opposite strand transcript protein | CLLU1OS | 1 | 1 | 1 | 8.9 | 8.9 | 2 | 3 |  |  |  | + |
| Q5SWH9 | Transmembrane protein 69 | TMEM69 | 1 | 1 | 1 | 4.9 | 4.9 | 62 | 1 |  |  |  | + |
| Q5SZK5 | Wiskott-Aldrich syndrome protein family member 1 | WASF1 | 4 | 2 | 2 | 17.8 | 17.8 | 18 | 2 | + | + |  |  |
| Q5W0U4-2 | B box and SPRY domain-containing protein | BSPRY | 2 | 1 | 1 | 3.6 | 3.6 | 66 | 0 |  |  |  | + |
| Q6PP77-2 | XK-related protein 2 | XKRX | 2 | 1 | 1 | 4.5 | 4.5 | 65 | 13 | + | + | + | + |
| Q71U36-2 | Tubulin alpha-1A chain | TUBA1A | 6 | 13 | 1 | 56.7 | 3.4 | 25 | 9 | + | + |  |  |
| Q7Z333-3 | Probable helicase senataxin | SETX | 3 | 1 | 1 | 0.3 | 0.3 | 71 | 0 |  | + |  |  |
| Q7Z442-2 | Polycystic kidney disease protein 1-like 2 | PKD1L2 | 2 | 1 | 1 | 0.7 | 0.7 | 61 | 1 |  | + |  |  |
| Q8IUE6 | Histone H2A type 2-B | HIST2H2AB | 1 | 5 | 1 | 45.4 | 5.4 | 64 | 3 |  | + |  |  |
| Q8IZU3 | Synaptonemal complex protein 3 | SYCP3 | 1 | 1 | 1 | 4.2 | 4.2 | 2 | 0 | + |  |  |  |
| Q8NC51-4 | Plasminogen activator inhibitor 1 RNA-binding protein | SERBP1 | 4 | 4 | 4 | 12.7 | 12.7 | 29 | 12 | + | + | + | + |
| Q8NEE8 | Tetratricopeptide repeat protein 16 | TTC16 | 1 | 1 | 1 | 1.3 | 1.3 | 2 | 1 | + |  |  |  |
| Q8TE73 | Dynein heavy chain 5, axonemal | DNAH5 | 1 | 1 | 1 | 0.2 | 0.2 | 62 | 7 |  | + | + | + |
| Q92878-3 | DNA repair protein RAD50 | RAD50 | 3 | 1 | 1 | 1.7 | 1.7 | 7 | 1 |  |  |  | + |
| Q9BRH9 | Zinc finger protein 251 | ZNF251 | 1 | 1 | 1 | 1.6 | 1.6 | 2 | 0 |  |  |  | + |
| Q9H156 | SLIT and NTRK-like protein 2 | SLITRK2 | 1 | 1 | 1 | 0.9 | 0.9 | 67 | 112 | + | + | + | + |
| Q9UQM7 | Calcium/calmodulin-dependent protein kinase type II subunit alpha | CAMK2A | 5 | 6 | 5 | 22.6 | 18.6 | 159 | 13 | + | + |  |  |
| Q9Y490 | Talin-1 | TLN1 | 1 | 2 | 2 | 1.3 | 1.3 | 27 | 3 |  | + |  | + |
| Q9Y6Q9-4 | Nuclear receptor coactivator 3 | NCOA3 | 5 | 1 | 1 | 1 | 1 | 79 | 1 | + |  |  |  |
