# Supplementary material for: Neutrophil extracellular traps (NET) induced by different stimuli: A comparative proteomic analysis
Source: PLoS One. 2019 Jul 8;14(7):e0218946. doi: 10.1371/journal.pone.0218946 (PMC6613696; doi:10.1371/journal.pone.0218946)
Supplement: S2 Table — The symbol “+” identify the post-translational modifications present in each experimental condition (Fig 7). (DOCX) [file pone.0218946.s004.docx]

**Supplementary Table 2**. List of all post-translational modifications identified by mass spectrometry using dependent peptide (DP) and variable modification (VM) methods. The symbol “+” identify the post-translational modifications present in each experimental condition (Figure 7).

| Uniprot ID | Protein name | Gene name | Sequence window for Variable Modification | Peptides for Dependent Peptide | Isotype | AA | Type | Spontaneous | PMA | LPS | A23187 | Classification |
| --- | --- | --- | --- | --- | --- | --- | --- | --- | --- | --- | --- | --- |
| A0A0U1RR27 | C-myc promoter-binding protein | DENND4A |  | NNLFER | Citrullination | nterm | D | + | + |  |  | DP |
| E7EQB2 | Kaliocin-1 | LTF |  | EDAIWNLLR | Citrullination | nterm | D |  | + | + |  | DP |
| O15541 | RING finger protein 113A | RNF113A |  | DAQAIFER | Citrullination | nterm | D |  | + | + | + | DP |
| P05109 | Protein S100-A8 | S100A8 |  | GNFHAVYR | Citrullination | nterm | D | + |  |  | + | DP |
| P05164 | 84 kDa myeloperoxidase | MPO |  | NGFPVALAR | Citrullination | nterm | D |  |  | + | + | DP |
| P05164 | 84 kDa myeloperoxidase | MPO |  | NNIFMSNSYPR | Citrullination | nterm | D |  | + | + |  | DP |
| P08311 | Cathepsin G | CTSG |  | HPQYNQR | Citrullination | nterm | D |  |  |  | + | DP |
| P11215 | Integrin alpha-M | ITGAM |  | LPSHSDFLAELR | Citrullination | nterm | D | + |  | + |  | DP |
| P62805 | Histone H4 | HIST1H4A |  | ISGLIYEETR | Citrullination | nterm | D |  | + |  |  | DP |
| Q9Y3C8 | Ubiquitin-fold modifier-conjugating enzyme 1 | UFC1 |  | TNAGPRDR | Citrullination | cterm;D;G;P;R | D | + |  |  | + | DP |
| A0A024R571 | EH domain-containing protein 1 | EHD1 |  | HLIEQDFPGMR | Reduction | cterm;F;G;M;P;R | Other |  |  |  | + | DP |
| A0A024R571 | EH domain-containing protein 1 | EHD1 |  | EHQISPGDFPSLR | Peptide:I.EREHQISPGDFPSLR | nterm | Other |  | + |  |  | DP |
| A0A075B724 | Tubulin beta-4A chain | TUBB4A |  | AVLVDLEPGTMDSVR | Methylation | nterm | Other |  | + |  |  | DP |
| A0A075B736 | Tubulin beta chain | TUBB |  | FPGQLNADLR | Comp.:O-NH3 | nterm | Other |  | + |  |  | DP |
| A0A075B736 | Tubulin beta chain | TUBB |  | LAVNMVPFPR | Oxidation | M;N | Other |  | + |  |  | DP |
| A0A075B736 | Tubulin beta chain | TUBB |  | LHFFMPGFAPLTSR | Diffuse:2 | A;F | Other |  | + |  |  | DP |
| A0A087WSZ2 | Alpha-actinin-1 | ACTN1 |  | HEAFESDLAAHQDR | Diffuse:2 | nterm | Other |  | + |  |  | DP |
| A0A087WTB6 | Guanine nucleotide-binding protein G(i) subunit alpha-1 | GNAI1 |  | LLLLGAGESGK | Peptide:L.LLGAGESGK | L | Other |  | + |  |  | DP |
| A0A087WTB6 | Guanine nucleotide-binding protein G(i) subunit alpha-1 | GNAI1 |  | LLLLGAGESGK | Peptide:L.LLLGAGESGK | L | Other |  | + |  |  | DP |
| A0A087WTB6 | Guanine nucleotide-binding protein G(i) subunit alpha-1 | GNAI1 |  | LLLLGAGESGK | V | A;cterm;E;G;K;L;S | Other |  | + |  |  | DP |
| A0A087WTB6 | Guanine nucleotide-binding protein G(i) subunit alpha-1 | GNAI1 |  | LLLLGAGESGK | Methylation | A;cterm;E;G;K;S | Other |  | + |  |  | DP |
| A0A087WTK0 | Protein-tyrosine-phosphatase | PTPRJ |  | VLLESIGSHEELTQDSR | Comp.:13C-12C | nterm | Other |  | + |  | + | DP |
| A0A087WV01 | Elongation factor 1-alpha | EEF1A1 |  | DGNASGTTLLEALDCILPPTRPTDK | Comp.:H4C3 | nterm | Other | + |  |  |  | DP |
| A0A087WVQ6 | Clathrin heavy chain | CLTC |  | GYFEELITMLEAALGLER | Reduction | L;M;T | Other |  | + |  |  | DP |
| A0A087WW55 | Alpha-trypsin chain 1 | PRSS1 |  | TLNNDIMLIKLSSRAVINAR | Reduction | A;cterm;I;K;L;M;N;R;S;V | Other | + | + | + |  | DP |
| A0A087WW55 | Alpha-trypsin chain 1 | PRSS1 |  | TLNNDIMLIKLSSRAVINAR | GlyGly | nterm | Other | + |  |  |  | DP |
| A0A087WWT3 | Serum albumin | ALB |  | VPQVSTPTLVEVSR | Formylation | nterm | Other |  | + |  |  | DP |
| A0A087WWU8 | Tropomyosin alpha-3 chain | DKFZp686J1372 |  | HIAEEADR | G | A;D | Other | + |  |  | + | DP |
| A0A087WWU8 | Tropomyosin alpha-3 chain | DKFZp686J1372 |  | HIAEEADR | Comp.:-H4O2 | E | Other |  |  |  | + | DP |
| A0A087X1X7 | Elongation factor 1-delta | EEF1D |  | IASLEVENQSLR | Peptide:A.SLEVENQSLR | nterm | Other |  |  | + | + | DP |
| A0A087X1X7 | Elongation factor 1-delta | EEF1D |  | IASLEVENQSLR | Peptide:I.ASLEVENQSLR | L | Other |  |  | + | + | DP |
| A0A0A0MT68 | Deoxyribonuclease | DNASE1L3 |  | STNCAYDR | Comp.:-H5C2NOS | C | Other | + | + | + | + | DP |
| A0A0A0MT68 | Deoxyribonuclease | DNASE1L3 |  | STNCAYDR | Peptide:S.TNCAYDR | nterm | Other | + |  |  |  | DP |
| A0A0A0MT68 | Deoxyribonuclease | DNASE1L3 |  | STNCAYDR | G | C | Other |  |  |  | + | DP |
| A0A0A0MT68 | Deoxyribonuclease | DNASE1L3 |  | STNCAYDR | Comp.:-CH4O | C | Other | + | + |  | + | DP |
| A0A0A0MT68 | Deoxyribonuclease | DNASE1L3 |  | STNCAYDR | Comp.:12C-13C | N | Other | + | + | + | + | DP |
| A0A0A0MT68 | Deoxyribonuclease | DNASE1L3 |  | STNCAYDR | Deamidation | N | Other | + | + |  | + | DP |
| A0A0A0MT68 | Deoxyribonuclease | DNASE1L3 |  | STNCAYDR | Comp.:C | nterm | Other |  | + |  |  | DP |
| A0A0A0MT68 | Deoxyribonuclease | DNASE1L3 |  | STNCAYDR | Comp.:HN | C;N | Other | + |  |  |  | DP |
| A0A0A0MT68 | Deoxyribonuclease | DNASE1L3 |  | STNCAYDR | Oxidation | C | Other |  |  | + |  | DP |
| A0A0A0MT68 | Deoxyribonuclease | DNASE1L3 |  | STNCAYDR | Sulfide | C | Other | + | + | + | + | DP |
| A0A0A0MT68 | Deoxyribonuclease | DNASE1L3 |  | STNCAYDR | Comp.:C4H8O3 | C | Other | + |  |  |  | DP |
| A0A0A0MT68 | Deoxyribonuclease | DNASE1L3 |  | STNCAYDR | Peptide:STNCAYDRI.V | cterm;R | Other | + | + | + | + | DP |
| A0A0A0MT68 | Deoxyribonuclease | DNASE1L3 |  | STNCAYDR | Comp.:C10H11O2NS | C | Other |  | + |  |  | DP |
| A0A0B4J269 | Tubulin beta chain | TUBB |  | AILVDLEPGTMDSVR | Comp.:-H4O2 | nterm | Other | + |  |  |  | DP |
| A0A0B4J269 | Tubulin beta chain | TUBB |  | ISEQFTAMFR | Reduction | A;cterm;F;M;R | Other | + | + |  |  | DP |
| A0A0C4DGB6 | Serum albumin | ALB |  | LVTDLTK | Peptide:S.KLVTDLTK | nterm | Other |  |  | + | + | DP |
| A0A0C4DGJ9 | Granzyme H | GZMH |  | GDSGGPLVCK | Peptide:D.SGGPLVCK | S | Other |  |  |  | + | DP |
| A0A0C4DGJ9 | Granzyme H | GZMH |  | GDSGGPLVCK | Comp.:-H5C2NOS | C;cterm;K | Other | + |  |  |  | DP |
| A0A0C4DGJ9 | Granzyme H | GZMH |  | GDSGGPLVCK | Oxidation | C;cterm;K | Other | + |  |  |  | DP |
| A0A0C4DGJ9 | Granzyme H | GZMH |  | GDSGGPLVCK | Sulfide | C | Other | + | + |  |  | DP |
| A0A0C4DGW9 | Serpin I2 | SERPINI2 |  | DMFSGEEFGPLTR | Reduction | nterm | Other |  |  |  | + | DP |
| A0A0C4DGW9 | Serpin I2 | SERPINI2 |  | IKDMFSGEEFGPLTR | Oxidation | nterm | Other |  | + |  |  | DP |
| A0A0G2JIW1 | Heat shock 70 kDa protein 1A | HSPA1A |  | ATAGDTHLGGEDFDNR | Oxidation | nterm | Other |  |  | + |  | DP |
| A0A0G2JIW1 | Heat shock 70 kDa protein 1A | HSPA1A |  | AQIHDLVLVGGSTR | Oxidation | nterm | Other |  |  |  | + | DP |
| A0A0J9YWK4 | Hemoglobin subunit beta | HBB |  | VHLTPEEK | Peptide:V.HLTPEEK | nterm | Other | + | + | + | + | DP |
| A0A0J9YWK4 | Hemoglobin subunit beta | HBB |  | VHLTPEEK | Deamidation | nterm | Other |  |  | + |  | DP |
| A0A0J9YWK4 | Hemoglobin subunit beta | HBB |  | VHLTPEEK | Diffuse:2 | nterm | Other |  |  | + |  | DP |
| A0A0J9YWK4 | Hemoglobin subunit beta | HBB |  | VHLTPEEK | Comp.:C | H | Other | + | + | + | + | DP |
| A0A0J9YWK4 | Hemoglobin subunit beta | HBB |  | VHLTPEEK | Acetaldehyde | H | Other |  | + |  | + | DP |
| A0A0J9YWK4 | Hemoglobin subunit beta | HBB |  | VHLTPEEK | tri-Methylation | E | Other |  | + | + | + | DP |
| A0A0J9YWK4 | Hemoglobin subunit beta | HBB |  | VHLTPEEK | GlyGly | nterm | Other | + | + | + | + | DP |
| A0A0U1RR27 | C-myc promoter-binding protein | DENND4A |  | NNLFER | Acetaldehyde | nterm | Other |  | + |  |  | DP |
| A0A0U1RR27 | C-myc promoter-binding protein | DENND4A |  | NNLFER | tri-Methylation | E | Other | + | + |  |  | DP |
| A0A0U1RR32 | Histone H2A | H2AFJ |  | HLQLAIR | Comp.:-H4O2 | I | Other | + | + | + | + | DP |
| A0A0U1RR32 | Histone H2A | H2AFJ |  | HLQLAIR | Deamidation | Q | Other | + | + | + | + | DP |
| A0A0U1RR32 | Histone H2A | H2AFJ |  | HLQLAIR | Comp.:C | nterm | Other | + | + |  |  | DP |
| A0A0U1RR32 | Histone H2A | H2AFJ |  | HLQLAIR | Formylation | nterm | Other |  | + |  | + | DP |
| A0A0U1RR32 | Histone H2A | H2AFJ |  | AGLQFPVGR | Peptide:A.GLQFPVGR | nterm | Other | + | + |  |  | DP |
| A0A0U1RR32 | Histone H2A | H2AFJ |  | AGLQFPVGR | Comp.:-H4O2 | nterm | Other | + | + |  |  | DP |
| A0A0U1RR32 | Histone H2A | H2AFJ |  | AGLQFPVGR | Comp.:H4C3 | nterm | Other | + |  |  |  | DP |
| A0A0U1RR32 | Histone H2A | H2AFJ |  | NDEELNK | Reduction | cterm;E;K;L;N | Other |  | + |  |  | DP |
| A0A0U1RR32 | Histone H2A | H2AFJ |  | NDEELNK | Deamidation | nterm | Other | + |  |  | + | DP |
| A0A0U1RR32 | Histone H2A | H2AFJ |  | NDEELNK | Oxidation | E;L;N | Other | + | + |  | + | DP |
| A0A0U1RR32 | Histone H2A | H2AFJ |  | NDEELNK | Comp.:N2 | cterm;K | Other | + | + | + | + | DP |
| A0A0U1RR32 | Histone H2A | H2AFJ |  | NDEELNK | Peptide:NDEELNKL.L | cterm;K | Other | + | + | + | + | DP |
| A0A0U1RR32 | Histone H2A | H2AFJ |  | NDEELNK | Peptide:I.RNDEELNK | nterm | Other | + | + | + | + | DP |
| A0A0U1RR32 | Histone H2A | H2AFJ |  | NDEELNK | R | cterm;K | Other | + | + | + | + | DP |
| A0A0U1RR32 | Histone H2A | H2AFJ |  | NDEELNK | Peptide:A.IRNDEELNK | nterm | Other |  |  | + |  | DP |
| A0A0U1RR32 | Histone H2A | H2AFJ |  | NDEELNK | Peptide:L.AVRNDEELNK | nterm | Other | + | + | + | + | DP |
| A0A0U1RR32 | Histone H2A | H2AFJ |  | HLQLAIRNDEELNK | Peptide:A.IRNDEELNK | nterm | Other |  | + | + |  | DP |
| A0A0U1RR32 | Histone H2A | H2AFJ |  | HLQLAIRNDEELNK | Peptide:HLQLAIRNDEELN.K | cterm;K;N | Other |  | + |  |  | DP |
| A0A0U1RR32 | Histone H2A | H2AFJ |  | VTIAQGGVLPNIQAVLLPK | Comp.:C | nterm | Other | + | + |  |  | DP |
| A0A0U1RR32 | Histone H2A type 1 | HIST1H2AB |  | KTESHHK | Loss of ammonia | E;H;S | Other |  |  |  | + | DP |
| A0A0U1RR32 | Histone H2A type 1 | HIST1H2AB |  | KTESHHK | Comp.:13C-12C | nterm | Other |  |  |  | + | DP |
| A0A0U1RRE1 | Septin-7 | SEPT7 |  | QMPDNRVQCCLYFIAPSGHGLK | Peptide:QMPDNRVQCCLYFIAPSGHGL.K | nterm | Other |  |  | + |  | DP |
| A0A0U1RRE1 | Septin-7 | SEPT7 |  | QMPDNRVQCCLYFIAPSGHGLK | G | nterm | Other |  |  |  | + | DP |
| A0A1B0GUA0 | Vimentin | VIM |  | LGDLYEEEMR | Reduction | cterm;E;M;R | Other |  |  | + |  | DP |
| A0A1W2PQM2 | Tubulin alpha-1A chain | TUBA1A |  | VGINYQPPTVVPGGDLAK | Peptide:I.NYQPPTVVPGGDLAK | nterm | Other | + |  |  |  | DP |
| A0A1W2PQM2 | Tubulin alpha-1A chain | TUBA1A |  | VGINYQPPTVVPGGDLAK | Diffuse:2 | P;Q | Other | + |  |  |  | DP |
| A0A1W2PQM2 | Tubulin alpha-1A chain | TUBA1A |  | EIIDLVLDR | Comp.:-CH4 | L | Other | + |  |  |  | DP |
| A0A1W2PQM2 | Tubulin alpha-1A chain | TUBA1A |  | DVNAAIATIK | Comp.:C-N3 | A;cterm;I;K;T | Other | + |  |  |  | DP |
| A2A369 | Dedicator of cytokinesis protein 8 | DOCK8 |  | KNYNKLK | Peptide:KNYNKL.K | K;L | Other |  |  |  | + | DP |
| A2A369 | Dedicator of cytokinesis protein 8 | DOCK8 |  | KNYNKLK | Comp.:N2 | N;Y | Other |  |  | + | + | DP |
| A5A3E0 | Actin, alpha cardiac muscle 1 | ACTA1 |  | AGFAGDDAPR | Peptide:G.FAGDDAPR | A;F | Other |  |  | + | + | DP |
| A5A3E0 | Actin, alpha cardiac muscle 1 | ACTA1 |  | AGFAGDDAPR | Peptide:A.GFAGDDAPR | A | Other | + | + | + | + | DP |
| A5A3E0 | Actin, alpha cardiac muscle 1 | ACTA1 |  | AGFAGDDAPR | Comp.:-H4O2 | nterm | Other | + | + | + | + | DP |
| A5A3E0 | Actin, alpha cardiac muscle 1 | ACTA1 |  | AGFAGDDAPR | Acetaldehyde | nterm | Other | + | + |  | + | DP |
| A5A3E0 | Actin, alpha cardiac muscle 1 | ACTA1 |  | AGFAGDDAPR | Peptide:C.KAGFAGDDAPR | nterm | Other |  |  |  | + | DP |
| A5A3E0 | Actin, alpha cardiac muscle 1 | ACTA1 |  | AVFPSIVGR | Loss of ammonia | cterm;G;I;R;S;V | Other |  | + |  |  | DP |
| A6NL76 | Actin, alpha cardiac muscle 1 | ACTA1 |  | DSYVGDEAQSK | Peptide:Y.VGDEAQSK | nterm | Other |  |  |  | + | DP |
| A6NL76 | Actin, alpha cardiac muscle 1 | ACTA1 |  | DSYVGDEAQSK | Peptide:D.SYVGDEAQSK | nterm | Other |  | + |  |  | DP |
| A6NL76 | Actin, alpha cardiac muscle 1 | ACTA1 |  | DSYVGDEAQSK | Acetaldehyde | nterm | Other | + |  |  |  | DP |
| A6NL76 | Actin, alpha cardiac muscle 1 | ACTA1 |  | DSYVGDEAQSK | tri-Methylation | A;cterm;K;Q;S | Other | + |  |  |  | DP |
| A6NL76 | Actin, alpha cardiac muscle 1 | ACTA1 |  | DSYVGDEAQSK | Peptide:Q.KDSYVGDEAQSK | nterm | Other |  |  |  | + | DP |
| A6NL76 | Actin, alpha cardiac muscle 1 | ACTA1 |  | DSYVGDEAQSK | Peptide:DSYVGDEAQSKRG.I | cterm;K;S | Other | + | + | + | + | DP |
| A6NL76 | Actin, alpha cardiac muscle 1 | ACTA1 |  | HQGVMVGMGQK | Reduction | G;M | Other |  |  | + | + | DP |
| A6NL76 | Actin, alpha cardiac muscle 1 | ACTA1 |  | YPIEHGIITNWDDMEK | Peptide:I.EHGIITNWDDMEK | nterm | Other |  | + | + | + | DP |
| A6NL76 | Actin, alpha cardiac muscle 1 | ACTA1 |  | YPIEHGIITNWDDMEK | Reduction | M | Other |  |  |  | + | DP |
| A6NL76 | Actin, alpha cardiac muscle 1 | ACTA1 |  | YPIEHGIITNWDDMEK | Comp.:13C-12C | nterm | Other |  | + |  |  | DP |
| A6NL76 | Actin, alpha cardiac muscle 1 | ACTA1 |  | YPIEHGIITNWDDMEK | Diffuse:2 | nterm | Other |  | + |  |  | DP |
| A6NL76 | Actin, alpha cardiac muscle 1 | ACTA1 |  | YPIEHGIITNWDDMEK | Oxidation | cterm;D;E;K;M | Other |  | + |  | + | DP |
| A6NL76 | Actin, alpha cardiac muscle 1 | ACTA1 |  | YPIEHGIITNWDDMEK | Peptide:L.KYPIEHGIITNWDDMEK | nterm | Other |  | + |  | + | DP |
| A6NL76 | Actin, alpha cardiac muscle 1 | ACTA1 |  | EITALAPSTMK | Oxidation | P;S | Other |  |  |  | + | DP |
| A6NL76 | Actin, alpha cardiac muscle 1 | ACTA1 |  | IIAPPER | Peptide:I.IAPPER | I | Other | + |  |  |  | DP |
| A6NL76 | Actin, alpha cardiac muscle 1 | ACTA1 |  | IIAPPER | tri-Methylation | cterm;E;P;R | Other |  |  |  | + | DP |
| A6NL76 | Actin, alpha cardiac muscle 1 | ACTA1 |  | IIAPPER | Peptide:I.KIIAPPER | nterm | Other | + | + | + | + | DP |
| A8MUF7 | Hemoglobin subunit beta | HBB |  | LLVVYPWTQR | Peptide:LLVVYPWTQRF.F | cterm;Q;R;T | Other |  | + |  |  | DP |
| B0YJC4 | Vimentin | VIM |  | LQEEMLQR | Reduction | L;M;Q | Other |  |  |  | + | DP |
| B0YJC4 | Vimentin | VIM |  | LQEEMLQR | Diffuse:2 | nterm | Other | + |  |  |  | DP |
| B4DEB1 | Histone H3 | H3F3A |  | EIAQDFK | Methylation | cterm;F;K | Other |  |  |  | + | DP |
| B4DEB1 | Histone H3 | H3F3A |  | EIAQDFK | Peptide:V.REIAQDFK | nterm | Other |  |  |  | + | DP |
| B4DNK4 | Pyruvate kinase | PKM |  | LDIDSPPITAR | Peptide:L.DIDSPPITAR | I | Other | + |  |  |  | DP |
| B4DNK4 | Pyruvate kinase | PKM |  | NTGIICTIGPASR | Diffuse:2 | I | Other | + |  |  |  | DP |
| B4DV12 | 40S ribosomal protein S27a | RPS27A |  | ESTLHLVLR | Diffuse:2 | nterm | Other |  |  |  | + | DP |
| B8ZZL8 | 10 kDa heat shock protein, mitochondrial | HSPE1 |  | FLPLFDR | K | F;L;P | Other |  |  |  | + | DP |
| C9J7T9 | Calmodulin-like protein 3 | CALM1 |  | ELGTVMR | Reduction | M | Other | + | + |  | + | DP |
| C9JCF5 | Kaliocin-1 | LTF |  | ADAVTLDGGFIYEAGLAPYK | Peptide:V.TLDGGFIYEAGLAPYK | nterm | Other |  | + |  | + | DP |
| C9JCF5 | Kaliocin-1 | LTF |  | GGSFQLNELQGLK | Peptide:K.KGGSFQLNELQGLK | nterm | Other |  |  |  | + | DP |
| C9JCF5 | Kaliocin-1 | LTF |  | KGGSFQLNELQGLK | Deamidation | E | Other |  | + |  |  | DP |
| C9JCF5 | Kaliocin-1 | LTF |  | KGGSFQLNELQGLK | Acetaldehyde | nterm | Other |  | + |  | + | DP |
| C9JCF5 | Kaliocin-1 | LTF |  | LRPVAAEVYGTER | Loss of water | E;G;T | Other |  |  |  | + | DP |
| C9JCF5 | Kaliocin-1 | LTF |  | LRPVAAEVYGTER | Diffuse:2 | E;V;Y | Other |  |  | + |  | DP |
| C9JCF5 | Kaliocin-1 | LTF |  | LRPVAAEVYGTER | Acetaldehyde | nterm | Other |  | + | + | + | DP |
| C9JCF5 | Kaliocin-1 | LTF |  | LRPVAAEVYGTER | Carbamidomethyl | R | Other |  | + | + | + | DP |
| C9JCF5 | Kaliocin-1 | LTF |  | RDSPIQCIQAIAENR | Diffuse:4 | C;I | Other |  |  | + |  | DP |
| C9JCF5 | Kaliocin-1 | LTF |  | SCHTGLR | Sulfide | nterm | Other |  |  |  | + | DP |
| C9JCF5 | Kaliocin-1 | LTF |  | SCHTGLR | D | G;L | Other |  |  |  | + | DP |
| C9JCF5 | Kaliocin-1 | LTF |  | TAGWNVPIGTLR | Peptide:T.AGWNVPIGTLR | nterm | Other |  | + |  |  | DP |
| C9JCF5 | Kaliocin-1 | LTF |  | TAGWNVPIGTLR | Diffuse:2 | N;W | Other |  | + |  |  | DP |
| C9JCF5 | Kaliocin-1 | LTF |  | TAGWNVPIGTLR | Acetaldehyde | nterm | Other |  | + |  | + | DP |
| C9JCF5 | Kaliocin-1 | LTF |  | THYYAVAVVK | Acetaldehyde | nterm | Other |  |  |  | + | DP |
| C9JCF5 | Kaliocin-1 | LTF |  | THYYAVAVVK | Peptide:THYYAVAVVKKG.G | cterm;K | Other |  |  |  | + | DP |
| C9JQ42 | Glycogenin-1 | GYG1 |  | LVVLATPQVSDSMR | Reduction | cterm;M;R;S | Other |  | + | + | + | DP |
| D6R9A6 | High mobility group protein B2 | HMGB2 |  | KDPNAPK | Comp.:13C-12C | D;N;P | Other |  |  | + |  | DP |
| D6R9A6 | High mobility group protein B2 | HMGB2 |  | SEHPGLSIGDTAK | Peptide:SEHPGLSIGDTA.K | A;cterm;K | Other |  | + | + | + | DP |
| D6RA82 | Annexin | ANXA3 |  | MLISILTER | Reduction | nterm | Other |  |  |  | + | DP |
| D6RFF0 | La-related protein 7 | LARP7 |  | KEDNIQAKEENMDTSNTSISK | E | nterm | Other |  |  | + |  | DP |
| D6RFF0 | La-related protein 7 | LARP7 |  | KEDNIQAKEENMDTSNTSISK | Loss of water | nterm | Other |  | + |  | + | DP |
| D6RFF0 | La-related protein 7 | LARP7 |  | KEDNIQAKEENMDTSNTSISK | Comp.:12C-13C | A;D;E;K;M;N;Q;S;T | Other |  | + | + |  | DP |
| D6RFF0 | La-related protein 7 | LARP7 |  | KEDNIQAKEENMDTSNTSISK | Deamidation | N | Other | + | + | + | + | DP |
| D6RFF0 | La-related protein 7 | LARP7 |  | KEDNIQAKEENMDTSNTSISK | Comp.:13C-12C | D;N | Other | + |  | + |  | DP |
| D6RFF0 | La-related protein 7 | LARP7 |  | KEDNIQAKEENMDTSNTSISK | Sulfide | A;D;E;I;K;M;N;Q;S;T | Other |  | + |  |  | DP |
| D6RGY2 | Calnexin | CANX |  | KPEDWDERPK | Diffuse:2 | nterm | Other |  |  | + |  | DP |
| E5RG95 | Alpha-enolase | ENO1 |  | FGANAILGVSLAVCK | Comp.:H4C3 | nterm | Other | + |  |  |  | DP |
| E5RGE1 | 14-3-3 protein zeta/delta | YWHAZ |  | SVTEQGAELSNEER | Peptide:V.TEQGAELSNEER | nterm | Other | + |  |  |  | DP |
| E7EMB3 | Calmodulin-like protein 3 | CALM1 |  | EAFSLFDK | Diffuse:2 | nterm | Other |  |  |  | + | DP |
| E7EMB3 | Calmodulin-like protein 3 | CALM1 |  | EAFSLFDK | Diffuse:2 | nterm | Other |  |  |  | + | DP |
| E7EQB2 | Kaliocin-1 | LTF |  | DGAGDVAFIR | Loss of water | nterm | Other |  |  |  | + | DP |
| E7EQB2 | Kaliocin-1 | LTF |  | DGAGDVAFIR | Acetaldehyde | nterm | Other |  |  |  | + | DP |
| E7EQB2 | Kaliocin-1 | LTF |  | DLLFKDSAIGFSR | Comp.:12C-13C | F | Other |  | + |  |  | DP |
| E7EQB2 | Kaliocin-1 | LTF |  | DLLFKDSAIGFSR | GlyGly | K | Other |  | + |  | + | DP |
| E7EQB2 | Kaliocin-1 | LTF |  | DSAIGFSR | Comp.:13C-12C | A;I | Other |  | + |  |  | DP |
| E7EQB2 | Kaliocin-1 | LTF |  | DVTVLQNTDGNNNEAWAK | Peptide:V.TVLQNTDGNNNEAWAK | nterm | Other |  | + | + | + | DP |
| E7EQB2 | Kaliocin-1 | LTF |  | DVTVLQNTDGNNNEAWAK | Comp.:-H4O2 | E | Other |  | + | + | + | DP |
| E7EQB2 | Kaliocin-1 | LTF |  | DVTVLQNTDGNNNEAWAK | Comp.:12C-13C | A;E;G;N;W | Other |  |  | + |  | DP |
| E7EQB2 | Kaliocin-1 | LTF |  | DVTVLQNTDGNNNEAWAK | Deamidation | N | Other |  | + |  | + | DP |
| E7EQB2 | Kaliocin-1 | LTF |  | EDAIWNLLR | Diffuse:2 | nterm | Other |  | + |  |  | DP |
| E7EQB2 | Kaliocin-1 | LTF |  | EDAIWNLLR | Trp->Kynurenin | W | Other |  |  |  | + | DP |
| E7EQB2 | Kaliocin-1 | LTF |  | EDAIWNLLR | Oxidation | W | Other |  | + |  | + | DP |
| E7EQB2 | Kaliocin-1 | LTF |  | EDAIWNLLR | Comp.:O2-C | W | Other |  |  |  | + | DP |
| E7EQB2 | Kaliocin-1 | LTF |  | EDAIWNLLR | di-Oxidation | W | Other |  | + |  | + | DP |
| E7EQB2 | Kaliocin-1 | LTF |  | EDAIWNLLR | tri-Methylation | nterm | Other |  | + |  | + | DP |
| E7EQB2 | Kaliocin-1 | LTF |  | ESTVFEDLSDEAER | Peptide:F.EDLSDEAER | nterm | Other |  | + |  |  | DP |
| E7EQB2 | Kaliocin-1 | LTF |  | ESTVFEDLSDEAER | Peptide:T.VFEDLSDEAER | nterm | Other |  | + |  |  | DP |
| E7EQB2 | Kaliocin-1 | LTF |  | FDEYFSQSCAPGSDPR | Comp.:-H5C2NOS | A;C | Other |  |  |  | + | DP |
| E7EQB2 | Kaliocin-1 | LTF |  | FDEYFSQSCAPGSDPR | Comp.:-CH4O | A;C;S | Other |  | + |  |  | DP |
| E7EQB2 | Kaliocin-1 | LTF |  | FQLFGSPSGQK | Diffuse:2 | F;L | Other |  | + |  |  | DP |
| E7EQB2 | Kaliocin-1 | LTF |  | FQLFGSPSGQK | Deamidation | nterm | Other |  | + | + | + | DP |
| E7EQB2 | Kaliocin-1 | LTF |  | GEADAMSLDGGYVYTAGK | Oxidation | nterm | Other |  | + |  |  | DP |
| E7EQB2 | Kaliocin-1 | LTF |  | GQFPNLCRLCAGTGENK | SA | A;C;E;G;L;R;T | Other |  |  | + | + | DP |
| E7EQB2 | Kaliocin-1 | LTF |  | GQFPNLCRLCAGTGENK | T | A;C;E;G;L;N;R;T | Other |  |  | + |  | DP |
| E7EQB2 | Kaliocin-1 | LTF |  | GQFPNLCRLCAGTGENK | Carbamidomethyl | A;C;E;G;L;N;P;R;T | Other |  |  |  | + | DP |
| E7EQB2 | Kaliocin-1 | LTF |  | IDSGLYLGSGYFTAIQNLR | Peptide:Y.LGSGYFTAIQNLR | nterm | Other |  | + |  | + | DP |
| E7EQB2 | Kaliocin-1 | LTF |  | LADFALLCLDGK | Comp.:-H5C2NOS | C | Other |  | + |  | + | DP |
| E7EQB2 | Kaliocin-1 | LTF |  | LADFALLCLDGK | G | C | Other |  | + |  |  | DP |
| E7EQB2 | Kaliocin-1 | LTF |  | LADFALLCLDGK | Comp.:-CH4O | C;L | Other |  | + |  |  | DP |
| E7EQB2 | Kaliocin-1 | LTF |  | LADFALLCLDGK | Sulfide | C | Other |  | + |  |  | DP |
| E7EQB2 | Kaliocin-1 | LTF |  | NLLFNDNTECLAR | Comp.:-H5C2NOS | C | Other |  | + | + | + | DP |
| E7EQB2 | Kaliocin-1 | LTF |  | NLLFNDNTECLAR | G | A;C;cterm;L;R | Other |  | + |  |  | DP |
| E7EQB2 | Kaliocin-1 | LTF |  | NLLFNDNTECLAR | Comp.:-CH4O | A;C;cterm;L;R | Other |  | + |  | + | DP |
| E7EQB2 | Kaliocin-1 | LTF |  | NLLFNDNTECLAR | Sulfide | C | Other |  | + |  | + | DP |
| E7EQB2 | Kaliocin-1 | LTF |  | QVLLHQQAK | Peptide:V.LLHQQAK | nterm | Other |  | + |  |  | DP |
| E7EQB2 | Kaliocin-1 | LTF |  | QVLLHQQAK | Loss of ammonia | nterm | Other |  | + |  | + | DP |
| E7EQB2 | Kaliocin-1 | LTF |  | RSDTSLTWNSVK | Deamidation | N | Other |  | + |  | + | DP |
| E7EQB2 | Kaliocin-1 | LTF |  | SCHLAMAPNHAVVSR | Comp.:-H5C2NOS | C | Other |  | + |  | + | DP |
| E7EQB2 | Kaliocin-1 | LTF |  | SCHLAMAPNHAVVSR | Comp.:-CH4O | nterm | Other |  | + |  | + | DP |
| E7EQB2 | Kaliocin-1 | LTF |  | SCHLAMAPNHAVVSR | Comp.:O2-C2H3N | nterm | Other |  | + |  | + | DP |
| E7EQB2 | Kaliocin-1 | LTF |  | SDTSLTWNSVK | Comp.:12C-13C | N | Other |  |  |  | + | DP |
| E7EQB2 | Kaliocin-1 | LTF |  | SDTSLTWNSVK | Deamidation | N | Other |  | + | + | + | DP |
| E7EQB2 | Kaliocin-1 | LTF |  | SDTSLTWNSVK | Peptide:R.RSDTSLTWNSVK | nterm | Other |  |  |  | + | DP |
| E7EQB2 | Kaliocin-1 | LTF |  | SQQSSDPDPNCVDRPVEGYLAVAVVR | Comp.:-H5C2NOS | A;C;cterm;D;E;G;L;N;P;R;V;Y | Other |  | + |  |  | DP |
| E7EQB2 | Kaliocin-1 | LTF |  | SQQSSDPDPNCVDRPVEGYLAVAVVR | G | A;C;cterm;D;E;G;L;N;P;R;V;Y | Other |  | + |  |  | DP |
| E7EQB2 | Kaliocin-1 | LTF |  | SQQSSDPDPNCVDRPVEGYLAVAVVR | Sulfide | A;C;cterm;D;E;G;L;N;P;R;V;Y | Other |  | + |  | + | DP |
| E7EQB2 | Kaliocin-1 | LTF |  | SVNGKEDAIWNLLR | Loss of ammonia | D;E;G;K;N | Other |  |  |  | + | DP |
| E7EQB2 | Kaliocin-1 | LTF |  | SVNGKEDAIWNLLR | Comp.:12C-13C | G;K;N | Other |  | + |  |  | DP |
| E7EQB2 | Kaliocin-1 | LTF |  | SVNGKEDAIWNLLR | Deamidation | G;K;N | Other |  | + |  |  | DP |
| E7EQB2 | Kaliocin-1 | LTF |  | VPSHAVVAR | Peptide:V.PSHAVVAR | nterm | Other |  | + | + | + | DP |
| E7EQB2 | Kaliocin-1 | LTF |  | YLGPQYVAGITNLK | Acetaldehyde | nterm | Other |  | + |  | + | DP |
| E7EQB2 | Kaliocin-1 | LTF |  | YLGPQYVAGITNLKK | Diffuse:2 | nterm | Other |  |  |  | + | DP |
| E7EUT5 | Glyceraldehyde-3-phosphate dehydrogenase | GAPDH |  | LISWYDNEFGYSNR | Oxidation | F | Other | + | + |  |  | DP |
| E7EUT5 | Glyceraldehyde-3-phosphate dehydrogenase | GAPDH |  | VIPELNGK | Loss of ammonia | cterm;K | Other | + | + |  |  | DP |
| E7EUT5 | Glyceraldehyde-3-phosphate dehydrogenase | GAPDH |  | VIPELNGK | Deamidation | N | Other | + | + | + | + | DP |
| E7EUT5 | Glyceraldehyde-3-phosphate dehydrogenase | GAPDH |  | VPTANVSVVDLTCR | Acetaldehyde | A;N;V | Other | + |  |  |  | DP |
| E9PEW8 | Hemoglobin subunit beta | HBB |  | VLGAFSDGLAHLDNLK | Peptide:D.GLAHLDNLK | nterm | Other |  | + |  | + | DP |
| E9PEW8 | Hemoglobin subunit beta | HBB |  | VLGAFSDGLAHLDNLK | Peptide:S.DGLAHLDNLK | nterm | Other | + | + |  | + | DP |
| E9PEW8 | Hemoglobin subunit beta | HBB |  | VLGAFSDGLAHLDNLK | Peptide:F.SDGLAHLDNLK | nterm | Other | + | + | + | + | DP |
| E9PEW8 | Hemoglobin subunit beta | HBB |  | VLGAFSDGLAHLDNLK | Peptide:VLGAFSDGLAHL.D | cterm;D;H;K;L;N | Other |  | + |  |  | DP |
| E9PEW8 | Hemoglobin subunit beta | HBB |  | VLGAFSDGLAHLDNLK | Peptide:L.GAFSDGLAHLDNLK | nterm | Other |  | + |  | + | DP |
| E9PEW8 | Hemoglobin subunit beta | HBB |  | VLGAFSDGLAHLDNLK | Peptide:VLGAFSDGLAHLDNL.K | cterm;D;H;K;L;N | Other |  | + |  |  | DP |
| E9PEW8 | Hemoglobin subunit beta | HBB |  | VLGAFSDGLAHLDNLK | Peptide:V.LGAFSDGLAHLDNLK | nterm | Other |  | + | + |  | DP |
| E9PEW8 | Hemoglobin subunit beta | HBB |  | VLGAFSDGLAHLDNLK | Peptide:VLGAFSDGLAHLDNLKG.T | H | Other | + | + |  | + | DP |
| E9PEW8 | Hemoglobin subunit beta | HBB |  | VLGAFSDGLAHLDNLK | Peptide:VLGAFSDGLAHLDNLKGTFA.T | cterm;D;K;L;N | Other |  | + |  |  | DP |
| E9PK25 | Cofilin-1 | CFL1 |  | MLPDKDCR | Reduction | nterm | Other |  | + |  |  | DP |
| E9PKU4 | 60S ribosomal protein L8 | RPL8 |  | IDKPILK | Acetaldehyde | D | Other | + |  |  |  | DP |
| E9PP15 | Low-density lipoprotein receptor-related protein 8 | LRP8 |  | SMNFDNPVYRK | Loss of ammonia | nterm | Other |  |  |  | + | DP |
| E9PP15 | Low-density lipoprotein receptor-related protein 8 | LRP8 |  | SMNFDNPVYRK | Reduction | nterm | Other |  | + | + | + | DP |
| E9PP15 | Low-density lipoprotein receptor-related protein 8 | LRP8 |  | SMNFDNPVYRK | Deamidation | N | Other | + |  | + |  | DP |
| F5H2R5 | Rho GDP-dissociation inhibitor 2 | ARHGDIB |  | APEPHVEEDDDDELDSK | Peptide:APEPHVEEDDDDELDSKL.N | cterm;K | Other | + |  |  |  | DP |
| F5H6T1 | Actin-related protein 2 | ACTR2 |  | RLDIAGR | Deamidation | G | Other | + | + | + | + | DP |
| F5H6T1 | Actin-related protein 2 | ACTR2 |  | RLDIAGR | Comp.:O-H | A;D;I;L | Other | + | + | + |  | DP |
| F5H6T1 | Actin-related protein 2 | ACTR2 |  | RLDIAGR | Acetaldehyde | nterm | Other |  |  |  | + | DP |
| F5H6T1 | Actin-related protein 2 | ACTR2 |  | RLDIAGR | Peptide:RLDIAGRD.I | cterm;R | Other | + |  | + | + | DP |
| F8VQQ4 | Tubulin alpha-1A chain | TUBA1A |  | LIGQIVSSITASLR | Comp.:H2CO | G;Q | Other | + |  |  |  | DP |
| F8W6P5 | Hemoglobin subunit beta | HBB |  | FFESFGDLSTPDAVMGNPK | Oxidation | A;cterm;D;G;K;M;N;P;V | Other |  | + |  |  | DP |
| F8W6P5 | Hemoglobin subunit beta | HBB |  | VNVDEVGGEALGR | Peptide:V.NVDEVGGEALGR | V | Other |  |  |  | + | DP |
| F8W8C1 | E3 ubiquitin-protein ligase TRIM9 | TRIM67 |  | AWAMYVDNNR | Comp.:-H4O2 | cterm;N;R | Other | + | + | + | + | DP |
| F8W8C1 | E3 ubiquitin-protein ligase TRIM9 | TRIM67 |  | AWAMYVDNNR | Deamidation | A;M;V;Y | Other |  | + | + |  | DP |
| F8W8C1 | E3 ubiquitin-protein ligase TRIM9 | TRIM67 |  | AWAMYVDNNR | Acetaldehyde | cterm;N;R | Other | + | + | + | + | DP |
| F8W8C1 | E3 ubiquitin-protein ligase TRIM9 | TRIM67 |  | AWAMYVDNNR | tri-Methylation | A;M;V;Y | Other |  |  |  | + | DP |
| H0Y5C6 | Filamin-A | FLNA |  | VSGQGLHEGHTFEPAEFIIDTR | Peptide:V.SGQGLHEGHTFEPAEFIIDTR | nterm | Other |  |  | + |  | DP |
| H0YJ11 | Alpha-actinin-1 | ACTN1 |  | ISIEMHGTLEDQLSHLR | Peptide:I.SIEMHGTLEDQLSHLR | E;G;H;I;L;M;T | Other |  |  |  | + | DP |
| H0YJ11 | Alpha-actinin-1 | ACTN1 |  | RDQALTEEHAR | Diffuse:2 | L;T | Other | + |  |  |  | DP |
| H0YJ11 | Alpha-actinin-1 | ACTN1 |  | RDQALTEEHAR | Diffuse:4 | L;T | Other |  |  |  | + | DP |
| H3BQ34 | Pyruvate kinase | PKM |  | TATESFASDPILYRPVAVALDTK | Formylation | nterm | Other |  | + |  |  | DP |
| I3L1U9 | Actin, alpha cardiac muscle 1 | ACTA1 |  | DLTDYLMK | Reduction | cterm;K;M | Other |  | + |  |  | DP |
| I3L3D5 | Profilin-1 | PFN1 |  | TFVNITPAEVGVLVGK | Comp.:C | nterm | Other | + | + |  |  | DP |
| I3L4N8 | Actin, cytoplasmic 1 | ACTB |  | LCYVALDFEQEMATAASSSSLEK | Comp.:C | nterm | Other | + | + |  |  | DP |
| J3KNB4 | Antibacterial protein FALL-39 | CAMP |  | FALLGDFFR | Peptide:A.LLGDFFR | nterm | Other |  | + |  |  | DP |
| J3KNB4 | Antibacterial protein FALL-39 | CAMP |  | SSDANLYR | Comp.:C | D;S | Other |  |  | + |  | DP |
| J3KSZ8 | Male-specific lethal 1 homolog | MSL1 |  | LELDEKR | Comp.:C | D;E;K | Other | + |  |  | + | DP |
| J3QSF7 | 84 kDa myeloperoxidase | MPO |  | QNQIAVDEIR | Loss of ammonia | nterm | Other |  | + |  | + | DP |
| J3QSF7 | 84 kDa myeloperoxidase | MPO |  | VFFASWR | Diffuse:2 | nterm | Other |  |  | + |  | DP |
| J3QSF7 | 84 kDa myeloperoxidase | MPO |  | VFFASWR | Trp->Kynurenin | W | Other |  | + |  |  | DP |
| J3QSF7 | 84 kDa myeloperoxidase | MPO |  | VFFASWR | Oxidation | A;S;W | Other |  |  |  | + | DP |
| J3QSF7 | 84 kDa myeloperoxidase | MPO |  | VFFASWR | Acetaldehyde | nterm | Other |  | + |  | + | DP |
| J3QSF7 | 84 kDa myeloperoxidase | MPO |  | VFFASWR | di-Oxidation | S;W | Other |  | + |  |  | DP |
| J3QSF7 | 84 kDa myeloperoxidase | MPO |  | VFFASWR | tri-Oxidation | S;W | Other |  | + |  |  | DP |
| J3QSF7 | 84 kDa myeloperoxidase | MPO |  | VVLEGGIDPILR | tri-Methylation | E | Other |  |  |  | + | DP |
| J3QSF7 | 84 kDa myeloperoxidase | MPO |  | YQPMEPNPR | Reduction | M | Other |  |  |  | + | DP |
| J3QSF7 | 84 kDa myeloperoxidase | EPX |  | GLMATPAK | Reduction | M | Other |  |  |  | + | DP |
| J3QTJ6 | Fibrous sheath-interacting protein 2 | FSIP2 |  | NNSVPLCNK | Sulfide | C;cterm;K;L;N;P;V | Other |  | + |  |  | DP |
| K7EJ44 | Profilin-1 | PFN1 |  | EGVHGGLINK | IN | I;L;N | Other | + |  |  |  | DP |
| K7EJ44 | Profilin-1 | PFN1 |  | EGVHGGLINK | Acetaldehyde | nterm | Other |  |  |  | + | DP |
| K7EJB9 | Calreticulin | CALR |  | IDDPTDSKPEDWDKPEHIPDPDAK | Peptide:I.DDPTDSKPEDWDKPEHIPDPDAK | nterm | Other | + | + | + |  | DP |
| K7EM49 | 6-phosphogluconate dehydrogenase, decarboxylating | PGD |  | VDDFLANEAK | ID | A;cterm;E;K;N | Other | + |  |  |  | DP |
| K7EM90 | Alpha-enolase | ENO1 |  | KLNVTEQEK | Peptide:K.LNVTEQEK | nterm | Other | + | + |  |  | DP |
| M0QX14 | Tubulin beta-4A chain | TUBB4A |  | FWEVISDEHGIDPTGTYHGDSDLQLER | Comp.:-H4O2 | cterm;D;E;G;H;I;L;P;Q;R;S;T;V;Y | Other | + |  |  |  | DP |
| M0QYM2 | Carcinoembryonic antigen-related cell adhesion molecule 6 | CEACAM6 |  | EVLLLAHNLPQNR | Loss of water | nterm | Other |  | + |  |  | DP |
| O00254-2 | Proteinase-activated receptor 3 | F2RL2 |  | MENDTNNLAK | Reduction | nterm | Other | + |  |  |  | DP |
| O00254-2 | Proteinase-activated receptor 3 | F2RL2 |  | MENDTNNLAK | Deamidation | A;D;L;N;T | Other | + |  |  |  | DP |
| O14782 | Kinesin-like protein KIF3C | KIF3C |  | SWCQSPQR | Sulfide | nterm | Other | + | + | + | + | DP |
| O14862 | Interferon-inducible protein AIM2 | AIM2 |  | RLQEEKEK | Reduction | E;K;L;Q | Other | + |  |  |  | DP |
| O15541 | RING finger protein 113A | RNF113A |  | DAQAIFER | V | nterm | Other |  |  | + | + | DP |
| O15541 | RING finger protein 113A | RNF113A |  | DAQAIFER | G | nterm | Other |  |  | + | + | DP |
| O60814 | Histone H2B | H2BFS |  | HAVSEGTK | Diffuse:2 | S;V | Other |  |  | + |  | DP |
| O60814 | Histone H2B | H2BFS |  | HAVSEGTK | Comp.:C | nterm | Other | + | + | + | + | DP |
| O60814 | Histone H2B | H2BFS |  | HAVSEGTK | Acetaldehyde | nterm | Other | + | + | + | + | DP |
| O60814 | Histone H2B | H2BFS |  | HAVSEGTK | tri-Methylation | E | Other | + | + | + | + | DP |
| O60814 | Histone H2B | H2BFS |  | HAVSEGTK | Peptide:A.KHAVSEGTK | nterm | Other | + | + | + | + | DP |
| O60814 | Histone H2B | H2BFS |  | LLLPGELAK | Peptide:L.LLPGELAK | L | Other | + |  |  |  | DP |
| O60814 | Histone H2B | H2BFS |  | QVHPDTGISSK | Peptide:QVHPDTGI.S | nterm | Other | + | + |  | + | DP |
| O60814 | Histone H2B | H2BFS |  | QVHPDTGISSK | Peptide:V.HPDTGISSK | nterm | Other | + | + | + | + | DP |
| O60814 | Histone H2B | H2BFS |  | QVHPDTGISSK | Peptide:QVHPDTGIS.S | nterm | Other | + | + | + | + | DP |
| O60814 | Histone H2B | H2BFS |  | QVHPDTGISSK | IG | nterm | Other | + | + | + | + | DP |
| O60814 | Histone H2B | H2BFS |  | QVHPDTGISSK | Peptide:Q.VHPDTGISSK | nterm | Other |  | + | + |  | DP |
| O60814 | Histone H2B | H2BFS |  | QVHPDTGISSK | Peptide:Q.VHPDTGISSK | nterm | Other | + | + | + | + | DP |
| O60814 | Histone H2B | H2BFS |  | QVHPDTGISSK | I | nterm | Other | + | + | + | + | DP |
| O60814 | Histone H2B | H2BFS |  | QVHPDTGISSK | A | nterm | Other | + |  | + | + | DP |
| O60814 | Histone H2B | H2BFS |  | QVHPDTGISSK | Loss of water | nterm | Other | + | + | + | + | DP |
| O60814 | Histone H2B | H2BFS |  | QVHPDTGISSK | Loss of ammonia | nterm | Other | + | + | + | + | DP |
| O60814 | Histone H2B | H2BFS |  | QVHPDTGISSK | Comp.:12C-13C | nterm | Other |  |  | + |  | DP |
| O60814 | Histone H2B | H2BFS |  | QVHPDTGISSK | Deamidation | nterm | Other | + | + | + | + | DP |
| O60814 | Histone H2B | H2BFS |  | QVHPDTGISSK | Diffuse:2 | H;P | Other |  |  | + |  | DP |
| O60814 | Histone H2B | H2BFS |  | QVHPDTGISSK | Acetaldehyde | nterm | Other | + | + | + | + | DP |
| O60814 | Histone H2B | H2BFS |  | QVHPDTGISSK | Comp.:C2O | nterm | Other | + | + | + | + | DP |
| O60814 | Histone H2B | H2BFS |  | QVHPDTGISSK | tri-Methylation | cterm;K;S | Other | + |  |  |  | DP |
| O60814 | Histone H2B | H2BFS |  | QVHPDTGISSK | GlyGly | nterm | Other |  | + |  |  | DP |
| O60814 | Histone H2B | H2BFS |  | QVHPDTGISSK | K | S | Other | + | + | + | + | DP |
| O60814 | Histone H2B | H2BFS |  | QVHPDTGISSK | Peptide:L.KQVHPDTGISSK | nterm | Other | + | + | + | + | DP |
| O60814 | Histone H2B | H2BFS |  | QVHPDTGISSK | Peptide:V.LKQVHPDTGISSK | nterm | Other |  | + |  | + | DP |
| O60814 | Histone H2B | H2BFS |  | AMGIMNSFVNDIFER | Peptide:S.FVNDIFER | nterm | Other | + | + | + | + | DP |
| O60814 | Histone H2B | H2BFS |  | AMGIMNSFVNDIFER | Peptide:S.FVNDIFER | nterm | Other | + | + |  |  | DP |
| O60814 | Histone H2B | H2BFS |  | AMGIMNSFVNDIFER | Peptide:S.FVNDIFER | nterm | Other | + |  | + | + | DP |
| O60814 | Histone H2B | H2BFS |  | AMGIMNSFVNDIFER | Peptide:N.SFVNDIFER | nterm | Other | + | + |  | + | DP |
| O60814 | Histone H2B | H2BFS |  | AMGIMNSFVNDIFER | Peptide:N.SFVNDIFER | nterm | Other |  | + | + | + | DP |
| O60814 | Histone H2B | H2BFS |  | AMGIMNSFVNDIFER | Peptide:N.SFVNDIFER | nterm | Other | + |  | + | + | DP |
| O60814 | Histone H2B | H2BFS |  | AMGIMNSFVNDIFER | Peptide:M.NSFVNDIFER | nterm | Other | + | + | + | + | DP |
| O60814 | Histone H2B | H2BFS |  | AMGIMNSFVNDIFER | Peptide:M.NSFVNDIFER | nterm | Other | + | + |  | + | DP |
| O60814 | Histone H2B | H2BFS |  | AMGIMNSFVNDIFER | Peptide:M.NSFVNDIFER | nterm | Other |  |  | + | + | DP |
| O60814 | Histone H2B | H2BFS |  | AMGIMNSFVNDIFER | Peptide:I.MNSFVNDIFER | nterm | Other | + | + | + |  | DP |
| O60814 | Histone H2B | H2BFS |  | AMGIMNSFVNDIFER | Peptide:I.MNSFVNDIFER | nterm | Other | + | + | + | + | DP |
| O60814 | Histone H2B | H2BFS |  | AMGIMNSFVNDIFER | Peptide:G.IMNSFVNDIFER | nterm | Other |  |  |  | + | DP |
| O60814 | Histone H2B | H2BFS |  | AMGIMNSFVNDIFER | Peptide:G.IMNSFVNDIFER | nterm | Other |  | + |  | + | DP |
| O60814 | Histone H2B | H2BFS |  | AMGIMNSFVNDIFER | Peptide:M.GIMNSFVNDIFER | nterm | Other |  | + |  |  | DP |
| O60814 | Histone H2B | H2BFS |  | AMGIMNSFVNDIFER | Peptide:M.GIMNSFVNDIFER | nterm | Other | + | + |  | + | DP |
| O60814 | Histone H2B | H2BFS |  | AMGIMNSFVNDIFER | Loss of ammonia | D;F;I;N;V | Other | + | + |  |  | DP |
| O60814 | Histone H2B | H2BFS |  | AMGIMNSFVNDIFER | Reduction | M | Other | + | + |  | + | DP |
| O60814 | Histone H2B | H2BFS |  | AMGIMNSFVNDIFER | Comp.:ON2-C2H3S | G;I | Other |  | + |  |  | DP |
| O60814 | Histone H2B | H2BFS |  | AMGIMNSFVNDIFER | Diffuse:2 | I | Other |  | + |  |  | DP |
| O60814 | Histone H2B | H2BFS |  | AMGIMNSFVNDIFER | Diffuse:2 | I;M | Other |  |  |  | + | DP |
| O60814 | Histone H2B | H2BFS |  | AMGIMNSFVNDIFER | Diffuse:3 | M;N;S | Other |  |  | + |  | DP |
| O60814 | Histone H2B | H2BFS |  | AMGIMNSFVNDIFER | Comp.:HN | M;N | Other |  | + |  |  | DP |
| O60814 | Histone H2B | H2BFS |  | AMGIMNSFVNDIFER | Oxidation | nterm | Other |  |  | + |  | DP |
| O60814 | Histone H2B | H2BFS |  | AMGIMNSFVNDIFER | di-Oxidation | nterm | Other | + |  |  |  | DP |
| P00338 | L-lactate dehydrogenase A chain | LDHA |  | TLHPDLGTDKDK | Peptide:TLHPDLGTDKDKEQW.K | cterm;D;K | Other |  | + |  | + | DP |
| P00558 | Phosphoglycerate kinase 1 | PGK1 |  | ITLPVDFVTADK | Peptide:I.TLPVDFVTADK | L | Other |  | + |  |  | DP |
| P02042 | Hemoglobin subunit beta | HBB |  | VVAGVANALAHKYH | Peptide:VVAGVANALAHK.Y | cterm;H | Other |  | + |  |  | DP |
| P02671 | Fibrinogen alpha chain | FGA |  | ESSSHHPGIAEFPSR | Peptide:S.SHHPGIAEFPSR | nterm | Other |  |  | + |  | DP |
| P02763 | Alpha-1-acid glycoprotein 1 | ORM1 |  | NWGLSVYADKPETTK | Diffuse:2 | L | Other |  |  |  | + | DP |
| P04083 | Annexin A1 | ANXA1 |  | SEDFGVNEDLADSDAR | Peptide:V.NEDLADSDAR | nterm | Other | + |  | + | + | DP |
| P04083 | Annexin A1 | ANXA1 |  | SEDFGVNEDLADSDAR | Peptide:F.GVNEDLADSDAR | nterm | Other |  |  | + | + | DP |
| P04083 | Annexin | ANXA1 |  | DITSDTSGDFR | Diffuse:2 | S;T | Other |  | + |  |  | DP |
| P05109 | Protein S100-A8 | S100A8 |  | ALNSIIDVYHK | Deamidation | N;S | Other |  | + |  | + | DP |
| P05109 | Protein S100-A8 | S100A8 |  | GNFHAVYR | Peptide:N.FHAVYR | nterm | Other |  |  | + | + | DP |
| P05109 | Protein S100-A8 | S100A8 |  | GNFHAVYR | Loss of ammonia | nterm | Other |  |  |  | + | DP |
| P05109 | Protein S100-A8 | S100A8 |  | GNFHAVYR | Diffuse:2 | nterm | Other |  | + |  |  | DP |
| P05109 | Protein S100-A8 | S100A8 |  | KGADVWFK | Peptide:K.GADVWFK | nterm | Other |  | + | + | + | DP |
| P05109 | Protein S100-A8 | S100A8 |  | KGADVWFK | Loss of water | F | Other |  | + |  |  | DP |
| P05109 | Protein S100-A8 | S100A8 |  | KGADVWFK | Acetaldehyde | nterm | Other |  |  |  | + | DP |
| P05109 | Protein S100-A8 | S100A8 |  | KGADVWFK | Formylation | G | Other |  |  | + |  | DP |
| P05109 | Protein S100-A8 | S100A8 |  | LLETECPQYIR | Peptide:L.LETECPQYIR | L | Other | + | + | + | + | DP |
| P05109 | Protein S100-A8 | S100A8 |  | LLETECPQYIR | Sulfide | C | Other |  | + |  | + | DP |
| P05109 | Protein S100-A8 | S100A8 |  | MLTELEK | Comp.:-CH4OS | nterm | Other | + | + |  |  | DP |
| P05109 | Protein S100-A8 | S100A8 |  | MLTELEK | Reduction | nterm | Other | + | + | + | + | DP |
| P05109 | Protein S100-A8 | S100A8 |  | MLTELEK | Comp.:13C-12C | nterm | Other |  | + |  |  | DP |
| P05109 | Protein S100-A8 | S100A8 |  | MLTELEK | Oxidation | nterm | Other |  | + |  |  | DP |
| P05109 | Protein S100-A8 | S100A8 |  | MLTELEK | Comp.:HCN | nterm | Other |  | + |  |  | DP |
| P05109 | Protein S100-A8 | S100A8 |  | MLTELEK | Comp.:H3C2N | nterm | Other | + | + |  |  | DP |
| P05109 | Protein S100-A8 | S100A8 |  | MLTELEK | Peptide:MLTELEKA.L | cterm;K | Other |  | + |  |  | DP |
| P05109 | Protein S100-A8 | S100A8 |  | SHEESHK | K | H | Other |  | + |  |  | DP |
| P05109 | Protein S100-A8 | S100A8 |  | SHEESHKE | Peptide:SHEESHK.E | cterm;E | Other | + |  |  | + | DP |
| P05109 | Protein S100-A8 | S100A8 |  | SHEESHKE | Diffuse:2 | cterm;E;S | Other |  |  | + |  | DP |
| P05109 | Protein S100-A8 | S100A8 |  | SHEESHKE | Peptide:K.KSHEESHKE | nterm | Other |  | + |  |  | DP |
| P05164 | 84 kDa myeloperoxidase | MPO |  | AVSNEIVR | Deamidation | N | Other |  | + |  | + | DP |
| P05164 | 84 kDa myeloperoxidase | MPO |  | AVSNEIVRFPTDQLTPDQER | Peptide:V.RFPTDQLTPDQER | nterm | Other |  | + |  | + | DP |
| P05164 | 84 kDa myeloperoxidase | MPO |  | AVSNEIVRFPTDQLTPDQER | Peptide:I.VRFPTDQLTPDQER | nterm | Other |  |  |  | + | DP |
| P05164 | 84 kDa myeloperoxidase | MPO |  | AVSNEIVRFPTDQLTPDQER | Deamidation | E;N;S | Other |  | + |  |  | DP |
| P05164 | 84 kDa myeloperoxidase | MPO |  | DYLPLVLGPTAMR | Reduction | A;cterm;M;P;R;T | Other |  | + |  |  | DP |
| P05164 | 84 kDa myeloperoxidase | MPO |  | FPTDQLTPDQER | Peptide:T.DQLTPDQER | nterm | Other |  | + |  |  | DP |
| P05164 | 84 kDa myeloperoxidase | MPO |  | FPTDQLTPDQER | Deamidation | E;Q | Other |  |  |  | + | DP |
| P05164 | 84 kDa myeloperoxidase | MPO |  | FPTDQLTPDQER | Comp.:13C-12C | nterm | Other |  |  |  | + | DP |
| P05164 | 84 kDa myeloperoxidase | MPO |  | FPTDQLTPDQER | Diffuse:2 | nterm | Other |  |  |  | + | DP |
| P05164 | 84 kDa myeloperoxidase | MPO |  | FPTDQLTPDQER | Peptide:V.RFPTDQLTPDQER | nterm | Other |  | + |  |  | DP |
| P05164 | 84 kDa myeloperoxidase | MPO |  | FWWENEGVFSMQQR | Trp->Kynurenin | W | Other |  | + |  | + | DP |
| P05164 | 84 kDa myeloperoxidase | MPO |  | FWWENEGVFSMQQR | Oxidation | W | Other |  | + |  | + | DP |
| P05164 | 84 kDa myeloperoxidase | MPO |  | FWWENEGVFSMQQR | Acetaldehyde | nterm | Other |  |  |  | + | DP |
| P05164 | 84 kDa myeloperoxidase | MPO |  | IANVFTNAFR | Peptide:N.VFTNAFR | nterm | Other |  | + |  | + | DP |
| P05164 | 84 kDa myeloperoxidase | MPO |  | IANVFTNAFR | Deamidation | N | Other | + | + | + | + | DP |
| P05164 | 84 kDa myeloperoxidase | MPO |  | IANVFTNAFR | Acetaldehyde | nterm | Other |  | + | + | + | DP |
| P05164 | 84 kDa myeloperoxidase | MPO |  | IGLDLPALNMQR | Peptide:I.GLDLPALNMQR | L | Other |  | + |  |  | DP |
| P05164 | 84 kDa myeloperoxidase | MPO |  | IGLDLPALNMQR | Loss of water | D;L | Other |  |  |  | + | DP |
| P05164 | 84 kDa myeloperoxidase | MPO |  | IGLDLPALNMQR | Reduction | M;Q | Other |  | + | + | + | DP |
| P05164 | 84 kDa myeloperoxidase | MPO |  | IGLDLPALNMQR | Comp.:12C-13C | cterm;M;N;Q;R | Other |  | + |  | + | DP |
| P05164 | 84 kDa myeloperoxidase | MPO |  | IGLDLPALNMQR | Deamidation | N | Other |  | + | + |  | DP |
| P05164 | 84 kDa myeloperoxidase | MPO |  | IGLDLPALNMQR | Oxidation | cterm;M;Q;R | Other |  |  |  | + | DP |
| P05164 | 84 kDa myeloperoxidase | MPO |  | IGLDLPALNMQR | Comp.:O2-HN | cterm;M;N;Q;R | Other |  |  |  | + | DP |
| P05164 | 84 kDa myeloperoxidase | MPO |  | IGLDLPALNMQR | Acetaldehyde | nterm | Other |  | + | + | + | DP |
| P05164 | 84 kDa myeloperoxidase | MPO |  | IGLDLPALNMQR | Comp.:H4C3 | nterm | Other |  |  |  | + | DP |
| P05164 | 84 kDa myeloperoxidase | MPO |  | IVGAMVQIITYR | Oxidation | M | Other |  | + |  |  | DP |
| P05164 | 84 kDa myeloperoxidase | MPO |  | LFEQVMR | Peptide:L.FEQVMR | nterm | Other |  | + |  |  | DP |
| P05164 | 84 kDa myeloperoxidase | MPO |  | LFEQVMR | Reduction | M | Other | + | + | + | + | DP |
| P05164 | 84 kDa myeloperoxidase | MPO |  | LFEQVMR | Diffuse:2 | nterm | Other |  | + |  |  | DP |
| P05164 | 84 kDa myeloperoxidase | MPO |  | LFEQVMR | Oxidation | M | Other | + | + | + | + | DP |
| P05164 | 84 kDa myeloperoxidase | MPO |  | LFEQVMR | Acetaldehyde | F | Other |  | + |  | + | DP |
| P05164 | 84 kDa myeloperoxidase | MPO |  | LFEQVMR | di-Oxidation | M | Other |  |  | + | + | DP |
| P05164 | 84 kDa myeloperoxidase | MPO |  | LFEQVMR | tri-Methylation | E | Other |  |  |  | + | DP |
| P05164 | 84 kDa myeloperoxidase | MPO |  | NGFPVALAR | Loss of ammonia | nterm | Other |  |  |  | + | DP |
| P05164 | 84 kDa myeloperoxidase | MPO |  | NNIFMSNSYPR | Comp.:-H4O2 | I | Other |  | + |  | + | DP |
| P05164 | 84 kDa myeloperoxidase | MPO |  | NNIFMSNSYPR | Comp.:12C-13C | cterm;N;P;R;S;Y | Other |  | + |  |  | DP |
| P05164 | 84 kDa myeloperoxidase | MPO |  | NNIFMSNSYPR | Comp.:13C-12C | F;I | Other |  | + |  |  | DP |
| P05164 | 84 kDa myeloperoxidase | MPO |  | NQINALTSFVDASMVYGSEEPLAR | Peptide:L.TSFVDASMVYGSEEPLAR | nterm | Other |  |  | + |  | DP |
| P05164 | 84 kDa myeloperoxidase | MPO |  | NQINALTSFVDASMVYGSEEPLAR | Peptide:L.TSFVDASMVYGSEEPLAR | nterm | Other |  | + |  | + | DP |
| P05164 | 84 kDa myeloperoxidase | MPO |  | NQINALTSFVDASMVYGSEEPLAR | Peptide:N.ALTSFVDASMVYGSEEPLAR | nterm | Other |  |  |  | + | DP |
| P05164 | 84 kDa myeloperoxidase | MPO |  | QALAQISLPR | Peptide:A.LAQISLPR | A | Other |  |  | + | + | DP |
| P05164 | 84 kDa myeloperoxidase | MPO |  | QALAQISLPR | Acetaldehyde | nterm | Other |  | + |  | + | DP |
| P05164 | 84 kDa myeloperoxidase | MPO |  | SLMFMQWGQLLDHDLDFTPEPAAR | Peptide:Q.WGQLLDHDLDFTPEPAAR | nterm | Other |  | + |  | + | DP |
| P05164 | 84 kDa myeloperoxidase | MPO |  | SLMFMQWGQLLDHDLDFTPEPAAR | Peptide:F.MQWGQLLDHDLDFTPEPAAR | nterm | Other |  | + |  | + | DP |
| P05164 | 84 kDa myeloperoxidase | MPO |  | SLMFMQWGQLLDHDLDFTPEPAAR | Peptide:F.MQWGQLLDHDLDFTPEPAAR | nterm | Other |  |  |  | + | DP |
| P05164 | 84 kDa myeloperoxidase | MPO |  | SLMFMQWGQLLDHDLDFTPEPAAR | Peptide:M.FMQWGQLLDHDLDFTPEPAAR | nterm | Other |  | + |  | + | DP |
| P05164 | 84 kDa myeloperoxidase | MPO |  | SLMFMQWGQLLDHDLDFTPEPAAR | Reduction | nterm | Other |  | + |  |  | DP |
| P05164 | 84 kDa myeloperoxidase | MPO |  | SLMFMQWGQLLDHDLDFTPEPAAR | Trp->Kynurenin | F;M;Q;W | Other |  | + |  |  | DP |
| P05164 | 84 kDa myeloperoxidase | MPO |  | SLMFMQWGQLLDHDLDFTPEPAAR | Oxidation | W | Other |  |  |  | + | DP |
| P05164 | 84 kDa myeloperoxidase | MPO |  | SLMFMQWGQLLDHDLDFTPEPAAR | di-Oxidation | D;F;G;H;L;M;Q;W | Other |  |  |  | + | DP |
| P05164 | 84 kDa myeloperoxidase | MPO |  | SLMFMQWGQLLDHDLDFTPEPAAR | Comp.:H3C2N | D;L | Other |  |  |  | + | DP |
| P05164 | 84 kDa myeloperoxidase | MPO |  | SLMFMQWGQLLDHDLDFTPEPAAR | Peptide:SLMFMQWGQLLDHDLDFTPEPAARA.S | A;cterm;E;F;P;R;T | Other |  | + |  |  | DP |
| P05164 | 84 kDa myeloperoxidase | MPO |  | SSEMPELTSMHTLLLR | Peptide:M.PELTSMHTLLLR | nterm | Other |  | + | + | + | DP |
| P05164 | 84 kDa myeloperoxidase | MPO |  | SSEMPELTSMHTLLLR | Peptide:M.PELTSMHTLLLR | nterm | Other |  | + |  | + | DP |
| P05164 | 84 kDa myeloperoxidase | MPO |  | SSEMPELTSMHTLLLR | Oxidation | M;S | Other |  | + |  | + | DP |
| P05164 | 84 kDa myeloperoxidase | MPO |  | SSEMPELTSMHTLLLR | Comp.:SH3C-NO | M | Other |  | + |  |  | DP |
| P05164 | 84 kDa myeloperoxidase | MPO |  | TITGMCNNR | Comp.:-H5C2NOS | C | Other |  |  |  | + | DP |
| P05164 | 84 kDa myeloperoxidase | MPO |  | TITGMCNNR | Sulfide | C | Other |  |  |  | + | DP |
| P05164 | 84 kDa myeloperoxidase | MPO |  | YGHTLIQPFMFR | Peptide:YGHTLIQPF.M | cterm;R | Other |  | + |  |  | DP |
| P05164 | 84 kDa myeloperoxidase | EPX |  | DHGLPGYNAWR | Comp.:O-NH3 | Y | Other |  | + |  | + | DP |
| P05164 | 84 kDa myeloperoxidase | EPX |  | DHGLPGYNAWR | Deamidation | N | Other |  | + |  |  | DP |
| P05164 | 84 kDa myeloperoxidase | EPX |  | DHGLPGYNAWR | Comp.:13C-12C | nterm | Other | + | + |  |  | DP |
| P05164 | 84 kDa myeloperoxidase | EPX |  | DHGLPGYNAWR | Acetaldehyde | nterm | Other |  | + |  |  | DP |
| P05164 | 84 kDa myeloperoxidase | EPX |  | IPCFLAGDTR | Comp.:-H5C2NOS | C | Other |  | + |  | + | DP |
| P05164 | 84 kDa myeloperoxidase | EPX |  | IPCFLAGDTR | Comp.:-CH4O | C | Other |  | + |  | + | DP |
| P05164 | 84 kDa myeloperoxidase | EPX |  | IPCFLAGDTR | Sulfide | C | Other |  | + |  | + | DP |
| P05204 | Non-histone chromosomal protein HMG-17 | HMGN2 |  | EGNNPAENGDAK | Deamidation | nterm | Other |  |  | + |  | DP |
| P06702 | Protein S100-A9 | S100A9 |  | DLQNFLK | Deamidation | N | Other |  | + | + | + | DP |
| P06702 | Protein S100-A9 | S100A9 |  | DLQNFLK | Comp.:13C-12C | Q | Other |  |  | + |  | DP |
| P06702 | Protein S100-A9 | S100A9 |  | DLQNFLK | Peptide:DLQNFLKK.E | cterm;K | Other |  | + |  | + | DP |
| P06702 | Protein S100-A9 | S100A9 |  | DLQNFLK | Peptide:R.KDLQNFLK | nterm | Other |  |  | + |  | DP |
| P06702 | Protein S100-A9 | S100A9 |  | KDLQNFLK | ID | D;L | Other |  | + |  | + | DP |
| P06702 | Protein S100-A9 | S100A9 |  | KDLQNFLK | Peptide:K.DLQNFLK | nterm | Other | + |  | + | + | DP |
| P06702 | Protein S100-A9 | S100A9 |  | KDLQNFLK | Peptide:KDLQNFL.K | L | Other |  |  | + |  | DP |
| P06702 | Protein S100-A9 | S100A9 |  | KDLQNFLK | Deamidation | Q | Other | + |  |  | + | DP |
| P06702 | Protein S100-A9 | S100A9 |  | KDLQNFLK | Acetaldehyde | nterm | Other |  |  |  | + | DP |
| P06702 | Protein S100-A9 | S100A9 |  | LGHPDTLNQGEFK | Peptide:LGHPDTLNQGEF.K | cterm;K | Other |  | + | + | + | DP |
| P06702 | Protein S100-A9 | S100A9 |  | LGHPDTLNQGEFK | Peptide:L.GHPDTLNQGEFK | nterm | Other |  |  |  | + | DP |
| P06702 | Protein S100-A9 | S100A9 |  | LGHPDTLNQGEFK | Comp.:12C-13C | N;Q | Other | + |  |  |  | DP |
| P06702 | Protein S100-A9 | S100A9 |  | LGHPDTLNQGEFK | Deamidation | N | Other |  |  |  | + | DP |
| P06702 | Protein S100-A9 | S100A9 |  | LGHPDTLNQGEFK | Acetaldehyde | G | Other |  | + | + | + | DP |
| P06702 | Protein S100-A9 | S100A9 |  | LGHPDTLNQGEFK | tri-Methylation | cterm;E;F;G;K | Other |  |  |  | + | DP |
| P06702 | Protein S100-A9 | S100A9 |  | LGHPDTLNQGEFK | K | cterm;K | Other |  |  |  | + | DP |
| P06702 | Protein S100-A9 | S100A9 |  | LGHPDTLNQGEFK | Peptide:V.KLGHPDTLNQGEFK | nterm | Other |  | + | + | + | DP |
| P06702 | Protein S100-A9 | S100A9 |  | LGHPDTLNQGEFK | R | cterm;F;K | Other |  |  |  | + | DP |
| P06702 | Protein S100-A9 | S100A9 |  | LGHPDTLNQGEFKELVR | Diffuse:3 | N;Q | Other |  | + | + |  | DP |
| P06702 | Protein S100-A9 | S100A9 |  | LGHPDTLNQGEFKELVRK | Peptide:LGHPDTLNQGEFKELVR.K | cterm;K;R;V | Other |  | + |  | + | DP |
| P06702 | Protein S100-A9 | S100A9 |  | LGHPDTLNQGEFKELVRK | Comp.:13C-12C | L;N;Q | Other |  |  | + |  | DP |
| P06702 | Protein S100-A9 | S100A9 |  | LTWASHEK | Peptide:L.TWASHEK | nterm | Other |  | + | + | + | DP |
| P06702 | Protein S100-A9 | S100A9 |  | LTWASHEK | Trp->Kynurenin | W | Other |  | + |  | + | DP |
| P06702 | Protein S100-A9 | S100A9 |  | LTWASHEK | Comp.:C | T | Other |  |  |  | + | DP |
| P06702 | Protein S100-A9 | S100A9 |  | LTWASHEK | Oxidation | W | Other |  | + | + | + | DP |
| P06702 | Protein S100-A9 | S100A9 |  | LTWASHEK | tri-Methylation | cterm;E;H;K | Other |  |  |  | + | DP |
| P06702 | Protein S100-A9 | S100A9 |  | MHEGDEGPGHHHK | Peptide:M.HEGDEGPGHHHK | nterm | Other | + | + | + | + | DP |
| P06702 | Protein S100-A9 | S100A9 |  | MHEGDEGPGHHHK | Reduction | nterm | Other |  | + | + |  | DP |
| P06702 | Protein S100-A9 | S100A9 |  | MHEGDEGPGHHHK | Diffuse:2 | E | Other |  |  | + |  | DP |
| P06702 | Protein S100-A9 | S100A9 |  | MHEGDEGPGHHHKPGLGEGTP | Peptide:E.GPGHHHKPGLGEGTP | nterm | Other |  |  |  | + | DP |
| P06702 | Protein S100-A9 | S100A9 |  | MHEGDEGPGHHHKPGLGEGTP | Peptide:G.DEGPGHHHKPGLGEGTP | nterm | Other |  |  |  | + | DP |
| P06702 | Protein S100-A9 | S100A9 |  | MHEGDEGPGHHHKPGLGEGTP | Peptide:MHEGDEGPGHHHKPGL.G | cterm;P | Other |  |  |  | + | DP |
| P06702 | Protein S100-A9 | S100A9 |  | MHEGDEGPGHHHKPGLGEGTP | Peptide:E.GDEGPGHHHKPGLGEGTP | nterm | Other |  |  |  | + | DP |
| P06702 | Protein S100-A9 | S100A9 |  | MHEGDEGPGHHHKPGLGEGTP | Peptide:MHEGDEGPGHHHKPGLG.E | cterm;P | Other |  |  |  | + | DP |
| P06702 | Protein S100-A9 | S100A9 |  | MHEGDEGPGHHHKPGLGEGTP | Peptide:M.HEGDEGPGHHHKPGLGEGTP | nterm | Other |  | + | + | + | DP |
| P06702 | Protein S100-A9 | S100A9 |  | MHEGDEGPGHHHKPGLGEGTP | Comp.:O-C4 | nterm | Other |  | + | + | + | DP |
| P06702 | Protein S100-A9 | S100A9 |  | MHEGDEGPGHHHKPGLGEGTP | Loss of water | D;E;G | Other |  | + | + | + | DP |
| P06702 | Protein S100-A9 | S100A9 |  | MHEGDEGPGHHHKPGLGEGTP | Loss of ammonia | D;E;G;H;P | Other |  |  | + | + | DP |
| P06702 | Protein S100-A9 | S100A9 |  | MHEGDEGPGHHHKPGLGEGTP | Reduction | nterm | Other |  | + | + | + | DP |
| P06702 | Protein S100-A9 | S100A9 |  | MHEGDEGPGHHHKPGLGEGTP | Comp.:13C-12C | P | Other | + | + | + | + | DP |
| P06702 | Protein S100-A9 | S100A9 |  | MHEGDEGPGHHHKPGLGEGTP | Diffuse:2 | cterm;P;T | Other |  | + |  | + | DP |
| P06702 | Protein S100-A9 | S100A9 |  | MHEGDEGPGHHHKPGLGEGTP | Diffuse:2 | E;G;H;K;L;P;T | Other |  |  |  | + | DP |
| P06702 | Protein S100-A9 | S100A9 |  | MHEGDEGPGHHHKPGLGEGTP | Oxidation | nterm | Other |  |  | + | + | DP |
| P06702 | Protein S100-A9 | S100A9 |  | MHEGDEGPGHHHKPGLGEGTP | Comp.:HCN | nterm | Other |  |  |  | + | DP |
| P06702 | Protein S100-A9 | S100A9 |  | MHEGDEGPGHHHKPGLGEGTP | Formylation | H | Other |  | + |  | + | DP |
| P06702 | Protein S100-A9 | S100A9 |  | MHEGDEGPGHHHKPGLGEGTP | di-Oxidation | E;G;H;K;L;P | Other | + | + | + | + | DP |
| P06702 | Protein S100-A9 | S100A9 |  | MHEGDEGPGHHHKPGLGEGTP | tri-Methylation | E;G;H;K;L;P;T | Other |  |  |  | + | DP |
| P06702 | Protein S100-A9 | S100A9 |  | MHEGDEGPGHHHKPGLGEGTP | Phosphorylation | T | Other | + | + | + | + | DP |
| P06702 | Protein S100-A9 | S100A9 |  | MHEGDEGPGHHHKPGLGEGTP | GlyGly | H | Other | + | + | + | + | DP |
| P06702 | Protein S100-A9 | S100A9 |  | MHEGDEGPGHHHKPGLGEGTP | D | G;H;P | Other |  | + | + | + | DP |
| P06702 | Protein S100-A9 | S100A9 |  | NIETIINTFHQYSVK | Peptide:I.INTFHQYSVK | I | Other |  | + |  | + | DP |
| P06702 | Protein S100-A9 | S100A9 |  | NIETIINTFHQYSVK | Peptide:NIETIINTFHQY.S | cterm;K | Other | + | + | + | + | DP |
| P06702 | Protein S100-A9 | S100A9 |  | NIETIINTFHQYSVK | Peptide:NIETIINTFHQYSV.K | cterm;H;K;Q;S;V;Y | Other |  | + |  | + | DP |
| P06702 | Protein S100-A9 | S100A9 |  | NIETIINTFHQYSVK | Comp.:12C-13C | N | Other |  |  |  | + | DP |
| P06702 | Protein S100-A9 | S100A9 |  | NIETIINTFHQYSVK | Diffuse:2 | E | Other |  |  | + |  | DP |
| P06702 | Protein S100-A9 | S100A9 |  | NIETIINTFHQYSVK | Deamidation | N | Other |  |  |  | + | DP |
| P06702 | Protein S100-A9 | S100A9 |  | NIETIINTFHQYSVK | Comp.:13C-12C | I;T | Other |  |  |  | + | DP |
| P06702 | Protein S100-A9 | S100A9 |  | NIETIINTFHQYSVK | Diffuse:2 | E;I;T | Other |  |  | + |  | DP |
| P06702 | Protein S100-A9 | S100A9 |  | NIETIINTFHQYSVK | Diffuse:4 | I;N;T | Other |  | + |  |  | DP |
| P06702 | Protein S100-A9 | S100A9 |  | NIETIINTFHQYSVK | Formylation | nterm | Other |  |  |  | + | DP |
| P06702 | Protein S100-A9 | S100A9 |  | VIEHIMEDLDTNADK | Peptide:M.EDLDTNADK | nterm | Other | + | + |  |  | DP |
| P06702 | Protein S100-A9 | S100A9 |  | VIEHIMEDLDTNADK | Peptide:M.EDLDTNADK | nterm | Other |  |  | + | + | DP |
| P06702 | Protein S100-A9 | S100A9 |  | VIEHIMEDLDTNADK | Peptide:VIEHIMEDL.D | cterm;K | Other |  | + |  |  | DP |
| P06702 | Protein S100-A9 | S100A9 |  | VIEHIMEDLDTNADK | Peptide:H.IMEDLDTNADK | nterm | Other |  | + | + | + | DP |
| P06702 | Protein S100-A9 | S100A9 |  | VIEHIMEDLDTNADK | Peptide:VIEHIMEDLDTN.A | cterm;K | Other | + | + | + | + | DP |
| P06702 | Protein S100-A9 | S100A9 |  | VIEHIMEDLDTNADK | Peptide:I.EHIMEDLDTNADK | nterm | Other | + | + | + | + | DP |
| P06702 | Protein S100-A9 | S100A9 |  | VIEHIMEDLDTNADK | Peptide:V.IEHIMEDLDTNADK | nterm | Other | + | + | + | + | DP |
| P06702 | Protein S100-A9 | S100A9 |  | VIEHIMEDLDTNADK | Reduction | M | Other | + | + | + | + | DP |
| P06702 | Protein S100-A9 | S100A9 |  | VIEHIMEDLDTNADK | Comp.:12C-13C | A;N | Other | + | + | + |  | DP |
| P06702 | Protein S100-A9 | S100A9 |  | VIEHIMEDLDTNADK | Deamidation | N | Other | + | + | + | + | DP |
| P06702 | Protein S100-A9 | S100A9 |  | VIEHIMEDLDTNADK | Comp.:13C-12C | nterm | Other |  |  |  | + | DP |
| P06702 | Protein S100-A9 | S100A9 |  | VIEHIMEDLDTNADK | Diffuse:2 | E;L | Other |  |  |  | + | DP |
| P06702 | Protein S100-A9 | S100A9 |  | VIEHIMEDLDTNADK | Diffuse:3 | I | Other |  |  | + |  | DP |
| P06702 | Protein S100-A9 | S100A9 |  | VIEHIMEDLDTNADK | Oxidation | M | Other | + | + | + | + | DP |
| P06702 | Protein S100-A9 | S100A9 |  | VIEHIMEDLDTNADK | Comp.:H3C2N | E;H;I;M | Other |  | + | + | + | DP |
| P06702 | Protein S100-A9 | S100A9 |  | VIEHIMEDLDTNADK | R | cterm;K | Other |  |  |  | + | DP |
| P06733 | Alpha-enolase | ENO1 |  | YISPDQLADLYK | Methylation | S | Other | + |  |  |  | DP |
| P08311 | Cathepsin G | CTSG |  | IFGSYDPR | Acetaldehyde | nterm | Other |  | + |  | + | DP |
| P08311 | Cathepsin G | CTSG |  | TIQNDIMLLQLSR | Reduction | M | Other |  |  |  | + | DP |
| P08311 | Cathepsin G | CTSG |  | TIQNDIMLLQLSR | Diffuse:3 | D;I;N;Q | Other |  | + |  |  | DP |
| P08311 | Cathepsin G | CTSG |  | TIQNDIMLLQLSR | Diffuse:3 | D;N | Other |  | + |  |  | DP |
| P08311 | Cathepsin G | CTSG |  | TIQNDIMLLQLSR | Oxidation | L;M | Other |  | + |  |  | DP |
| P08311 | Cathepsin G | CTSG |  | VSSFLPWIR | Peptide:V.SSFLPWIR | nterm | Other |  | + |  |  | DP |
| P08311 | Cathepsin G | CTSG |  | VSSFLPWIR | di-Oxidation | cterm;I;P;R;W | Other |  | + |  |  | DP |
| P10153 | Non-secretory ribonuclease | RNASE2 |  | RDPPQYPVVPVHLDR | Peptide:D.PPQYPVVPVHLDR | nterm | Other |  |  |  | + | DP |
| P11021 | 78 kDa glucose-regulated protein | HSPA5 |  | IEWLESHQDADIEDFK | Comp.:13C-12C | A;D;E;H;L;Q;S;W | Other |  | + |  |  | DP |
| P11021 | 78 kDa glucose-regulated protein | HSPA5 |  | IEWLESHQDADIEDFK | Diffuse:4 | E;H;L;S | Other |  |  | + |  | DP |
| P11215 | Integrin alpha-M | ITGAM |  | LPSHSDFLAELR | Diffuse:3 | nterm | Other |  |  |  | + | DP |
| P13639 | Elongation factor 2 | EEF2 |  | TFCQLILDPIFK | Comp.:C | nterm | Other | + | + |  |  | DP |
| P17600 | Synapsin-1 | SYN1 |  | VLLVIDEPHTDWAK | Comp.:-H4O2 | I | Other | + |  |  |  | DP |
| P20160 | Azurocidin | AZU1 |  | GPDFFTR | Comp.:13C-12C | D | Other |  | + |  | + | DP |
| P20160 | Azurocidin | AZU1 |  | GPDFFTR | Diffuse:2 | D;F | Other |  |  | + |  | DP |
| P20160 | Azurocidin | AZU1 |  | GPDFFTR | tri-Methylation | F;T | Other |  | + |  |  | DP |
| P20160 | Azurocidin | AZU1 |  | HFCGGALIHAR | Comp.:-H5C2NOS | C | Other |  | + |  |  | DP |
| P20160 | Azurocidin | AZU1 |  | HFCGGALIHAR | Comp.:-CH4O | C | Other |  | + |  |  | DP |
| P20160 | Azurocidin | AZU1 |  | HFCGGALIHAR | Comp.:13C-12C | A;C;I;L | Other |  | + |  |  | DP |
| P20160 | Azurocidin | AZU1 |  | HFCGGALIHAR | Oxidation | A;C;G | Other |  | + |  |  | DP |
| P20160 | Azurocidin | AZU1 |  | HFCGGALIHAR | Sulfide | C | Other |  | + |  | + | DP |
| P20160 | Azurocidin | AZU1 |  | QFPFLASIQNQGR | Loss of ammonia | nterm | Other |  | + |  |  | DP |
| P20160 | Azurocidin | AZU1 |  | QFPFLASIQNQGR | Deamidation | N | Other |  | + |  |  | DP |
| P20160 | Azurocidin | AZU1 |  | QFPFLASIQNQGR | Diffuse:2 | nterm | Other |  | + |  |  | DP |
| P20160 | Azurocidin | AZU1 |  | QFPFLASIQNQGR | Acetaldehyde | nterm | Other |  | + |  |  | DP |
| P21333 | Filamin-A | FLNA |  | FADQHVPGSPFSVK | Diffuse:2 | D;H;P;Q;V | Other |  |  |  | + | DP |
| P24158 | Myeloblastin | PRTN3 |  | PYMASLQMR | Reduction | M | Other |  | + |  | + | DP |
| P24158 | Myeloblastin | PRTN3 |  | LVNVVLGAHNVR | Peptide:LVNVVLGAH.N | cterm;R | Other |  | + |  |  | DP |
| P30044 | Peroxiredoxin-5, mitochondrial | PRDX5 |  | ETDLLLDDSLVSIFGNR | Comp.:-C2H2O2 | nterm | Other | + |  |  |  | DP |
| P30740 | Leukocyte elastase inhibitor | SERPINB1 |  | TFHFNTVEEVHSR | Comp.:12C-13C | F;N;T | Other |  |  |  | + | DP |
| P30740 | Leukocyte elastase inhibitor | SERPINB1 |  | TFHFNTVEEVHSR | Comp.:13C-12C | V | Other |  |  | + |  | DP |
| P30740 | Leukocyte elastase inhibitor | SERPINB1 |  | TYGADLASVDFQHASEDAR | Diffuse:2 | A;D;G;L;S;V | Other |  |  | + |  | DP |
| P30740 | Leukocyte elastase inhibitor | SERPINB1 |  | LEESYTLNSDLAR | Diffuse:2 | E | Other |  |  |  | + | DP |
| P30740 | Leukocyte elastase inhibitor | SERPINB1 |  | LGVQDLFNSSK | Deamidation | F;N | Other |  |  |  | + | DP |
| P31949 | Protein S100-A11 | S100A11 |  | DPGVLDR | Comp.:13C-12C | nterm | Other |  |  | + |  | DP |
| P31949 | Protein S100-A11 | S100A11 |  | TEFLSFMNTELAAFTK | Comp.:C | nterm | Other | + |  |  |  | DP |
| P31997 | Carcinoembryonic antigen-related cell adhesion molecule 8 | CEACAM8 |  | EVLLLVHNLPQDPR | Peptide:EVLLLVHNLPQD.P | D;P;Q | Other |  | + | + | + | DP |
| P31997 | Carcinoembryonic antigen-related cell adhesion molecule 8 | CEACAM8 |  | EVLLLVHNLPQDPR | Loss of water | nterm | Other | + | + | + | + | DP |
| P31997 | Carcinoembryonic antigen-related cell adhesion molecule 8 | CEACAM8 |  | EVLLLVHNLPQDPR | Peptide:EVLLLVHNLPQDPRG.Y | H;L;N | Other |  | + | + |  | DP |
| P35579 | Myosin-9 | MYH9 |  | HEAMITDLEER | Oxidation | A;M | Other |  | + |  |  | DP |
| P35579 | Myosin-9 | MYH9 |  | HSQAVEELAEQLEQTK | Diffuse:2 | L | Other |  | + |  |  | DP |
| P35579 | Myosin-9 | MYH9 |  | HSQAVEELAEQLEQTK | Diffuse:3 | L | Other |  |  | + |  | DP |
| P35579 | Myosin-9 | MYH9 |  | DLEAHIDSANK | Comp.:-H4O2 | I | Other |  | + |  | + | DP |
| P35579 | Myosin-9 | MYH9 |  | EMEAELEDER | Reduction | nterm | Other |  |  |  | + | DP |
| P35579 | Myosin-9 | MYH9 |  | EMEAELEDERK | Peptide:V.REMEAELEDERK | nterm | Other |  |  |  | + | DP |
| P35579 | Myosin-9 | MYH9 |  | IAQLEEQLDNETK | Peptide:I.AQLEEQLDNETK | nterm | Other |  |  |  | + | DP |
| P35579 | Myosin-9 | MYH9 |  | RQLEEAEEEAQR | Comp.:12C-13C | A;E | Other |  |  |  | + | DP |
| P35579 | Myosin-9 | MYH9 |  | RQLEEAEEEAQR | Peptide:RQLEEAEEEAQRA.N | cterm;R | Other |  |  |  | + | DP |
| P42677 | 40S ribosomal protein S27 | RPS27 |  | DLLHPSPEEEK | Diffuse:2 | E;P;S | Other | + |  |  |  | DP |
| P60709 | Actin, cytoplasmic 1 | ACTB |  | DLYANTVLSGGTTMYPGIADR | Peptide:T.VLSGGTTMYPGIADR | nterm | Other |  | + |  |  | DP |
| P62805 | Histone H4 | HIST1H4A |  | DAVTYTEHAK | IN | nterm | Other |  |  | + |  | DP |
| P62805 | Histone H4 | HIST1H4A |  | DNIQGITKPAIR | Peptide:DNIQGITKPA.I | A;cterm;I;P;R | Other |  |  |  | + | DP |
| P62805 | Histone H4 | HIST1H4A |  | ISGLIYEETR | Peptide:L.IYEETR | S | Other |  |  | + | + | DP |
| P62805 | Histone H4 | HIST1H4A |  | ISGLIYEETR | Peptide:I.SGLIYEETR | I | Other | + | + | + | + | DP |
| P62805 | Histone H4 | HIST1H4A |  | ISGLIYEETR | Comp.:-H4O2 | nterm | Other | + | + | + |  | DP |
| P62805 | Histone H4 | HIST1H4A |  | ISGLIYEETR | Acetaldehyde | nterm | Other | + |  |  |  | DP |
| P62805 | Histone H4 | HIST1H4A |  | VFLENVIR | Peptide:V.FLENVIR | nterm | Other | + | + | + | + | DP |
| P62805 | Histone H4 | HIST1H4A |  | VFLENVIR | Acetaldehyde | nterm | Other | + |  |  |  | DP |
| P68363 | Tubulin alpha-1B chain | TUBA1B |  | SIQFVDWCPTGFK | Methylation | nterm | Other |  | + |  |  | DP |
| P68371 | Tubulin beta-2A chain | TUBB2A |  | MSATFIGNSTAIQELFK | Reduction | nterm | Other |  | + |  |  | DP |
| P69905 | Hemoglobin subunit alpha | HBA1 |  | VGAHAGEYGAEALER | Peptide:V.GAHAGEYGAEALER | A;H | Other |  | + |  |  | DP |
| P69905 | Hemoglobin subunit alpha | HBA1 |  | VGAHAGEYGAEALER | Acetaldehyde | nterm | Other |  | + |  |  | DP |
| P80511 | Calcitermin | S100A12 |  | AAHYHTHK | Peptide:AAHYHTHKE. | cterm;K | Other |  |  | + |  | DP |
| P80511 | Calcitermin | S100A12 |  | GHFDTLSK | Peptide:G.HFDTLSK | nterm | Other | + | + |  | + | DP |
| P80511 | Calcitermin | S100A12 |  | GHFDTLSK | Peptide:R.KGHFDTLSK | nterm | Other |  |  |  | + | DP |
| P80511 | Calcitermin | S100A12 |  | GHFDTLSKGELK | Comp.:13C-12C | L;T | Other |  | + |  |  | DP |
| Q05996 | Processed zona pellucida sperm-binding protein 2 | ZP2 |  | ATYDNCTRR | Comp.:-H5C2NOS | C;D;N;R;T;Y | Other |  | + |  | + | DP |
| Q05996 | Processed zona pellucida sperm-binding protein 2 | ZP2 |  | ATYDNCTRR | Comp.:-CH4O | C;D;N;R;T;Y | Other |  |  | + |  | DP |
| Q05996 | Processed zona pellucida sperm-binding protein 2 | ZP2 |  | ATYDNCTRR | Sulfide | C;D;N;R;T;Y | Other | + | + |  |  | DP |
| Q09666 | Neuroblast differentiation-associated protein AHNAK | AHNAK |  | ISMPDVNLNLK | Peptide:I.SMPDVNLNLK | L | Other | + | + |  |  | DP |
| Q09666 | Neuroblast differentiation-associated protein AHNAK | AHNAK |  | ISMPDVNLNLK | Loss of ammonia | nterm | Other | + |  |  |  | DP |
| Q09666 | Neuroblast differentiation-associated protein AHNAK | AHNAK |  | ISMPDVNLNLK | Comp.:-H4O2 | D;M;N;P;S;V | Other | + |  |  |  | DP |
| Q09666 | Neuroblast differentiation-associated protein AHNAK | AHNAK |  | ISMPDVNLNLK | Diffuse:2 | nterm | Other | + |  |  |  | DP |
| Q09666 | Neuroblast differentiation-associated protein AHNAK | AHNAK |  | ISMPDVNLNLK | Deamidation | N | Other | + | + |  |  | DP |
| Q09666 | Neuroblast differentiation-associated protein AHNAK | AHNAK |  | ISMPDVNLNLK | Comp.:C | D;M;N;P;S;V | Other | + |  |  |  | DP |
| Q09666 | Neuroblast differentiation-associated protein AHNAK | AHNAK |  | ISMPDVNLNLK | Acetaldehyde | D;M;N;P;S;V | Other | + |  |  |  | DP |
| Q09666 | Neuroblast differentiation-associated protein AHNAK | AHNAK |  | ISMPDVNLNLK | Formylation | cterm;K;L | Other | + | + |  |  | DP |
| Q09666 | Neuroblast differentiation-associated protein AHNAK | AHNAK |  | ISMPDVNLNLK | tri-Oxidation | D;M;N;P;S;V | Other | + |  |  |  | DP |
| Q09666 | Neuroblast differentiation-associated protein AHNAK | AHNAK |  | ISMPDVNLNLK | Peptide:ISMPDVNLNLKG.P | cterm;K;L | Other | + | + |  |  | DP |
| Q09666 | Neuroblast differentiation-associated protein AHNAK | AHNAK |  | ISMPDVNLNLK | Comp.:C3H4O2 | D;M;N;P;S;V | Other | + |  |  |  | DP |
| Q09666 | Neuroblast differentiation-associated protein AHNAK | AHNAK |  | VSMPDVELNLK | Comp.:-HN | nterm | Other |  | + | + |  | DP |
| Q09666 | Neuroblast differentiation-associated protein AHNAK | AHNAK |  | VSMPDVELNLK | Comp.:ON2-C2H3S | nterm | Other |  |  |  | + | DP |
| Q09666 | Neuroblast differentiation-associated protein AHNAK | AHNAK |  | VSMPDVELNLK | Comp.:-H4O2 | nterm | Other |  |  | + | + | DP |
| Q09666 | Neuroblast differentiation-associated protein AHNAK | AHNAK |  | VSMPDVELNLK | Comp.:12C-13C | nterm | Other |  | + | + | + | DP |
| Q09666 | Neuroblast differentiation-associated protein AHNAK | AHNAK |  | VSMPDVELNLK | Deamidation | L;N | Other |  | + | + |  | DP |
| Q09666 | Neuroblast differentiation-associated protein AHNAK | AHNAK |  | VSMPDVELNLK | Comp.:13C-12C | E;L;N | Other |  |  | + |  | DP |
| Q09666 | Neuroblast differentiation-associated protein AHNAK | AHNAK |  | VSMPDVELNLK | Formylation | cterm;K;L | Other |  |  |  | + | DP |
| Q09666 | Neuroblast differentiation-associated protein AHNAK | AHNAK |  | VSMPDVELNLK | Propionyl | nterm | Other |  | + | + | + | DP |
| Q562R1 | Beta-actin-like protein 2 | ACTBL2 |  | AGFGGDDAPR | N | nterm | Other |  | + |  |  | DP |
| Q5SZK5 | Wiskott-Aldrich syndrome protein family member 1 | WASF1 |  | FYTNPSYFFDLWK | Deamidation | N;T | Other | + |  |  |  | DP |
| Q6PP77 | XK-related protein 2 | XKRX |  | SGAQMPNNIEK | Loss of water | nterm | Other | + |  |  |  | DP |
| Q8IUE6 | Histone H2A type 2-B | HIST2H2AB |  | HLQLAVR | Methylation | V | Other |  | + |  |  | DP |
| Q8IZU3 | Synaptonemal complex protein 3 | SYCP3 |  | ILNMFRQQQK | Comp.:12C-13C | cterm;F;K;M;Q;R | Other |  | + |  | + | DP |
| Q8TAT5 | Endonuclease 8-like 3 | NEIL3 |  | ITVCRFGDNNR | A | cterm;N;R | Other |  |  |  | + | DP |
| Q92930 | Ras-related protein Rab-8B | RAB8B |  | KTSFFRCSLL | Peptide:KTSFFRCSL.L | nterm | Other | + |  | + |  | DP |
| Q92930 | Ras-related protein Rab-8B | RAB8B |  | KTSFFRCSLL | K | cterm;L | Other |  |  | + |  | DP |
| Q9H156 | SLIT and NTRK-like protein 2 | SLITRK2 |  | QNQDRINK | Peptide:QNQDRIN.K | N | Other | + | + | + | + | DP |
| Q9H156 | SLIT and NTRK-like protein 2 | SLITRK2 |  | QNQDRINK | N | N | Other |  |  | + |  | DP |
| Q9H156 | SLIT and NTRK-like protein 2 | SLITRK2 |  | QNQDRINK | Loss of water | N | Other | + | + | + | + | DP |
| Q9H156 | SLIT and NTRK-like protein 2 | SLITRK2 |  | QNQDRINK | Loss of ammonia | N | Other | + | + | + | + | DP |
| Q9H156 | SLIT and NTRK-like protein 2 | SLITRK2 |  | QNQDRINK | Reduction | N | Other | + |  |  |  | DP |
| Q9H156 | SLIT and NTRK-like protein 2 | SLITRK2 |  | QNQDRINK | Comp.:O-NH3 | nterm | Other |  |  | + |  | DP |
| Q9H156 | SLIT and NTRK-like protein 2 | SLITRK2 |  | QNQDRINK | Comp.:12C-13C | Q | Other | + |  |  | + | DP |
| Q9H156 | SLIT and NTRK-like protein 2 | SLITRK2 |  | QNQDRINK | Deamidation | N | Other | + | + | + | + | DP |
| Q9H156 | SLIT and NTRK-like protein 2 | SLITRK2 |  | QNQDRINK | Comp.:C | nterm | Other | + | + | + | + | DP |
| Q9H156 | SLIT and NTRK-like protein 2 | SLITRK2 |  | QNQDRINK | Acetaldehyde | D;Q;R | Other | + |  | + |  | DP |
| Q9H156 | SLIT and NTRK-like protein 2 | SLITRK2 |  | QNQDRINK | Formylation | cterm;K | Other |  |  | + |  | DP |
| Q9H156 | SLIT and NTRK-like protein 2 | SLITRK2 |  | QNQDRINK | Comp.:N2 | cterm;K | Other | + | + | + | + | DP |
| Q9H156 | SLIT and NTRK-like protein 2 | SLITRK2 |  | QNQDRINK | tri-Methylation | nterm | Other | + | + | + | + | DP |
| Q9H156 | SLIT and NTRK-like protein 2 | SLITRK2 |  | QNQDRINK | GlyGly | nterm | Other | + | + | + | + | DP |
| Q9H156 | SLIT and NTRK-like protein 2 | SLITRK2 |  | QNQDRINK | EI | nterm | Other | + |  |  | + | DP |
| Q9H156 | SLIT and NTRK-like protein 2 | SLITRK2 |  | QNQDRINK | Peptide:QNQDRINKTVL.Y | cterm;K;N | Other |  |  |  | + | DP |
| Q9Y3C8 | Ubiquitin-fold modifier-conjugating enzyme 1 | UFC1 |  | TNAGPRDR | Comp.:12C-13C | N | Other | + |  |  |  | DP |
| Q9Y3C8 | Ubiquitin-fold modifier-conjugating enzyme 1 | UFC1 |  | TNAGPRDR | Comp.:C | N | Other |  | + | + |  | DP |
| Q9Y3C8 | Ubiquitin-fold modifier-conjugating enzyme 1 | UFC1 |  | TNAGPRDR | Acetaldehyde | nterm | Other |  | + | + |  | DP |
| Q9Y3C8 | Ubiquitin-fold modifier-conjugating enzyme 1 | UFC1 |  | TNAGPRDR | Carboxymethyl | nterm | Other | + | + |  | + | DP |
| H3BT58 | Coactosin-like protein | COTL1 | FALITWIGENVSGLQRAKTGTDKTLVKEVVQ |  | Citrullination | R | D |  | + |  |  | VM |
| C9JYJ6 | Filamin A-interacting protein 1-like | FILIP1L | RSPEPTEISAKHAIFRVSPDRQSSWQFQRSN |  | Citrullination | R | D |  |  |  | + | VM |
| C9JYJ6 | Filamin A-interacting protein 1-like | FILIP1L | TEISAKHAIFRVSPDRQSSWQFQRSNSNSSS |  | Citrullination | R | D |  |  |  | + | VM |
| P30793-3 | GTP cyclohydrolase 1 | GCH1 | _________MEKGPVRAPAEKPRGARCSNGF |  | Citrullination | R | D |  | + |  |  | VM |
| B9ZVN9 | DNA-directed RNA polymerase | POLR1A | QRIIEESTHCGPQAVRAALNLPEAASYDEVR |  | Citrullination | R | D |  | + |  |  | VM |
| A1A4F0 | Putative uncharacterized protein PQLC2L | PQLC2L | KKMIFQPQLFKDSITREKVRLSLWGVLCPVY |  | Citrullination | R | D |  |  | + |  | VM |
| B8ZZQ6 | Prothymosin alpha | PTMA | EDGDEDEEAESATGKRAAEDDEDDDVDTKKQ |  | Citrullination | R | D | + | + |  |  | VM |
| C9IYJ2 | PX domain-containing protein kinase-like protein | PXK | WGLQPRFQEYIIRVQRGISVENSWQIVRRYS |  | Citrullination | R | D |  | + |  |  | VM |
| C9IYJ2 | PX domain-containing protein kinase-like protein | PXK | RVQRGISVENSWQIVRRYSDFDLLNNSLQIA |  | Citrullination | R | D |  | + |  |  | VM |
| Q9H156 | SLIT and NTRK-like protein 2 | SLITRK2 | RQFAPSYESRRQNQDRINKTVLYGTPRKCFV |  | Citrullination | R | D | + | + | + | + | VM |
| C9JWN9 | Tensin-3 | TNS3 | GCSEESYLHNLQEVTRMLKSKHGDNYLVLNL |  | Citrullination | R | D | + |  | + |  | VM |
| F8W8C1 | E3 ubiquitin-protein ligase TRIM9 | TRIM67 | LGKDDKAWAMYVDNNRSWFMHCNSHTNRTEG |  | Citrullination | R | D | + | + | + | + | VM |
| Q9Y3C8 | Ubiquitin-fold modifier-conjugating enzyme 1 | UFC1 | RVVSEIPVLKTNAGPRDRELWVQRLKEEYQS |  | Citrullination | R | D | + | + | + | + | VM |
| Q9Y3C8 | Ubiquitin-fold modifier-conjugating enzyme 1 | UFC1 | VSEIPVLKTNAGPRDRELWVQRLKEEYQSLI |  | Citrullination | R | D | + | + | + | + | VM |
| P63261 | Actin, cytoplasmic 1 | ACTB | TTAEREIVRDIKEKLCYVALDFEQEMATAAS |  | Alkylation | C | IAA | + |  |  |  | VM |
| O43823 | A-kinase anchor protein 8 | AKAP8 | RDRTRDRAADRIQFACSVCKFRSFDDEEIQK |  | Alkylation | C | IAA |  |  |  | + | VM |
| O43823 | A-kinase anchor protein 8 | AKAP8 | TRDRAADRIQFACSVCKFRSFDDEEIQKHLQ |  | Alkylation | C | IAA |  |  |  | + | VM |
| M0R116 | Potassium-transporting ATPase alpha chain 2 | ATP12A | FARTSPQQKLIIVEGCQRQGAIVAVTGDGVN |  | Alkylation | C | IAA | + | + |  |  | VM |
| M0R116 | Sodium/potassium-transporting ATPase subunit alpha-1 | ATP1A1 | KNLEAVETLGSTSTICSDKTGTLTQNRMTVA |  | Alkylation | C | IAA | + |  |  |  | VM |
| P20160 | Azurocidin | AZU1 | QFPFLASIQNQGRHFCGGALIHARFVMTAAS |  | Alkylation | C | IAA |  | + |  | + | VM |
| Q9UQM7 | Calcium/calmodulin-dependent protein kinase type II subunit alpha | CAMK2A | IEAISNGDFESYTKMCDPGMTAFEPEALGNL |  | Alkylation | C | IAA | + |  |  |  | VM |
| Q6TFL3-3 | Coiled-coil domain-containing protein 171 | CCDC171 | DYQNKLEDASNEEKACNELDSTKQKIDSHTK |  | Alkylation | C | IAA | + |  |  |  | VM |
| P86791 | Vacuolar fusion protein CCZ1 homolog | CCZ1 | EEELLDKVYSSVLRQCYSMYKLFNGTFLKAM |  | Alkylation | C | IAA |  | + |  | + | VM |
| G3V1A4 | Cofilin-1 | CFL1 | STPEEVKKRKKAVLFCLSEDKKNIILEEGKE |  | Alkylation | C | IAA | + |  |  |  | VM |
| G3V1A4 | Cofilin-1 | CFL1 | DDPYATFVKMLPDKDCRYALYDATYETKESK |  | Alkylation | C | IAA |  | + | + | + | VM |
| P12277 | Creatine kinase B-type | CKB | SMQKGGNMKEVFTRFCTGLTQIETLFKSKDY |  | Alkylation | C | IAA | + | + |  |  | VM |
| I3L2V2 |  | DGKE | ______________HCAQIQVKLANPFRIGQ |  | Alkylation | C | IAA |  | + |  | + | VM |
| H7C4R7 | Deoxyribonuclease | DNASE1L3 | LIGDQEDTTVKKSTNCAYDRPWMSATTFQLN |  | Alkylation | C | IAA | + | + | + | + | VM |
| A0A1C7CYX9 | Dihydropyrimidinase-related protein 2 | DPYSL2 | AELRGVPRGLYDGPVCEVSVTPKTVTPASSA |  | Alkylation | C | IAA |  | + |  |  | VM |
| Q5VTE0 | Elongation factor 1-alpha | EEF1A1 | KDGNASGTTLLEALDCILPPTRPTDKPLRLP |  | Alkylation | C | IAA | + | + |  |  | VM |
| P13639 | Elongation factor 2 | EEF2 | KSATSPEGKKLPRTFCQLILDPIFKVFDAIM |  | Alkylation | C | IAA | + | + |  |  | VM |
| P06733 | Alpha-enolase | ENO1 | AKFGANAILGVSLAVCKAGAAERELPLYRHI;SKFGANAILGVSLAVCKAGAVEKGVPLYRHI |  | Alkylation | C | IAA | + |  |  |  | VM |
| P06733 | Alpha-enolase | ENO1 | GETEDTFIADLVVGLCTGQIKTGAPCRSERL |  | Alkylation | C | IAA | + |  |  |  | VM |
| M0QZ95 | F-box only protein 17 | FBXO17 | QIEICVADWWGARENCGCVYQLRVRLLDVYE |  | Alkylation | C | IAA | + |  |  | + | VM |
| M0QZ95 | F-box only protein 17 | FBXO17 | EICVADWWGARENCGCVYQLRVRLLDVYEKE |  | Alkylation | C | IAA | + |  |  | + | VM |
| J3QTJ6 | Fibrous sheath-interacting protein 2 | FSIP2 | RNKSFSMHRNNSVPLCNKINRQASPRDWQFS |  | Alkylation | C | IAA |  | + |  |  | VM |
| P04406-2 | Glyceraldehyde-3-phosphate dehydrogenase | GAPDH | AFRVPTANVSVVDLTCRLEKPAKYDDIKKVV |  | Alkylation | C | IAA | + |  |  |  | VM |
| Q92616 | Translational activator GCN1 | GCN1L1 | LYFSESLVPTARKALCDPLEEVREAAAKTFE |  | Alkylation | C | IAA |  | + |  |  | VM |
| P62873-2 | Guanine nucleotide-binding protein G(I)/G(S)/G(T) subunit beta-1 | GNB1 | SKSGRLLLAGYDDFNCNVWDALKADRAGVLA |  | Alkylation | C | IAA | + |  |  |  | VM |
| E7EP32 | Guanine nucleotide-binding protein G(I)/G(S)/G(T) subunit beta-2 | GNB2 | SRSGRLLLAGYDDFNCNIWDAMKGDRAGVLA |  | Alkylation | C | IAA | + |  |  |  | VM |
| A0A0C4DGJ9 | Granzyme H | GZMH | AAHCQGRGDSGGPLVCKDVAQGILSYGNKKG |  | Alkylation | C | IAA | + |  |  | + | VM |
| O14782 | Kinesin-like protein KIF3C | KIF3C | LERPSTSKVRKSRSWCQSPQRPPPSTTHASL |  | Alkylation | C | IAA | + | + | + | + | VM |
| P40926 | Malate dehydrogenase | MDH2 | IGGHAGKTIIPLISQCTPKVDFPQDQLTALT |  | Alkylation | C | IAA | + | + |  |  | VM |
| P05164-2 | 84 kDa myeloperoxidase | EPX | HDDPCLLTNRSARIPCFLAGDTRSSEMPELT;HDDPCLLTNRSARIPCFLAGDTRSTETPKLA |  | Alkylation | C | IAA |  | + |  | + | VM |
| H0Y6U5 | Condensin-2 complex subunit G2 | NCAPG2 | AFDTSSADVRCSVFKCLPMILDNKLSHPLLE |  | Alkylation | C | IAA |  |  |  | + | VM |
| Q8TAT5 | Endonuclease 8-like 3 | NEIL3 | YKRPNCGQCHCRITVCRFGDNNRMTYFCPHC |  | Alkylation | C | IAA |  |  |  | + | VM |
| I3L0N3 | Vesicle-fusing ATPase | NSF | NSGLHIIIFDEIDAICKQRGSMAGSTGVHDT |  | Alkylation | C | IAA |  | + |  |  | VM |
| P18669 | Phosphoglycerate mutase 1 | PGAM1 | RGGQALRDAGYEFDICFTSVQKRAIRTLWTV |  | Alkylation | C | IAA | + | + |  |  | VM |
| H3BTN5 | Pyruvate kinase | PKM | DIDSPPITARNTGIICTIGPASRSVETLKEM |  | Alkylation | C | IAA | + |  |  |  | VM |
| B9ZVN9 | DNA-directed RNA polymerase | POLR1A | KADVKRQRIIEESTHCGPQAVRAALNLPEAA |  | Alkylation | C | IAA |  | + |  |  | VM |
| P62937 | Peptidyl-prolyl cis-trans isomerase | PPIA | GYKGSCFHRIIPGFMCQGGDFTRHNGTGGKS |  | Alkylation | C | IAA | + | + |  |  | VM |
| Q96QC0 | Serine/threonine-protein phosphatase 1 regulatory subunit 10 | PPP1R10 | MSNRPVCRHFMMKGNCRYENNCAFYHPGVNG |  | Alkylation | C | IAA |  |  | + |  | VM |
| D6RD68 | Histone-lysine N-methyltransferase PRDM9 | PRDM7 | ALITIGLRATRPAFMCHRRQAIKLQVDDTED |  | Alkylation | C | IAA | + |  |  |  | VM |
| H0Y380 | Protein-tyrosine-phosphatase | PTPRD | SSKAHTSRFISANLPCNKFKNRLVNIMPYEL |  | Alkylation | C | IAA |  |  |  | + | VM |
| Q92930 | Ras-related protein Rab-8B | RAB8B | VKITENRSKKTSFFRCSLL____________ |  | Alkylation | C | IAA | + |  | + |  | VM |
| P05109 | Protein S100-A8 | S100A8 | HAVYRDDLKKLLETECPQYIRKKGADVWFKE |  | Alkylation | C | IAA | + | + | + | + | VM |
| P17600-2 | Synapsin-1 | SYN1 | EQIAMSDRYKLWVDTCSEIFGGLDICAVEAL |  | Alkylation | C | IAA |  | + |  |  | VM |
| P17600-2 | Synapsin-1 | SYN1 | LWVDTCSEIFGGLDICAVEALHGKDGRDHII |  | Alkylation | C | IAA |  | + |  |  | VM |
| A0A087X2E3 | Synapsin-2 | SYN2 | VHGKDGKDYIFEVMDCSMPLIGEHQVEDRQL |  | Alkylation | C | IAA | + | + |  |  | VM |
| C9JWN9 | Tensin-3 | TNS3 | TYITERIIAVSFPAGCSEESYLHNLQEVTRM |  | Alkylation | C | IAA |  | + |  | + | VM |
| Q71U36-2 | Tubulin alpha-1A chain | TUBA1A | IATIKTKRTIQFVDWCPTGFKVGINYQPPTV |  | Alkylation | C | IAA | + | + |  |  | VM |
| P68363 | Tubulin alpha-1B chain | TUBA1B | IAAIKTKRSIQFVDWCPTGFKVGINYQPPTV;IATIKTKRSIQFVDWCPTGFKVGINYQPPTV |  | Alkylation | C | IAA | + | + |  |  | VM |
| P68363 | Tubulin alpha-1A chain | TUBA1A | TVVPGGDLAKVQRAVCMLSNTTAIAEAWARL |  | Alkylation | C | IAA | + |  |  |  | VM |
| P04350 | Tubulin beta chain | TUBB | DLNHLVSATMSGVTTCLRFPGQLNADLRKLA |  | Alkylation | C | IAA |  | + |  |  | VM |
| H7C1J4 | UHRF1-binding protein 1 | UHRF1BP1 | NQGRIQIALKRRTKDCNVISSKLMFLLDDLL |  | Alkylation | C | IAA | + |  |  |  | VM |
| P31946-2 | 14-3-3 protein beta/alpha | YWHAB | GKEYREKIEAELQDICNDVLELLDKYLIPNA |  | Alkylation | C | IAA | + | + |  |  | VM |
| P62258 | 14-3-3 protein epsilon | YWHAE | IREYRQMVETELKLICCDILDVLDKHLIPAA |  | Alkylation | C | IAA | + | + |  |  | VM |
| P62258 | 14-3-3 protein epsilon | YWHAE | REYRQMVETELKLICCDILDVLDKHLIPAAN |  | Alkylation | C | IAA | + | + |  |  | VM |
| P61981 | 14-3-3 protein gamma | YWHAG | VRAYREKIEKELEAVCQDVLSLLDNYLIKNC |  | Alkylation | C | IAA | + | + |  |  | VM |
| P63104 | 14-3-3 protein zeta/delta | YWHAZ | AREYREKIETELRDICNDVLSLLEKFLIPNA |  | Alkylation | C | IAA |  | + |  |  | VM |
| X6RCN5 | Zinc finger MYM-type protein 6 | ZMYM6 | KGQTAYHKTGSTQLFCSTRCITRHSSPACLP |  | Alkylation | C | IAA |  |  | + |  | VM |
| X6RCN5 | Zinc finger MYM-type protein 6 | ZMYM6 | AYHKTGSTQLFCSTRCITRHSSPACLPPPPK |  | Alkylation | C | IAA |  |  | + |  | VM |
| E9PL27 | Zinc finger protein 790 | ZNF790 | AQLEIMRICKNHSLDCLCFRGDWEGNTQFQT |  | Alkylation | C | IAA |  |  | + | + | VM |
| E9PL27 | Zinc finger protein 790 | ZNF790 | LEIMRICKNHSLDCLCFRGDWEGNTQFQTLQ |  | Alkylation | C | IAA |  |  | + | + | VM |
| Q05996-2 | Processed zona pellucida sperm-binding protein 2 | ZP2 | ILDPEKLTLRATYDNCTRRVHGGHQMTIRVM |  | Alkylation | C | IAA | + | + | + | + | VM |
| A0A1W2PS94 |  |  | LRFLSTGFPKRAERTCSLNNCTVAKRFGKGK |  | Alkylation | C | IAA |  |  | + |  | VM |
| A0A1W2PS94 |  |  | TGFPKRAERTCSLNNCTVAKRFGKGKDATVT |  | Alkylation | C | IAA |  |  | + |  | VM |
| A0A0U1RRH9 | Septin-7 | SEPT7 | FEDYLNAESRVNRRQMPDNRVQCCLYFIAPS |  | Sulfoxide | M | MO |  |  | + |  | VM |
| P68133 | Actin, alpha cardiac muscle 1 | ACTA1 | LKYPIEHGIITNWDDMEKIWHHTFYNELRVA |  | Sulfoxide | M | MO | + | + | + | + | VM |
| P63261 | Actin, cytoplasmic 1 | ACTB | RKDLYANTVLSGGTTMYPGIADRMQKEITAL |  | Sulfoxide | M | MO | + |  |  |  | VM |
| P63261 | Actin, alpha cardiac muscle 1 | ACTA1 | AVFPSIVGRPRHQGVMVGMGQKDSYVGDEAQ;AVFPSMIGRPRHQGVMVGMGQKDCYVGDEAQ |  | Sulfoxide | M | MO |  |  | + | + | VM |
| P63261 | Actin, alpha cardiac muscle 1 | ACTA1 | PSIVGRPRHQGVMVGMGQKDSYVGDEAQSKR;PSMIGRPRHQGVMVGMGQKDCYVGDEAQSKR |  | Sulfoxide | M | MO |  |  | + | + | VM |
| P63261 | Actin, alpha cardiac muscle 1 | ACTA1 | AVFPSIVGRPRHQGVMVGMGQKDSYVGDEAQ;AVFPSMIGRPRHQGVMVGMGQKDCYVGDEAQ |  | Sulfoxide | M | MO | + | + | + | + | VM |
| P63261 | Actin, alpha cardiac muscle 1 | ACTA1 | PSIVGRPRHQGVMVGMGQKDSYVGDEAQSKR;PSMIGRPRHQGVMVGMGQKDCYVGDEAQSKR |  | Sulfoxide | M | MO | + | + | + | + | VM |
| P63261 | Actin | ACTA1 | ADRMQKEITALAPSTMKIKIIAPPERKYSVW |  | Sulfoxide | M | MO |  | + |  | + | VM |
| P12814 | Alpha-actinin-1 | ACTN1 | WIQTKMEEIGRISIEMHGTLEDQLSHLRQYE |  | Sulfoxide | M | MO |  |  |  | + | VM |
| P06576 | ATP synthase subunit beta, mitochondrial | ATP5B | LQDYKSLQDIIAILGMDELSEEDKLTVSRAR |  | Sulfoxide | M | MO | + | + |  |  | VM |
| Q9UQM7 | Calcium/calmodulin-dependent protein kinase type II subunit alpha | CAMK2A | LIEAISNGDFESYTKMCDPGMTAFEPEALGN |  | Sulfoxide | M | MO | + |  |  |  | VM |
| P12277 | Creatine kinase B-type | CKB | SNADRLGFSEVELVQMVVDGVKLLIEMEQRL |  | Sulfoxide | M | MO | + |  |  |  | VM |
| A0A087WVQ6 | Clathrin heavy chain | CLTC | INYYQDRGYFEELITMLEAALGLERAHMGMF |  | Sulfoxide | M | MO |  | + |  |  | VM |
| Q12860-2 | Contactin-1 | CNTN1 | LSYRWLLNEFPVFITMDKRRFVSQTNGNLYI |  | Sulfoxide | M | MO | + |  |  |  | VM |
| P08311 | Cathepsin G | CTSG | EIIGGRESRPHSRPYMAYLQIQSPAGQSRCG |  | Sulfoxide | M | MO |  | + |  |  | VM |
| P08311 | Cathepsin G | CTSG | IRHPQYNQRTIQNDIMLLQLSRRVRRNRNVN |  | Sulfoxide | M | MO |  | + |  | + | VM |
| C9JC03 | EH domain-containing protein 1 | EHD1 | KTTFIRHLIEQDFPGMRIGPEPTTDSFIAVM |  | Sulfoxide | M | MO | + | + | + | + | VM |
| H0YAP7 | Elongator complex protein 3 | ELP3 | VSSRDPTKFQHQGFGMLLMEEAERIAREEHG |  | Sulfoxide | M | MO |  |  |  | + | VM |
| H0YAP7 | Elongator complex protein 3 | ELP3 | RDPTKFQHQGFGMLLMEEAERIAREEHGSGK |  | Sulfoxide | M | MO |  |  |  | + | VM |
| P06733 | Alpha-enolase | ENO1 | SKKLNVTEQEKIDKLMIEMDGTENKSKFGAN |  | Sulfoxide | M | MO |  | + |  |  | VM |
| P06733 | Alpha-enolase | ENO1 | LNVTEQEKIDKLMIEMDGTENKSKFGANAIL |  | Sulfoxide | M | MO |  | + |  |  | VM |
| G5E9W8 | Glycogenin-1 | GYG1 | TRRLVVLATPQVSDSMRKVLETVFDEVIMVD |  | Sulfoxide | M | MO | + | + | + | + | VM |
| U3KQK0 | Histone H2B | H2BFS | VLKQVHPDTGISSKAMGIMNSFVNDIFERIA |  | Sulfoxide | M | MO | + | + | + | + | VM |
| U3KQK0 | Histone H2B | H2BFS | QVHPDTGISSKAMGIMNSFVNDIFERIAGEA |  | Sulfoxide | M | MO | + | + | + | + | VM |
| U3KQK0 | Histone H2B | H2BFS | VLKQVHPDTGISSKAMGIMNSFVNDIFERIA |  | Sulfoxide | M | MO | + | + | + | + | VM |
| U3KQK0 | Histone H2B | H2BFS | QVHPDTGISSKAMGIMNSFVNDIFERIAGEA |  | Sulfoxide | M | MO | + | + | + | + | VM |
| D6RFF0 | La-related protein 7 | LARP7 | GRMKKEDNIQAKEENMDTSNTSISKMKRSRP |  | Sulfoxide | M | MO | + | + | + |  | VM |
| H0YCA4 | Low-density lipoprotein receptor-related protein 8 | LRP8 | GYLIWRNWKRKNTKSMNFDNPVYRKTTEEED |  | Sulfoxide | M | MO | + | + | + | + | VM |
| P05164-2 | 84 kDa myeloperoxidase | MPO | RDGDRFWWENEGVFSMQQRQALAQISLPRII |  | Sulfoxide | M | MO | + | + | + | + | VM |
| P05164-2 | 84 kDa myeloperoxidase | MPO | ITYRDYLPLVLGPTAMRKYLPTYRSYNDSVD |  | Sulfoxide | M | MO |  | + |  |  | VM |
| P05164-2 | 84 kDa myeloperoxidase | MPO | QIAVDEIRERLFEQVMRIGLDLPALNMQRSR |  | Sulfoxide | M | MO | + | + | + | + | VM |
| P05164-2 | 84 kDa myeloperoxidase | MPO | FEQVMRIGLDLPALNMQRSRDHGLPGYNAWR |  | Sulfoxide | M | MO | + | + | + | + | VM |
| P05164-2 | 84 kDa myeloperoxidase | MPO | DGERLYQEARKIVGAMVQIITYRDYLPLVLG |  | Sulfoxide | M | MO |  | + | + | + | VM |
| P05164-2 | 84 kDa myeloperoxidase | MPO | CDNTGITTVSKNNIFMSNSYPRDFVNCSTLP |  | Sulfoxide | M | MO | + | + | + | + | VM |
| P05164-2 | 84 kDa myeloperoxidase | MPO | IRNQINALTSFVDASMVYGSEEPLARNLRNM |  | Sulfoxide | M | MO |  |  |  | + | VM |
| P05164-2 | 84 kDa myeloperoxidase | MPO | RFPTDQLTPDQERSLMFMQWGQLLDHDLDFT |  | Sulfoxide | M | MO |  | + | + | + | VM |
| P05164-2 | 84 kDa myeloperoxidase | MPO | ARIPCFLAGDTRSSEMPELTSMHTLLLREHN |  | Sulfoxide | M | MO |  | + | + | + | VM |
| P05164-2 | 84 kDa myeloperoxidase | MPO | LAGDTRSSEMPELTSMHTLLLREHNRLATEL |  | Sulfoxide | M | MO |  |  |  | + | VM |
| P05164-2 | 84 kDa myeloperoxidase | MPO | FTNAFRYGHTLIQPFMFRLDNRYQPMEPNPR |  | Sulfoxide | M | MO |  | + |  | + | VM |
| P05164-2 | 84 kDa myeloperoxidase | MPO | QLGTVLRNLKLARKLMEQYGTPNNIDIWMGG |  | Sulfoxide | M | MO |  |  |  | + | VM |
| P05164-2 | 84 kDa myeloperoxidase | MPO | KLMEQYGTPNNIDIWMGGVSEPLKRKGRVGP |  | Sulfoxide | M | MO |  |  |  | + | VM |
| P05164-2 | 84 kDa myeloperoxidase | MPO | RFPTDQLTPDQERSLMFMQWGQLLDHDLDFT |  | Sulfoxide | M | MO | + | + | + | + | VM |
| P05164-2 | 84 kDa myeloperoxidase | MPO | PTDQLTPDQERSLMFMQWGQLLDHDLDFTPE |  | Sulfoxide | M | MO | + | + | + | + | VM |
| P05164-2 | 84 kDa myeloperoxidase | MPO | ARIPCFLAGDTRSSEMPELTSMHTLLLREHN |  | Sulfoxide | M | MO |  | + |  | + | VM |
| P05164-2 | 84 kDa myeloperoxidase | MPO | LAGDTRSSEMPELTSMHTLLLREHNRLATEL |  | Sulfoxide | M | MO |  | + |  | + | VM |
| P35579 | Myosin-9 | MYH9 | EKSKSLAKLKNKHEAMITDLEERLRREEKQR |  | Sulfoxide | M | MO |  |  |  | + | VM |
| F8VZU9 | Myosin light polypeptide 6 | MYL6 | AEMNVKVLDFEHFLPMLQTVAKNKDQGTYED |  | Sulfoxide | M | MO |  |  |  | + | VM |
| E5RI98 | Nucleophosmin | NPM1 | NYEGSPIKVTLATLKMSVQPTVSLGGFEITP |  | Sulfoxide | M | MO | + |  |  |  | VM |
| P07737 | Profilin-1 | PFN1 | CSVIRDSLLQDGEFSMDLRTKSTGGAPTFNV |  | Sulfoxide | M | MO |  | + |  | + | VM |
| P18669 | Phosphoglycerate mutase 1 | PGAM1 | RAIRTLWTVLDAIDQMWLPVVRTWRLNERHY |  | Sulfoxide | M | MO |  | + |  |  | VM |
| P60201 | Myelin proteolipid protein | PLP1 | SKTSASIGSLCADARMYGVLPWNAFPGKVCG |  | Sulfoxide | M | MO | + | + |  |  | VM |
| E9PLB5 | Protein phosphatase 1 regulatory subunit 36 | PPP1R36 | FYARRKRLGGQTPYLMDQLGLRLGMWYWKDE |  | Sulfoxide | M | MO |  | + | + | + | VM |
| P05109 | Protein S100-A8 | S100A8 | _______________MLTELEKALNSIIDVY |  | Sulfoxide | M | MO | + | + | + | + | VM |
| P06702 | Protein S100-A9 | S100A9 | FIMLMARLTWASHEKMHEGDEGPGHHHKPGL |  | Sulfoxide | M | MO | + | + | + | + | VM |
| P06702 | Protein S100-A9 | S100A9 | FLKKENKNEKVIEHIMEDLDTNADKQLSFEE |  | Sulfoxide | M | MO | + | + | + | + | VM |
| C9J7N5 | Serpin I2 | SERPINI2 | ISTWVERKTDGKIKDMFSGEEFGPLTRLVLV |  | Sulfoxide | M | MO |  | + | + | + | VM |
| P68363 | Tubulin alpha-1A chain | TUBA1A | MYAKRAFVHWYVGEGMEEGEFSEAREDMAAL |  | Sulfoxide | M | MO | + | + |  |  | VM |
| Q5JP53 | Tubulin beta chain | TUBB | KYVPRAILVDLEPGTMDSVRSGPFGQIFRPD |  | Sulfoxide | M | MO | + |  |  |  | VM |
| P04350 | Tubulin beta chain | TUBB | TLLISKIREEFPDRIMNTFSVVPSPKVSDTV;TLLISKIREEYPDRIMNTFSVVPSPKVSDTV |  | Sulfoxide | M | MO |  | + |  |  | VM |
| P04350 | Tubulin beta chain | TUBB | FPGQLNADLRKLAVNMVPFPRLHFFMPGFAP |  | Sulfoxide | M | MO | + |  |  |  | VM |
| P68371 | Tubulin beta-2A chain | TUBB2A | NVKTAVCDIPPRGLKMSATFIGNSTAIQELF |  | Sulfoxide | M | MO | + | + |  |  | VM |
| P10599 | Thioredoxin | TXN | LVVVDFSATWCGPCKMIKPFFHSLSEKYSNV |  | Sulfoxide | M | MO | + | + |  |  | VM |
| B0YJC4 | Vimentin | VIM | KGQGKSRLGDLYEEEMRELRRQVDQLTNDKA |  | Sulfoxide | M | MO | + | + | + |  | VM |
| Q6PP77-2 | XK-related protein 2 | XKRX | ILFEPWIKFWRSGAQMPNNIEKNFSRVGTLV |  | Sulfoxide | M | MO | + |  | + | + | VM |
| P31946-2 | 14-3-3 protein beta/alpha | SFN | LDTLNEDSYKDSTLIMQLLRDNLTLWTSDQQ;LDTLNEESYKDSTLIMQLLRDNLTLWTSENQ;LDTLSEESYKDSTLIMQLLRDNLTLWTSDMQ;LDTLSEESYKDSTLIMQLLRDNLTLWTSDTQ |  | Sulfoxide | M | MO | + |  |  |  | VM |
| O14862 | Interferon-inducible protein AIM2 | AIM2 | RIFQKLNYMLLAKRLQEEKEKVDKQYKSVTK |  | Deamination | Q | NQ | + | + | + | + | VM |
| A0A0C4DGD1 | Calpastatin | CAST | RDDTIPPEYRHLLDDNGQDKPVKPPTKKSED |  | Deamination | N | NQ | + |  |  |  | VM |
| H3BTW5 | C-myc promoter-binding protein | DENND4A | PPLQYSYNGFPVLRNNLFERPEGFLQAKKNK |  | Deamination | N | NQ | + | + | + | + | VM |
| H3BTW5 | C-myc promoter-binding protein | DENND4A | EPPLQYSYNGFPVLRNNLFERPEGFLQAKKN |  | Deamination | N | NQ | + | + | + | + | VM |
| H3BTW5 | C-myc promoter-binding protein | DENND4A | PPLQYSYNGFPVLRNNLFERPEGFLQAKKNK |  | Deamination | N | NQ | + | + | + | + | VM |
| H7C4R7 | Deoxyribonuclease | DNASE1L3 | WLIGDQEDTTVKKSTNCAYDRPWMSATTFQL |  | Deamination | N | NQ | + | + | + | + | VM |
| H0YAP7 | Elongator complex protein 3 | ELP3 | YGSVVPVSSRDPTKFQHQGFGMLLMEEAERI |  | Deamination | Q | NQ |  |  |  | + | VM |
| P04406-2 | Glyceraldehyde-3-phosphate dehydrogenase | GAPDH | STGAAKAVGKVIPELNGKLTGMAFRVPTANV |  | Deamination | N | NQ |  | + |  |  | VM |
| U3KQK0 | Histone H2B | H2BFS | VHPDTGISSKAMGIMNSFVNDIFERIAGEAS |  | Deamination | N | NQ | + | + |  | + | VM |
| U3KQK0 | Histone H2B | H2BFS | TGISSKAMGIMNSFVNDIFERIAGEASRLAH |  | Deamination | N | NQ | + | + |  | + | VM |
| U3KQK0 | Histone H2B | H2BFS | SRKESYSVYVYKVLKQVHPDTGISSKAMGIM |  | Deamination | Q | NQ | + | + | + | + | VM |
| A0A140TA05 | HLA class I histocompatibility antigen, Cw-7 alpha chain | HLA-C | AQITQRKLEAARAAEQLRAYLEGTCVEWLRR |  | Deamination | Q | NQ |  |  | + |  | VM |
| P05204 | Non-histone chromosomal protein HMG-17 | HMGN2 | KGKADAGKEGNNPAENGDAKTDQAQKAEGAG |  | Deamination | N | NQ | + | + | + |  | VM |
| D6RFF0 | La-related protein 7 | LARP7 | KEDNIQAKEENMDTSNTSISKMKRSRPTSEG |  | Deamination | N | NQ | + | + | + |  | VM |
| H0YCA4 | Low-density lipoprotein receptor-related protein 8 | LRP8 | WRNWKRKNTKSMNFDNPVYRKTTEEEDEDEL |  | Deamination | N | NQ | + | + | + | + | VM |
| P05164-2 | 84 kDa myeloperoxidase | MPO | TQFRKLRDGDRFWWENEGVFSMQQRQALAQI |  | Deamination | N | NQ |  |  |  | + | VM |
| P05164-2 | 84 kDa myeloperoxidase | MPO | LFEQVMRIGLDLPALNMQRSRDHGLPGYNAW |  | Deamination | N | NQ |  | + |  | + | VM |
| P05164-2 | 84 kDa myeloperoxidase | MPO | NTGITTVSKNNIFMSNSYPRDFVNCSTLPAL |  | Deamination | N | NQ |  | + |  |  | VM |
| P05164-2 | 84 kDa myeloperoxidase | MPO | DGFSLPYGWTPGVKRNGFPVALARAVSNEIV |  | Deamination | N | NQ |  |  |  | + | VM |
| P05164-2 | 84 kDa myeloperoxidase | MPO | NTGITTVSKNNIFMSNSYPRDFVNCSTLPAL |  | Deamination | N | NQ |  | + |  |  | VM |
| Q9Y6Q9-4 | Nuclear receptor coactivator 3 | NCOA3 | NIMRPRTNTPKQLRMQLQQRLQGQQFLNQSR |  | Deamination | Q | NQ |  | + | + |  | VM |
| Q9Y6Q9-4 | Nuclear receptor coactivator 3 | NCOA3 | MRPRTNTPKQLRMQLQQRLQGQQFLNQSRQA |  | Deamination | Q | NQ |  | + | + |  | VM |
| Q9Y6Q9-4 | Nuclear receptor coactivator 3 | NCOA3 | RPRTNTPKQLRMQLQQRLQGQQFLNQSRQAL |  | Deamination | Q | NQ |  | + | + |  | VM |
| P00558-2 | Phosphoglycerate kinase 1 | PGK1 | KKYAEAVTRAKQIVWNGPVGVFEWEAFARGT |  | Deamination | N | NQ |  | + |  |  | VM |
| H3BTN5 | Pyruvate kinase | PKM | QVKQKGADFLVTEVENGGSLGSKKGVNLPGA |  | Deamination | N | NQ | + |  |  |  | VM |
| B9ZVN9 | DNA-directed RNA polymerase | POLR1A | VKRQRIIEESTHCGPQAVRAALNLPEAASYD |  | Deamination | Q | NQ |  | + |  |  | VM |
| E9PLB5 | Protein phosphatase 1 regulatory subunit 36 | PPP1R36 | YRVPEFYARRKRLGGQTPYLMDQLGLRLGMW |  | Deamination | Q | NQ |  | + | + | + | VM |
| C9IYJ2 | PX domain-containing protein kinase-like protein | PXK | FQEYIIRVQRGISVENSWQIVRRYSDFDLLN |  | Deamination | N | NQ |  | + |  |  | VM |
| C9IYJ2 | PX domain-containing protein kinase-like protein | PXK | YIIRVQRGISVENSWQIVRRYSDFDLLNNSL |  | Deamination | Q | NQ |  | + |  |  | VM |
| E9PK47 | Alpha-1,4 glucan phosphorylase | PYGL | PNDFNLRDFNVGDYIQAVLDRNLAENISRVL |  | Deamination | Q | NQ | + | + |  |  | VM |
| A0A0A0MRV0 | Ribosome-binding protein 1 | RRBP1 | HKATQKGDPVAILKRQLEEKEKLLATEQEDA |  | Deamination | Q | NQ |  | + | + | + | VM |
| P06702 | Protein S100-A9 | S100A9 | TFHQYSVKLGHPDTLNQGEFKELVRKDLQNF |  | Deamination | N | NQ |  | + |  | + | VM |
| P06702 | Protein S100-A9 | S100A9 | KNEKVIEHIMEDLDTNADKQLSFEEFIMLMA |  | Deamination | N | NQ | + | + | + | + | VM |
| P06702 | Protein S100-A9 | S100A9 | TLNQGEFKELVRKDLQNFLKKENKNEKVIEH |  | Deamination | Q | NQ |  |  |  | + | VM |
| P30740 | Leukocyte elastase inhibitor | SERPINB1 | TRGNTAAQLSKTFHFNTVEEVHSRFQSLNAD |  | Deamination | N | NQ |  | + |  | + | VM |
| C9JNW0 | SH3 domain-binding protein 5 | SH3BP5 | _____________MLNHATQRVMEAEQTKTR |  | Deamination | N | NQ |  |  | + |  | VM |
| C9JNW0 | SH3 domain-binding protein 5 | SH3BP5 | _________MLNHATQRVMEAEQTKTRSELV |  | Deamination | Q | NQ |  |  | + |  | VM |
| P43004-3 | Amino acid transporter | SLC1A2 | PGNPKLKKQLGPGKKNDEVSSLDAFLDLIRN |  | Deamination | N | NQ | + | + |  |  | VM |
| Q9H156 | SLIT and NTRK-like protein 2 | SLITRK2 | LPKRQFAPSYESRRQNQDRINKTVLYGTPRK |  | Deamination | N | NQ | + | + | + | + | VM |
| Q9H156 | SLIT and NTRK-like protein 2 | SLITRK2 | FAPSYESRRQNQDRINKTVLYGTPRKCFVGQ |  | Deamination | N | NQ |  |  |  | + | VM |
| Q9H156 | SLIT and NTRK-like protein 2 | SLITRK2 | TLPKRQFAPSYESRRQNQDRINKTVLYGTPR |  | Deamination | Q | NQ | + | + | + | + | VM |
| Q9H156 | SLIT and NTRK-like protein 2 | SLITRK2 | PKRQFAPSYESRRQNQDRINKTVLYGTPRKC |  | Deamination | Q | NQ | + | + | + | + | VM |
| Q9H156 | SLIT and NTRK-like protein 2 | SLITRK2 | LPKRQFAPSYESRRQNQDRINKTVLYGTPRK |  | Deamination | N | NQ | + | + | + | + | VM |
| Q9H156 | SLIT and NTRK-like protein 2 | SLITRK2 | FAPSYESRRQNQDRINKTVLYGTPRKCFVGQ |  | Deamination | N | NQ | + |  | + | + | VM |
| Q9H156 | SLIT and NTRK-like protein 2 | SLITRK2 | TLPKRQFAPSYESRRQNQDRINKTVLYGTPR |  | Deamination | Q | NQ | + | + | + | + | VM |
| Q9H156 | SLIT and NTRK-like protein 2 | SLITRK2 | PKRQFAPSYESRRQNQDRINKTVLYGTPRKC |  | Deamination | Q | NQ | + | + | + | + | VM |
| C9JW66 | Synaptojanin-1 | SYNJ1 | KPQVQKFLDYGFFYFNGSEVQRCQSGTVRTN |  | Deamination | N | NQ |  | + |  |  | VM |
| Q6PP77-2 | XK-related protein 2 | XKRX | FEPWIKFWRSGAQMPNNIEKNFSRVGTLVVL |  | Deamination | N | NQ | + |  | + | + | VM |
| Q6PP77-2 | XK-related protein 2 | XKRX | EPWIKFWRSGAQMPNNIEKNFSRVGTLVVLI |  | Deamination | N | NQ | + |  | + | + | VM |
| Q6PP77-2 | XK-related protein 2 | XKRX | IILFEPWIKFWRSGAQMPNNIEKNFSRVGTL |  | Deamination | Q | NQ | + |  | + | + | VM |
| O00254-2 | Proteinase-activated receptor 3 | F2RL2 | _____________MENDTNNLAKPTLPIKTF |  | Deamination | N | NQ | + | + | + | + | VM |
| O00254-2 | Proteinase-activated receptor 3 | F2RL2 | __________MENDTNNLAKPTLPIKTFRGA |  | Deamination | N | NQ | + | + | + | + | VM |
| O00254-2 | Proteinase-activated receptor 3 | F2RL2 | _________MENDTNNLAKPTLPIKTFRGAP |  | Deamination | N | NQ | + | + | + | + | VM |
| O00254-2 | Proteinase-activated receptor 3 | F2RL2 | _____________MENDTNNLAKPTLPIKTF |  | Deamination | N | NQ |  | + |  |  | VM |
| O00254-2 | Proteinase-activated receptor 3 | F2RL2 | __________MENDTNNLAKPTLPIKTFRGA |  | Deamination | N | NQ |  | + |  |  | VM |
| O00254-2 | Proteinase-activated receptor 3 | F2RL2 | _________MENDTNNLAKPTLPIKTFRGAP |  | Deamination | N | NQ |  | + |  |  | VM |
